# Intramolecular Reaction of Transient Phosphenium and Arsenium Ions Giving Rise to Isolable 9‐Phospha‐ and 9‐Arsena‐Fluorenium Ions

**DOI:** 10.1002/anie.202006728

**Published:** 2020-07-06

**Authors:** Marian Olaru, Daniel Duvinage, Yannik Naß, Lorraine A. Malaspina, Stefan Mebs, Jens Beckmann

**Affiliations:** ^1^ Institut für Anorganische Chemie und Kristallographie Universität Bremen Leobener Straße 7 28359 Bremen Germany; ^2^ Institut für Experimentalphysik Freie Universität Berlin Arnimallee 14 14195 Berlin Germany

**Keywords:** arenium ions, arsoles, *m*-terphenyl compounds, phospholes, pnictogenium ions

## Abstract

Transient phosphenium and arsenium ions, generated by fluoride abstraction from bis(*m*‐terphenyl)fluoropnictogens, underwent intramolecular electrophilic attack prior to methyl group migration and gave rise to isolable 9‐phospha‐ and 9‐arsena‐fluorenium ions.

## Graphical abstract

**Chemical rollercoaster ride**: Attempts to prepare phosphenium and arsenium ions of the type [(2,6‐{Me_5_C_6_}_2_C_6_H_3_)_2_E]^+^ (E=P; E=As) lead to the isolation of two‐isomer mixtures of phospha‐ and arsena‐fluorenium ions. These arenium ions were isolated and structurally characterized. They are high‐energy species that undergo subsequent, solvent‐dependent reactions leading to thermodynamically favored products.

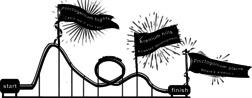

Arenium ions are well‐recognized key intermediates of electrophilic aromatic substitution reactions.^1^ Although often referred to as Wheland intermediates due to his seminal theoretical publication of 1942,^2^ the interest in arenium ions dates back earlier to pioneering experimental work by Pfeiffer and Wizinger, published already in 1928.^3^ Over the years a number of arenium ions have been isolated and fully characterized (Scheme 1). For instance, the protonation of benzene provided the parent benzenium ion [C_6_H_7_]^+^ (**I**).^4^ Starting from hexamethylbenzene, substituted hexamethylbenzenium ions [C_6_Me_6_R]^+^ (**II**, R=Me,^5^ Cl,^6^ Br^7^) were obtained. Attempts to afford silyl cations [R_3_Si]^+^ have led to the isolation of silyl‐substituted toluenium ions [C_6_H_5_MeSiR_3_]^+^ (**III**, R=Me, Et).^8^

**Scheme 1:**
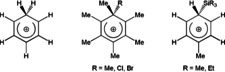
Examples of previously known arenium ions.

In their native form, phosphenium ions [R_2_P]^+^ and arsenium ions [R_2_As]^+^ are highly reactive six‐valence‐electron species that have never been isolated without the support of electron‐rich ligands.^9^ Our efforts to prepare kinetically stabilized pnictogenium ions [(2,6‐Mes_2_C_6_H_3_)_2_E]^+[10]^ by fluoride abstraction from bis‐*m*‐terphenylpnictogen fluorides (2,6‐Mes_2_C_6_H_3_)_2_EF (E=P, As) yielded 1,2,4‐trimethyl‐6‐mesityl‐5‐*m*‐terphenylbenzo[b]phospholium and ‐arsolium ions instead.^11^ The formation of these protonated phosphole and arsole structures involved a methyl group migration from the *ortho*‐position of a flanking mesityl group to the *meta*‐position prior to a hydrogen transfer from that position to phosphorus and arsenic. For the mechanism of this rearrangement we proposed transient short‐lived arenium ions. We further concluded that reactive hydrogen atoms need to be avoided in the *m*‐terphenyl substituents in order to isolate kinetically stabilized phosphenium and arsenium ions by this route.^11^

We have therefore adjusted our strategy and turned our attention to a related *m*‐terphenyl substituent in which the two flanking aryl groups are permethylated. Starting from 2,6‐(Me_5_C_6_)_2_C_6_H_3_Li^12^ and ECl_3_ (E=P, As), we have now prepared the bis(*m*‐terphenyl)fluoropnictogens (2,6‐{Me_5_C_6_}_2_C_6_H_3_)_2_EF (**1 a**, E=P; **1 b**, E=As). The synthesis, solution NMR, and solid‐state structural features of **1 a** and **1 b** resemble those of (2,6‐Mes_2_C_6_H_3_)_2_EF (E=P,^13^ As^11^) reported previously. In solution, **1 a** and **1 b** are characterized by ^19^F NMR chemical shifts (CD_2_Cl_2_) at *δ*=−200.3 (E=P) and −216.0 ppm (E=As), while **1 a** gives a ^31^P NMR signal at *δ*=193.8 ppm. Fluoride ion abstraction from **1 a** and **1 b** using EtAlCl_2_ and AlCl_3_, respectively, in CH_2_Cl_2_ proceeded in virtually quantitative yield to afford two‐isomer mixtures of phospha‐fluorenium ions [**5 a**]^+^ and [**7 a**]^+^ (ca. 0.65:0.35 molar ratio) and arsa‐fluorenium ions [**5 b**]^+^ and [**7 b**]^+^ (ca. 0.70:0.30 molar ratio), species isolated as dark‐red crystalline solids (Figure 1). The ^31^P NMR spectrum (CD_2_Cl_2_) showing two close resonances at *δ*=−26.9 ppm for [**5 a**]^+^ and −22.2 ppm for [**7 a**]^+^ confirmed that phosphorus was in a very similar coordination environment for both isomers. The ^1^H NMR spectra of these mixtures were complex; as a consequence of complete desymmetrization of the molecules, each species displayed six signals assigned to the aromatic protons and individual resonances for all methyl groups in the aliphatic area which only overlapped by chance. The similar distribution pattern of the resonances and of the coupling constants indicated that the major and minor species were closely related isomers. In both the phosphorus and arsenic cases, the ^1^H NMR spectra displayed sharp signals with virtually no change when measured at lower temperatures, signaling configurationally stable structures. NOESY spectra did not reveal any exchange between the major and the minor species in either phosphorus or arsenic cases, even at very short mixing times (see the Supporting Information).^15^ The identities of the major phosphole [**5 a**]^+^ and arsole [**5 b**]^+^ components of the mixtures were confirmed by single‐crystal X‐ray diffraction (Figure 1),^14^ and after the full NMR assignment it was clear that the solid‐state structures were retained in solution. The molecular structures of [**5 a**]^+^ and [**5 b**]^+^ reveal that after abstraction of the fluoride ion, the corresponding pnictogenium ions [(2,6‐{Me_5_C_6_}_2_C_6_H_3_)_2_E]^+^ ([**2 a**]^+^, E=P; [**2 b**]^+^, E=As) immediately underwent intramolecular electrophilic substitution, which caused the displacement of one of the original *ortho*‐methyl groups of a flanking C_6_Me_5_ group and formation of arenium ions. This is reminiscent of similarly behaving unstable cations [(2,6‐Mes_2_C_6_H_3_)_2_E]^+^ (E=P, As), which were previously shown to undergo a 1,2‐methyl shift, resulting in formation of protonated phospholium and arsolium ions.^11^ The molecular structures of [[**5 a**] and [**5 b**]^+^ compare well with those of the related phospholes and arsoles,^11^ with very similar bond lengths and angles around the pnictogen atoms. The dearomatized rings in [**5 a**]^+^ and [**5 b**]^+^ contain two single C−C bonds (C22,24−C23 1.499(3) Å for both [**5 a**] and [**5 b**]^+^) while the rest of the C−C bond lengths range between 1.359(3) Å (C21−C22, [**5 a**]^+^) and 1.436(2) Å (C20−C25, [**5 b**]^+^). Despite significant efforts we could not obtain suitable single crystals of [**7 a**]^+^ and [**7 b**]^+^ but the solution structures were unambiguously inferred from NMR spectroscopy and identified as positional isomers resulting from the migration of the displaced methyl group in a position *ortho* relative to the central aromatic ring.

**Figure 1:**
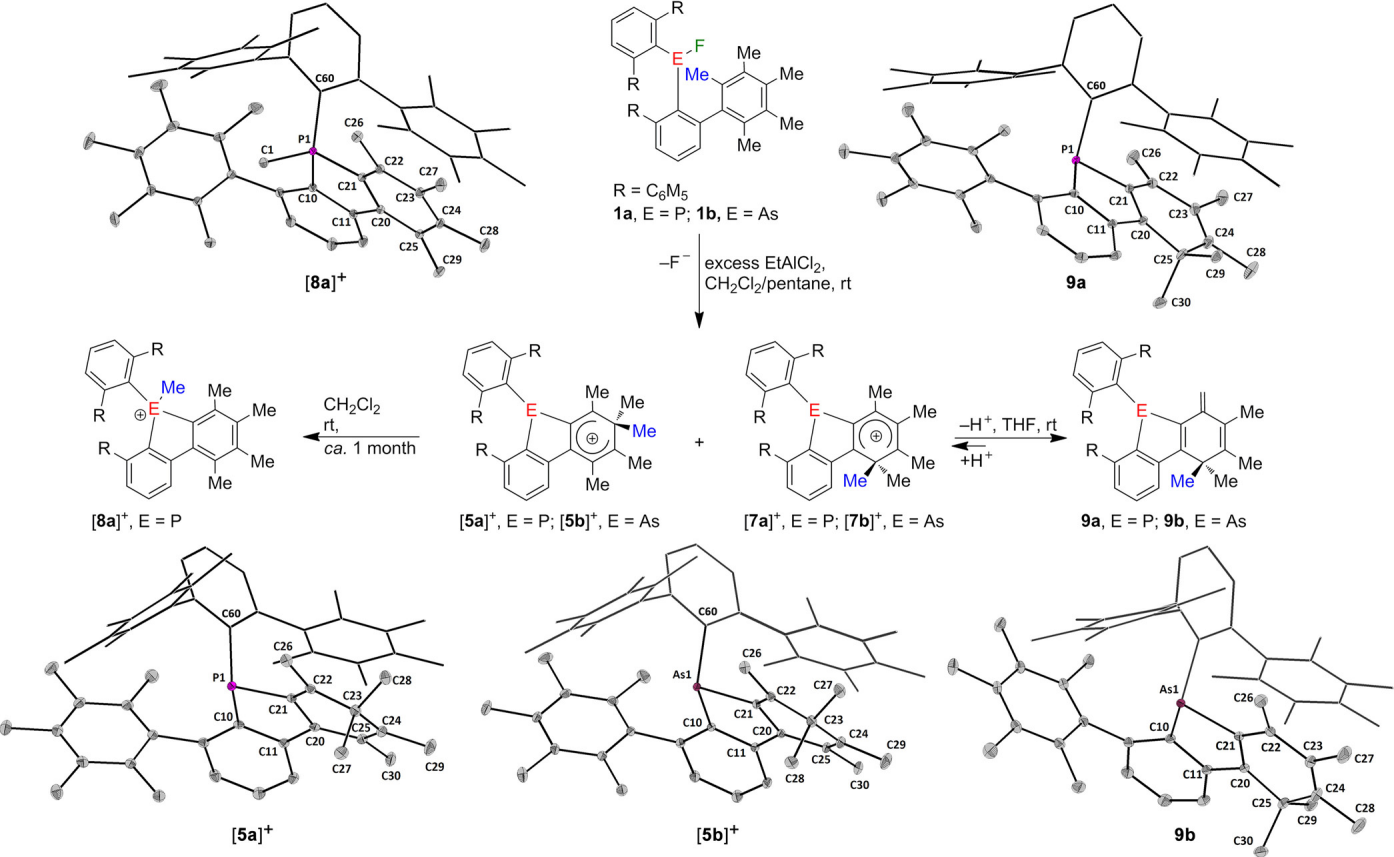
Fluoride abstraction from bis(*m*‐terphenyl)element fluorides **1 a** and **1 b** and subsequent reactions of arenium ions [**5 a**]^+^ and [**5 b**]^+^ as well as [**7 a**]^+^ and [**7 b**]^+^. Molecular structures of [**5 a**]^+^, [**5 b**]^+^, [**8 a**]^+^, **9 a**, and **9 b** showing 30 % probability ellipsoids and the essential atom numbering scheme. For clarity, the counter ions of [**5 a**]^+^ ([0.75⋅AlCl_4_+0.25⋅EtAlCl_3_]^−^), [**5 b**]^+^ ([AlCl_4_]^−^), and [**8 a**]^+^ ([AlCl_4_]^−^) were omitted from both the reaction scheme and structure depictions. Selected bond parameters [Å, °] of [**5 a**]^+^: P1‐C10 1.831(2), P1‐C21 1.836(2), P1‐C60 1.861(2), C20‐C21 1.432(3), C21‐C22 1.359(3), C22‐C23 1.500(3), C24‐C23 1.499(3), C25‐C24 1.370(3), C20‐C25 1.429(3), C10‐P1‐C21 88.40(8), C10‐P1‐C60 104.77(8), C21‐P1‐C60 107.63(8), C28‐C23‐C27 108.69(16). Selected bond parameters [Å, °] of [**5 b**]^+^: As1‐C10 1.950(1), As1‐C21 1.956(1), As1‐C60 1.982(1), C20‐C21 1.432(2), C20‐C25 1.436(2), C21‐C22 1.362(2), C22‐C23 1.499(2), C24‐C23 1.499(2), C25‐C24 1.376(2), C10‐As1‐C21 84.64(5), C10‐As1‐C60 103.21(5), C21‐As1‐C60 104.80(5), C27‐C23‐C28 109.12(11). Selected bond parameters [Å, °] of [**8 a**]^+^: P1‐C1 1.7962(9), P1‐C10 1.7997(8), P1‐C21 1.7825(8), P1‐C60 1.8269(8), C21‐P1‐C1 110.38(4), C21‐P1‐C10 93.86(4), C1‐P1‐C10 109.84(4), C21‐P1‐C60 114.40(4), C1‐P1‐C60 114.96(4), C10‐P1‐C60, 111.45(4). Selected bond parameters [Å, °] of **9 a**: C10‐P1 1.836(2), C21‐P1 1.824(2), C60‐P1 1.865(2), C20‐C21 1.352(3), C21‐C22 1.459(3), C22‐C26 1.340(3), C22‐C23 1.469(3), C23‐C24 1.348(3), C24‐C25 1.518(3), C20‐C25 1.520(3) C21‐P1‐C10 89.78(9), C21‐P1‐C60 105.15(8), C10‐P1‐C60 111.59(8), C29‐C25‐C30 109.17(17). Selected bond parameters [Å, °] of **9 b**: As1‐C10 1.970(1), As1‐C21 1.946(2), As1‐C60 2.004(1), C21‐C20 1.356(2), C21‐C22 1.465(2), C22‐C26 1.360(2), C22‐C23 1.460(2), C23‐C24 1.352(3), C25‐C24 1.518(3), C20‐C25 1.522(2), C21‐As1‐C60 103.53(6), C21‐As1‐C10 85.58(6), C10‐As1‐C60 106.33(6), C29‐C25‐C30 110.6(1).

In dichloromethane solution the mixture of [**5 a**]^+^ and [**7 a**]^+^ underwent a slow reaction in which a methyl group migrated to phosphorus, over the course of ca. one month, yielding [**8 a**]^+^ as the major product.^16^ Such a reaction did not take place for [**5 b**]^+^ and [**7 b**]^+^ even after six months. The methyl‐phospholium ion [**8 a**]^+^ gave a ^31^P NMR resonance signal at *δ*=21.7 ppm. Inspection of its molecular structure reveals that upon methylation of phosphorus, the C_6_Me_4_ ring rearomatized. The coordination geometry around the phosphorus atom is disordered tetrahedral with bond lengths and angle values that are remarkably close to those of the simplest congener 2,2′‐biphenylenemethylphenylphosphonium iodide despite the steric bulk of the substituents in [**8 a**]^+^.^17^ With tetrahydrofuran, mixtures of [**5 a**]^+^ and [**7 a**]^+^, as well as [**5 b**]^+^ and [**7 b**]^+^, reacted within minutes to afford **9 a** and **9 b**, respectively, as the major species, as a result of a methyl group deprotonation in *trans*‐position of the dimethyl group.^18^ This transformation took place in a seemingly complex equilibrium; the reversal of the reaction by evaporation of THF gave, aside from [**5 a**]^+^ and [**7 a**]^+^, as well as [**5 b**]^+^ and [**7 b**]^+^, a complex mixture of by‐products. In solution, **9 a** is characterized by a ^31^P NMR chemical shift ([D_8_]THF) of *δ*=−24.2 ppm. The molecular structure of neutral species **9 a** (and similarly that of **9 b**) shows that the dearomatized C20–C25 ring contains two parallel double bonds (C23−C24 1.348(3) and C21−C20 1.352(3) Å) and four single bonds with C−C distances ranging between 1.459(3) (C21−C22) and 1.520(3) Å (C20−C25). The C22−C26 distance (**9 a**: 1.340(3) Å; **9 b**: C22−C26 1.360(2) Å) is consistent with a double bond character.

From the very different structures of the targeted pnictogenium ions [**2 a**]^+^ and [**2 b**]^+^ and the actually isolated 9‐pnictogena‐fluorenium ions [**5 a**]^+^ and [**5 b**]^+^ it becomes obvious that the mechanism of the rearrangement involved unobserved cationic intermediates. In an effort to shed some light on the possible mechanism of the rearrangement, DFT calculations were carried out at the B3PW91/6‐311+G(d) level of theory, whereby the relative energies of the isolated arenium ions [**5 a**]^+^ and [**5 b**]^+^ were arbitrarily set to 0 kJ mol^−1^ (Figure 2). The energetically least favorable cation is the initially formed phosphenium ion [**2 a**]^+^, which is 90.9 kJ mol^−1^ higher in energy. Notably, all attempts to geometrically optimize the arsenium ion [**2 b**]^+^ failed and gave the product of the electrophilic attack at the *ortho*‐position, namely, the arenium ion [**3 b**]^+^, which is 3.7 kJ mol^−1^ more stable than the isolated [**5 b**]^+^. The related arenium ion [**3 a**]^+^ is 82.4 kJ mol^−1^ more stable than the phosphenium ion [**2 a**]^+^, but 8.5 kJ mol^−1^ less stable than reference [**5 a**]^+^. From the arenium ions [**3 a**]^+^ and [**3 b**]^+^, formal 1,2‐, 1,3‐, 1,4‐, and 1,5‐methyl shifts give rise to the conceivable arenium ions [**4 a**]^+^ and [**4 b**]^+^ (not observed), [**5 a**]^+^ and [**5 b**]^+^ (observed), [**6 a**]^+^ and [**6 b**]^+^ (not observed), as well as [**7 a**]^+^ and [**7 b**]^+^ (observed), whereby the relative energies correctly reflect the experimental observations. Mechanistically, the circumambulation of the methyl groups might involve intermediate bicycle[3.1.0]hexenyl cations as suggested by Childs and Winstein for the parent heptamethylbenzenium ions [C_6_Me_7_]^+^.^19^ This might also explain why the global energetic minimum, the phospholium ion [**8 a**]^+^, which is 57.1 kJ mol^−1^ more stable than the reference [**5 a**]^+^, is not immediately formed from [**3 a**]^+^, despite the fact that the volatile methyl group is already in close proximity to the phosphorus atom. In the same context it is surprising that the related arsonium ion [**8 b**]^+^ is not experimentally observed, although the energy gain relative to reference [**5 b**]^+^ would be 106.5 kJ mol^−1^. In light of these energy values, the observed slow transfer of the methyl group to the phosphorus might occur by an intermolecular transfer mechanism, perhaps involving traces of aluminum species as transfer reagents. Overall, the rearrangement of the phosphenium ion [**2 a**]^+^ to the methyl‐phospholium ion [**8 a**]^+^ is energetically favored by 248.0 kJ mol^−1^. This might be explained by the fact that the permethylphenyl group is electron‐richer than the mesityl group, which makes it more susceptible to an electrophilic attack. Therefore, this value even exceeds the energy gain (209.6 kJ mol^−1^) associated with the rearrangement of the previously studied [(2,6‐Mes_2_C_6_H_3_)_2_P]^+^ into the related protonated 9‐phospha‐fluorene.^11^

**Figure 2:**
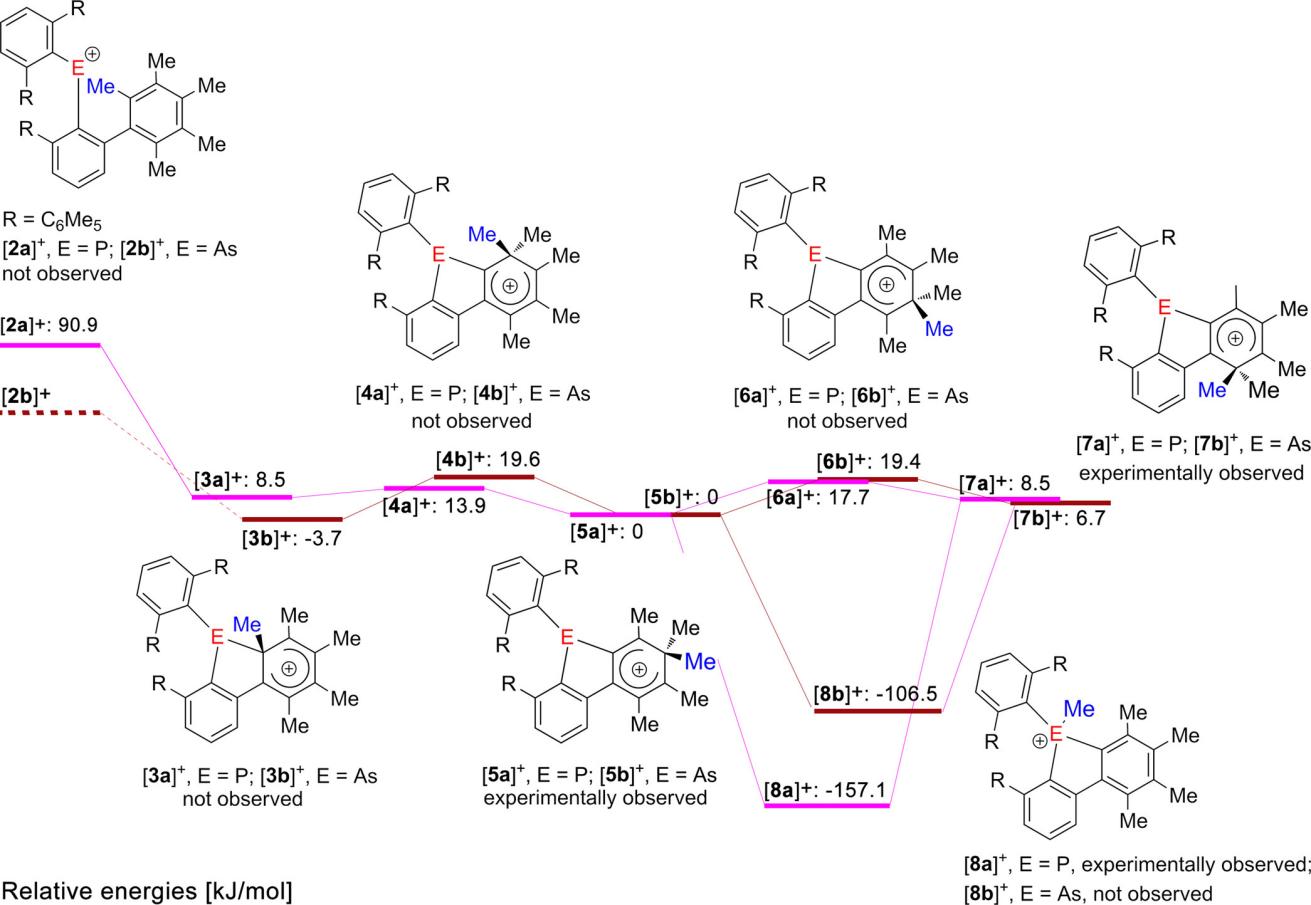
Suggested mechanism of the rearrangement, including relative energies of the pnictogenium ions [**2 a**]^+^, the 9‐pnictogena‐fluorenium ions [**5 a**]^+^ and [**5 b**]^+^ as well as [**7 a**]^+^ and [**7 b**]^+^ and the phospholium ion [ **8 a**]^+^. Attempts to optimize [**2 b**]^+^ always resulted in the conversion into [**3 b**]^+^.

In summary, attempts to prepare the kinetically stabilized phosphenium and arsenium ions [(2,6‐{Me_5_C_6_}_2_C_6_H_3_)_2_E]^+^ (**2 a**, E=P; **2 b**, E=As) lead to the isolation of two‐isomer mixtures of phospha‐fluorenium ions [**5 a**]^+^ and [**7 a**]^+^ (ca. 0.65:0.35 molar ratio) and arsena‐fluorenium ions [**5 b**]^+^ and [**7 b**]^+^ (ca. 0.70:0.30 molar ratio), respectively. Compared to the methyl‐phospholium ion [**8 a**]^+^, the arenium ions [**5 a**]^+^ and [**7 a**]^+^ are high‐energy species; their conversion is slow enough (ca. one month) on the laboratory time scale, which enabled their full characterization. Although thermodynamically also favored, the conversion of the arsena‐fluorenium ions [**5 b**]^+^ and [**7 b**]^+^ into the elusive methyl‐arsolium ion [**8 b**]^+^ is not observed.

## Conflict of interest

The authors declare no conflict of interest.

## Supporting information

As a service to our authors and readers, this journal provides supporting information supplied by the authors. Such materials are peer reviewed and may be re‐organized for online delivery, but are not copy‐edited or typeset. Technical support issues arising from supporting information (other than missing files) should be addressed to the authors.

Supplementary

## References

[1] D. Lenoir , Angew. Chem. Int. Ed. 2003, 42, 854–857; 10.1002/anie.200390231 12596165

[2] G. W. Wheland , J. Am. Chem. Soc. 1942, 64, 900–908.

[3] P. Pfeiffer , R. Wizinger , Liebigs Ann. Chem. 1928, 461, 132–154.

[4a] C. A. Reed , K.-C. Kim , E. S. Stoyanov , D. Stasko , F. S. Tham , L. J. Mueller , P. D. W. Boyd , J. Am. Chem. Soc. 2003, 125, 1796–1804; 12580605 10.1021/ja027336o

[4b] F. Scholz , D. Himmel , L. Eisele , W. Unkrig , I. Krossing , Angew. Chem. Int. Ed. 2014, 53, 1689–1692; 10.1002/anie.201308120 24453134

[5] N. C. Baenziger , A. D. Nelson , J. Am. Chem. Soc. 1968, 90, 6602–6607.

[6] R. Rathore , J. Hecht , J. K. Kochi , J. Am. Chem. Soc. 1998, 120, 13278–13279.

[7] A. V. Vasilyev , S. V. Lindeman , J. K. Kochi , New J. Chem. 2002, 26, 582–592.

[8a] J. B. Lambert , S. Zhang , C. Stern , J. Huffman , Science 1993, 260, 1917–1918; 17836721 10.1126/science.260.5116.1917

[8b] M. F. Ibad , P. Langer , A. Schulz , A. Villinger , J. Am. Chem. Soc. 2011, 133, 21016–21027. 22085216 10.1021/ja209693a

[9a] A. H. Cowley , R. A. Kemp , Chem. Rev. 1985, 85, 367–382;

[9b] D. Gudat , Coord. Chem. Rev. 1997, 163, 71–106;

[9c] N. Burford , P. J. Ragogna , Dalton Trans. 2002, 4307–4315;

[9d] A. P. M. Robertson , P. A. Gray , N. Burford , Angew. Chem. Int. Ed. 2014, 53, 6050–6069; 10.1002/anie.201307658 24861365

[9e] M. Olaru , A. Mischin , L. A. Malaspina , S. Mebs , J. Beckmann , Angew. Chem. Int. Ed. 2020, 59, 1581–1584; 10.1002/anie.201913081 PMC7003730 31751492

[10] M. Olaru , D. Duvinage , S. Mebs , J. Beckmann , Angew. Chem. Int. Ed. 2018, 57, 10080–10084; 10.1002/anie.201803160 29644767

[11] M. Olaru , D. Duvinage , E. Lork , S. Mebs , J. Beckmann , Chem. Eur. J. 2019, 25, 14758–14761. 31404472 10.1002/chem.201902520 PMC6900177

[12] S. Hino , M. M. Olmstead , J. C. Fettinger , P. P. Power , J. Organomet. Chem. 2005, 690, 1638–1644.

[13] M. Olaru , A. Schröder , L. Albers , D. Duvinage , S. Mebs , J. Beckmann , Chem. Eur. J. 2019, 25, 9861–9865. 31095811 10.1002/chem.201902221

[14] Deposition numbers 1993654 (**1a**), 1993655 [**5a**]^+^, 1993656 [**5b**]^+^, 1993657 [**8a**]^+^, 1993658 (**9a**), and 1993659 (**9b**) contain the supplementary crystallographic data for this paper. These data are provided free of charge by the joint Cambridge Crystallographic Data Centre and Fachinformationszentrum Karlsruhe Access Structures service

[15] This observation is in contrast to the heptamethylbenzenium ion [C_6_Me_7_]^+^, which undergoes a methyl shift making all seven methyl groups equivalent at sufficiently high temperatures. B. H. Meier , R. R. Ernst , J. Am. Chem. Soc. 1979, 101, 6441–6442.

[16] The reaction appeared to be faster and cleaner when the solution was exposed to sunlight. A control reaction that was ran in parallel, in complete darkness, was significantly slower (see the Supporting Information).

[17] P. Adkine , T. Cantat , E. Deschamps , L. Ricard , N. Mézailles , P. Le Floch , M. Geoffroy , Phys. Chem. Chem. Phys. 2006, 8, 862–868. 16482328 10.1039/b513736p

[18] The same regioselectivity was observed for the deprotonation of heptamethylbenzenium ion and the protonation of 4-methylene-1,1,2,3,5,6-hexamethylcyclohexadiene-2,5;

[18a] W. von Doering , M. Saunders , H. G. Boyton , H. W. Earhart , E. F. Wadley , W. R. Edwards , G. Laber , Tetrahedron 1958, 4, 178–185;

[18b] H. Hart , P. M. Collins , A. J. Waring , J. Am. Chem. Soc. 1966, 88, 1005–1013;

[18c] M. Attina , F. Cacece , G. de Petris , S. Fornarini , P. Giacomello , J. Am. Chem. Soc. 1985, 107, 2297–2302.

[19] R. F. Childs , S. Winstein , J. Am. Chem. Soc. 1974, 96, 6409–6417.

