# Supplementary material for: Intramolecular Reaction of Transient Phosphenium and Arsenium Ions Giving Rise to Isolable 9‐Phospha‐ and 9‐Arsena‐Fluorenium Ions
Source: Angew Chem Int Ed Engl. 2020 Jul 6;59(34):14414–7. doi: 10.1002/anie.202006728 (PMC7496934; doi:10.1002/anie.202006728)
Supplement: Supplementary file 1 — Supplementary [file ANIE-59-14414-s001.pdf]

Angewandte Chemie
Eine Zeitschrift der Gesellschaft Deutscher Chemiker

Supporting Information

Intramolecular Reaction of Transient Phosphenium and Arsenium Ions Giving Rise to Isolable 9-Phospha- and 9-Arsena-Fluorenium Ions

*Marian Olaru, Daniel Duvinage, Yannik Naß, Lorraine A. Malaspina, Stefan Mebs, and Jens Beckmann**

anie_202006728_sm_miscellaneous_information.pdf

Table of Contents

Experimental procedures 2

General information 2

Synthesis and characterization of **1a** 3

Synthesis and characterization of **1b** 8

Synthesis and characterization of [**5a**][$AlCl_4$] (and [**7a**][$AlCl_4$]) 12

Synthesis and characterization of [**5b**][$AlCl_4$] (and [**7b**][$AlCl_4$]) 28

Synthesis and characterization of [**8a**][$AlCl_4$] 39

Synthesis and characterization of **9a** 52

Synthesis and characterization of **9b** 64

X-Ray diffraction studies 73

Computational data 78

References 79

Experimental procedures

General information

Unless otherwise stated, reactions and manipulations were performed under inert atmosphere (argon) using anhydrous solvents. Reagents used in this work including PCl_3 $AsCl_3$, ZnF_2, $AlCl_3$ and $EtAlCl_2$ (0.9 M in Heptane) were obtained commercially and were used as received. CsF was dried at 140 °C under reduced pressure. The reagents 2,6-$(Me_5C_6)_2C_6H_3I$ and 2,6-$(Me_5C_6)_2C_6H_3Li$ were prepared following the published procedures.[S1] Anhydrous dichloromethane, hexane, tetrahydrofuran and toluene were collected from an SPS800 mBraun solvent purification system and stored over 4 Å molecular sieves. 1,2-Difluorobenzene was degassed and dried under argon over 4 Å molecular sieves. Et_2O was dried by refluxing it over Na/benzophenone under argon atmosphere. Deuterated solvents were degassed and dried over 4 Å molecular sieves under argon.

Unless otherwise noted, NMR spectra were recorded at room temperature on a Bruker Avance 360 and Avance 600 MHz spectrometers. ^{1}H, $^{13}C\{^{1}H\}$, $^{11}B\{^{1}H\}$, $^{31}P\{1H\}$ and ^{19}F NMR spectra are reported on the δ scale (ppm) and are referenced against $SiMe_4$, $BF_3{\cdot}Et_2O$ (15% in $CDCl_3$), H_3PO_4 (85% in water) and $CFCl_3$, respectively. ^{1}H and $^{13}C\{^{1}H\}$ chemical shifts are reported relative to the residual peak of the solvent ($CDHCl_2$ 5.32 ppm for CD_2Cl_2) in the ^{1}H NMR spectra, and to the peak of the deuterated solvent (CD_2Cl_2 53.84 ppm) in the $^{13}C\{^{1}H\}$ NMR spectra.[S2] The assignment of the ^{1}H and $^{13}C\{^{1}H\}$ resonance signals was made in accordance with the COSY, HSQC and HMBC spectra. The labelling schemes are attached to the ^{1}H and $^{13}C\{^{1}H\}$ spectra. With the exception of **1a** and **1b**, for all other compounds, the NMR and the crystal structure labelling schemes are identical.

The ESI HRMS spectra were measured on a Bruker Impact II spectrometer. Acetonitrile or dichloromethane/acetonitrile solutions ($c = 1{\cdot}10^{-5}$ mol·L^{-1}) were injected directly into the spectrometer at a flow rate of 3 µL·min^{-1}. Nitrogen was used both as a drying gas and for nebulization with flow rates of approximately 5 L·min^{-1} and a pressure of 5 psi. Pressure in the mass analyzer region was usually about $1{\cdot}10^{-5}$ mbar. Spectra were collected for 1 min and averaged. The nozzle-skimmer voltage was adjusted individually for each measurement.

Synthesis and characterization of 1a

To a suspension of 2,6-(Me_5C_6)$_2$$C_6H_3$Li (3.01 g, 8.00 mmol) in hexane (10 mL) cooled to −80 °C was added a PCl_3 (1.17 g, 8.52 mmol). The reaction mixture was stirred for 20 min at −80 °C then brought to room temperature for 30 minutes after which all volatiles were removed under reduced pressure. CH_2Cl_2 was added and the suspension was filtered under argon to remove LiCl. All volatiles were removed under reduced pressure to obtain 3.44 g of crude 2,6-(Me_5C_6)$_2$$C_6H_3PCl_2$ that was used without further purification in the next step.* To the crude 2,6-(Me_5C_6)$_2$$C_6H_3PCl_2$, CsF (3.32 g, 21.85 mmol) and THF (15 mL) were added. The reaction mixture was stirred for 72 h at 88 °C. All volatiles were removed under reduced pressure and CH_2Cl_2 (80 mL) was added. The suspension was filtered under argon over a pad of dry Celite, and the solvent was evaporated to dryness to obtain 2.40 g of crude 2,6-(Me_5C_6)$_2$$C_6H_3PF_2$ that was used without further purification in the next step.† To the crude 2,6-(Me_5C_6)$_2$$C_6H_3PF_2$ (2.1 g), additional 2,6-(Me_5C_6)$_2$$C_6H_3$Li (2.01 g, 5.34 mmol) and hexane (50 mL) were added. The mixture was stirred for 18 hours at 65 °C until the 2,6-(Me_5C_6)$_2$$C_6H_3PF_2$ was completely consumed. The solvent was removed under reduced pressure and the crude product was purified by column chromatography (silicagel, hexane:CH_2Cl_2 1:0.4 v/v) to obtain **1a** as a white solid (0.63 g, 17%‡). **Mp** 244–246 °C (decomp.). **^{1}H NMR (600 MHz, CD_2Cl_2)**: δ = 7.13 (t, $^3J(^1H–^1H)$ = 8 Hz, 2H, H4), 6.59 (dd, $^3J(^1H–^1H)$ = 8 Hz, 2H, $^4J(^1H–^{31}P)$ = 2 Hz, 4H, H3, H5), 2.27 (s, 6H, H15), 2.08 (s, 6H, H14 or H16), 2.056 (s, 6H, H14 or H16), 1.60 (s, 6H, H13 or H17), 1.56 (s, 6H, H13 or H17) ppm. **$^{13}C\{^1H\}$ NMR (151 MHz, CD_2Cl_2)**: δ = 147.92 (dd, $^2J(^{13}C–^{31}P)$ = 20 Hz, $^3J(^{13}C–^{19}F)$ = 4 Hz, C6, C2), 140.88 (d, $^2J(^{13}C–^{31}P)$ = 3 Hz, C7), 139.89 (dd, $^1J(^{13}C–^{31}P)$ = 52 Hz, $^2J(^{13}C–^{19}F)$ = 12 Hz, C1), 133.39 (s, C10), 132.71 (s, br, C8 or C12), 132.54 (d, $^3J(^{13}C–^{31}P)$ = 2 Hz, C3, C5), 132.02 (s, C9 or C11), 131.96 (s, br, C8 or C12), 131.84 (s, C8 or C11), 128.92 (s, C4), 19.87 (d, $^2J(^{13}C–^{31}P)$ = 7 Hz, C13 or 17), 19.68 (s, C13 or C17), 17.10 (s, C15), 16.91 (s, C14 or C16), 16.80 (s, C14 or C16) ppm. **$^{31}P\{^1H\}$ NMR (146 MHz, CD_2Cl_2)**: δ = 193.75 (d, $^1J(^{31}P–^{19}F)$ = 851 Hz) ppm. **^{19}F NMR (565 MHz, CD_2Cl_2)**: δ = −200.33 (d, $^1J(^{31}P–^{19}F)$ = 849 Hz). **HRMS ESI (m/z)**: $[M+Na]^+$ calculated. for $C_{56}H_{66}PFNa$, 811.47784; found, 811.47769.

*$^{31}P\{^1H\}$ (THF-*d8*) = 162.5 ppm (s).

† $^{31}P\{^1H\}$ (C_6D_6) = 214.0 ppm (t, $^1J(^{19}F–^{31}P)$ = 1164 Hz; ^{19}F (C_6D_6) = −92.92 ppm (d, $^1J(^{19}F–^{31}P)$ = 1164 Hz).

‡ Several attempts to optimize the reaction for a better yield were unsuccessful.

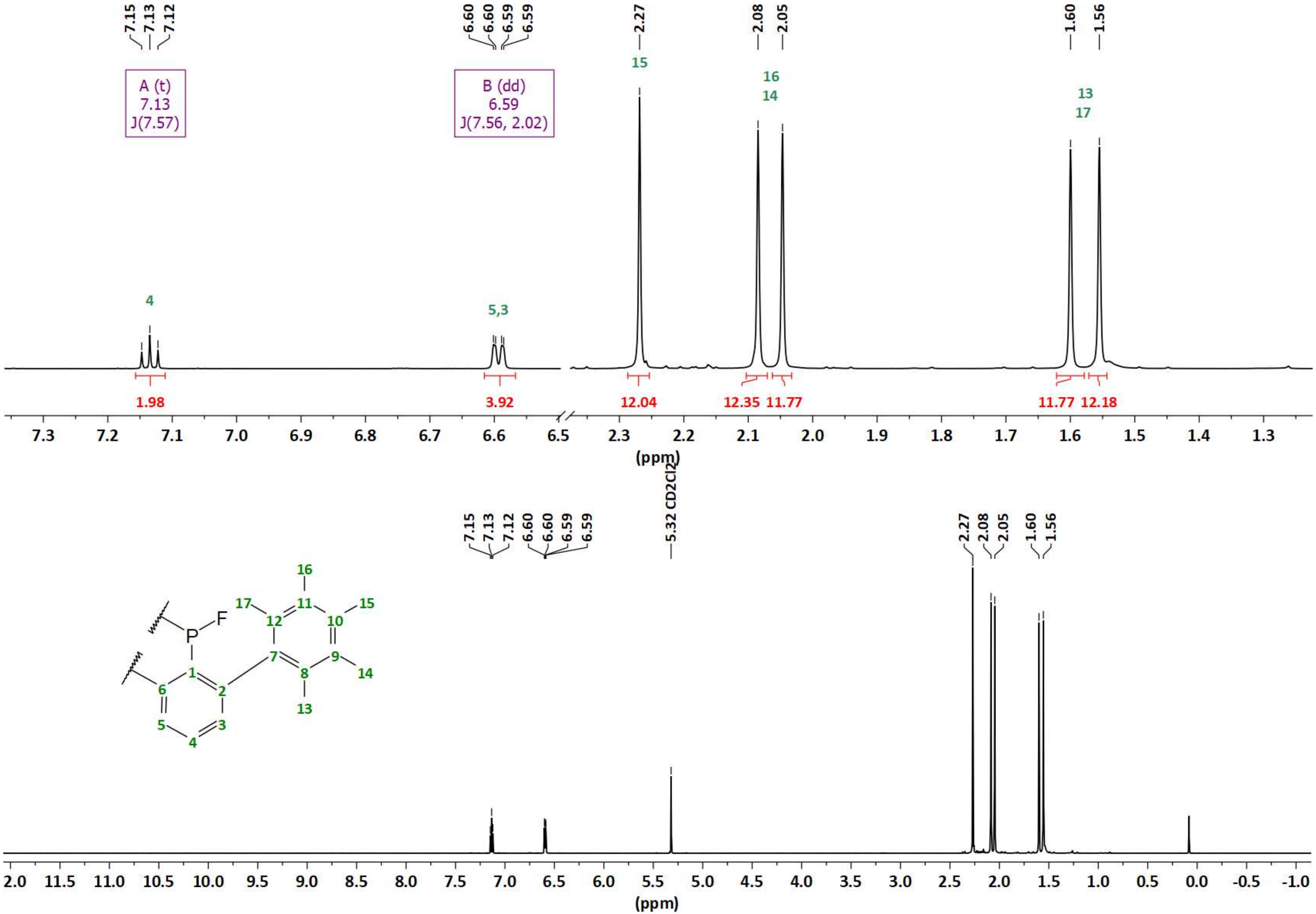

Figure S1. ^{1}H NMR (CD_2Cl_2, 600 MHz) spectrum of **1a**.

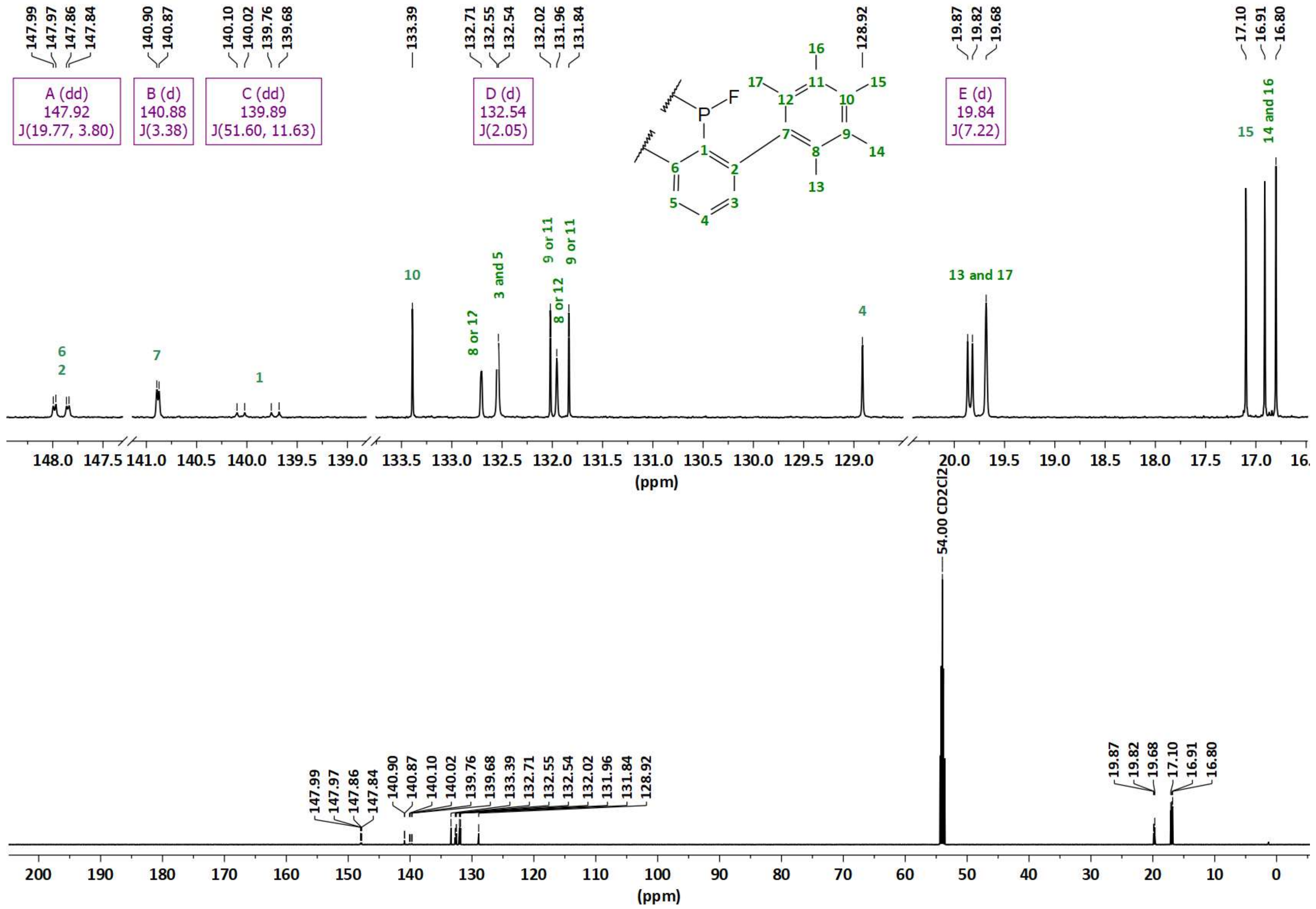

Figure S2. $^{13}C\{^{1}H\}$ NMR (CD_2Cl_2, 151 MHz) spectrum of **1a**.

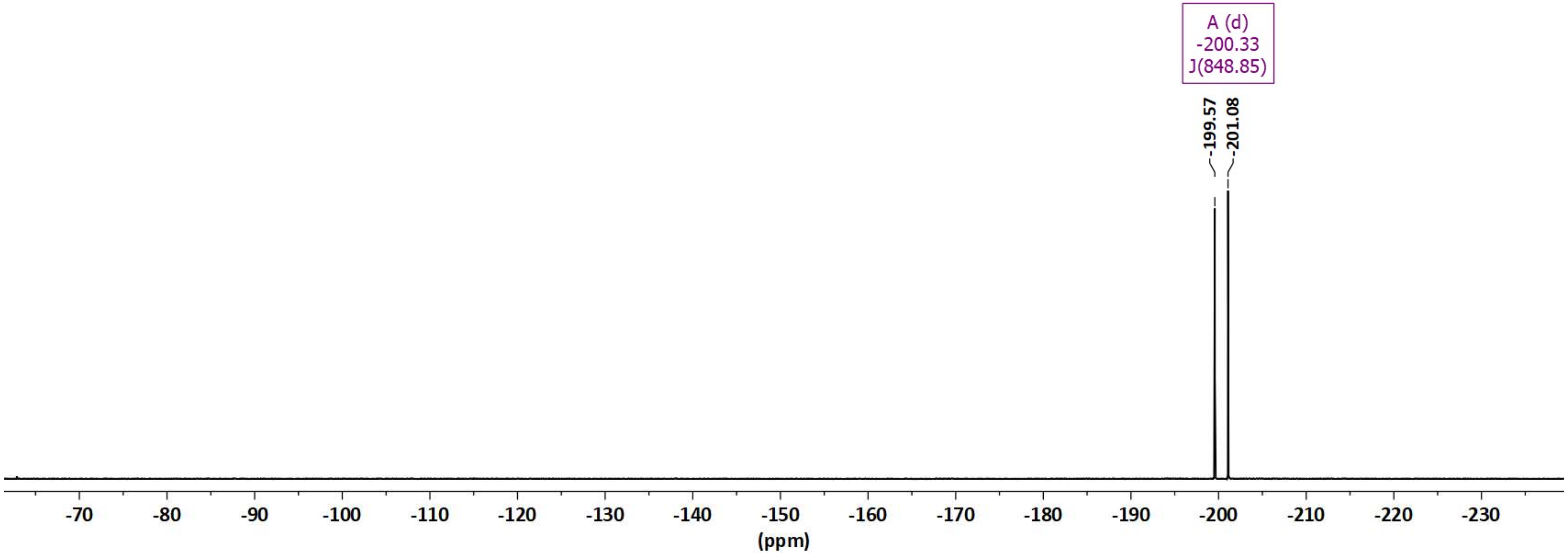

Figure S3. ^{19}F NMR (CD_2Cl_2, 565 MHz) spectrum of **1a**.

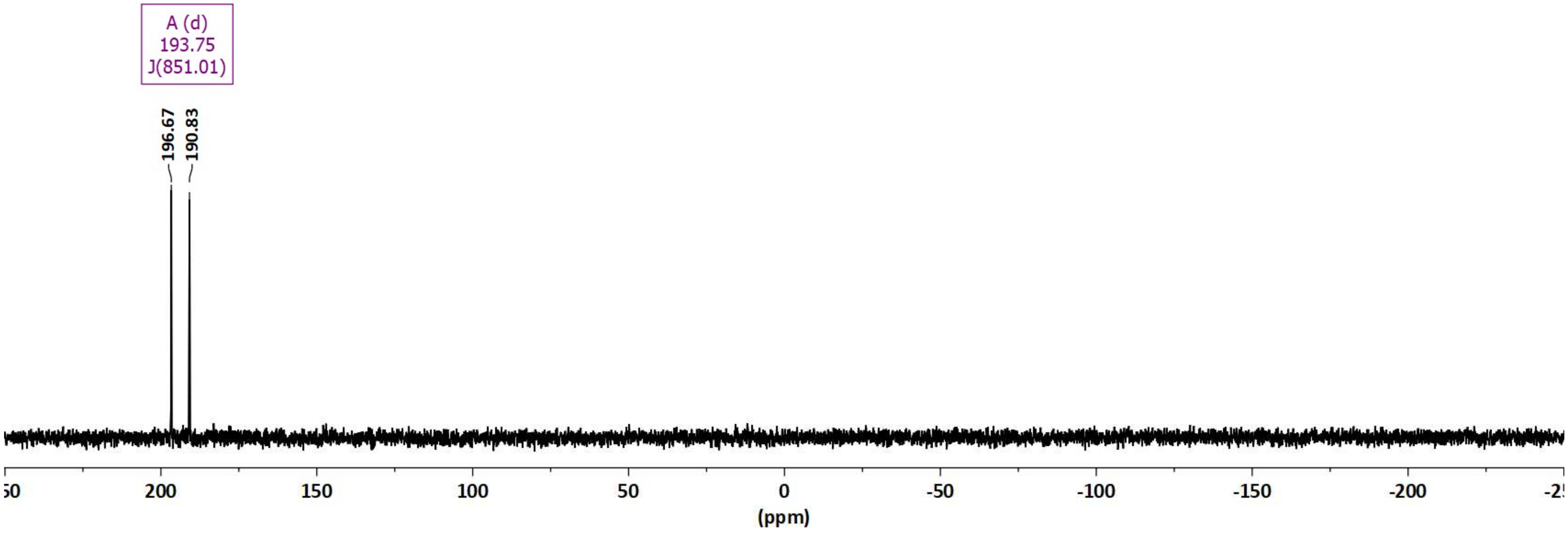

Figure S4. $^{31}P\{^{1}H\}$ NMR (CD_2Cl_2, 146 MHz) spectrum of **1a**.

Synthesis and characterization of 1b

A solution of 2,6-($Me_5C_6)_2C_6H_3Li$ (4.20 g, 11.15 mmol) in Et_2O (50 mL) was added to a solution of $AsCl_3$ (2.10 g, 11.58 mmol) in Et_2O (20 mL) at 0°C over the course of 20 minutes. The reaction mixture was brought to room temperature and stirred for additional 16 hours. The solvent was removed under reduced pressure. To the crude mixture ZnF_2 (5.50 g, 53.19 mmol) and MeCN (120 mL) were added. The reaction mixture was stirred for 120 h at 80 °C. After reaction control by ^{19}F NMR spectroscopy the solvent was removed under vacuum and to the residue CH_2Cl_2 (120 mL) was added. The suspension was filtered through a pad of Celite and the solvent of the solution was removed under reduced pressure. The remaining solid was washed with cold MeCN (3×20 mL) and with cold hexane (3×10 mL). To the crude 2,6-($Me_5C_6)_2C_6H_3AsF_2$ thus obtained, 2,6-($Me_5C_6)_2C_6H_3Li$ (4.20 g, 11.16 mmol) was added and the solids were suspended in hexane. The reaction mixture was stirred for 24 h at room temperature and controlled by ^{19}F NMR spectroscopy. Afterwards the solvent was removed under reduced pressure and to the residual solid CH_2Cl_2 (300 mL) was added. The suspension was worked up aqueous by washing the organic phase with distilled H_2O (3×100 mL). The organic phase was dried over sodium sulphate and the solvent was removed under vacuum to afford a beige solid. This was washed with cold MeCN (3×40 mL) and cold hexane (3×40 mL) to obtain the title compound **1b** as colourless solid (4.29 g, 46%). Crystals suitable for X-ray diffraction were obtained by dissolving **1b** in CH_2Cl_2 and slow diffusion of hexane. **Mp**: 250–255 °C (decomp.). **^{1}H NMR (600 MHz, CD_2Cl_2)**: δ = 7.15 (2H, t, $^3J(^1H–^1H)$ = 7.5 Hz, H4), 6.60 (4H, d, $^2J(^1H–^1H)$ = 7.5 Hz, H3 and H5), 2.28 (12H, s, H15), 2.11 (12H, s, H14 or H16), 2.06 (12H, s, H14 or 16), 1.62 (12H, s, H13 or H17), 1.60 (12H, s, H13 or H17) ppm. **$^{13}C\{^1H\}$ NMR (151 MHz, CD_2Cl_2)**: δ = 148.33 (4C, s, C2 and C6), 145.74. (2C, d, $^3J(^{13}C–^{19}F)$ = 11.8 Hz, C1), 140.59 (4C, s, C7), 133.77 (4C, s, C10), 132.89 (4C, s, C8 or C12), 132.40 (4C, s, C9 or C11), 132.20 (4C, s, C3), 132.03 (4C, s, C9 or C11), 131.96 (4C, s, C8 or C12), 128.96 (2C, s, C4), 19.83 (4C, s, C13 or C17), 19.72 (4C, s, C13 or C17), 17.14 (4C, s, C15), 16.99 (4C, s, C14 or C16), 16.83 (4C, s, C14 or C16) ppm. **^{19}F NMR (565 MHz, CD_2Cl_2)**: δ = −216 (1F, s, As–F) ppm. **HRMS ESI (m/z)**: $[M+Na]^+$ calculated for $C_{56}H_{66}AsFNa$ 855.42567; found 855.42504.

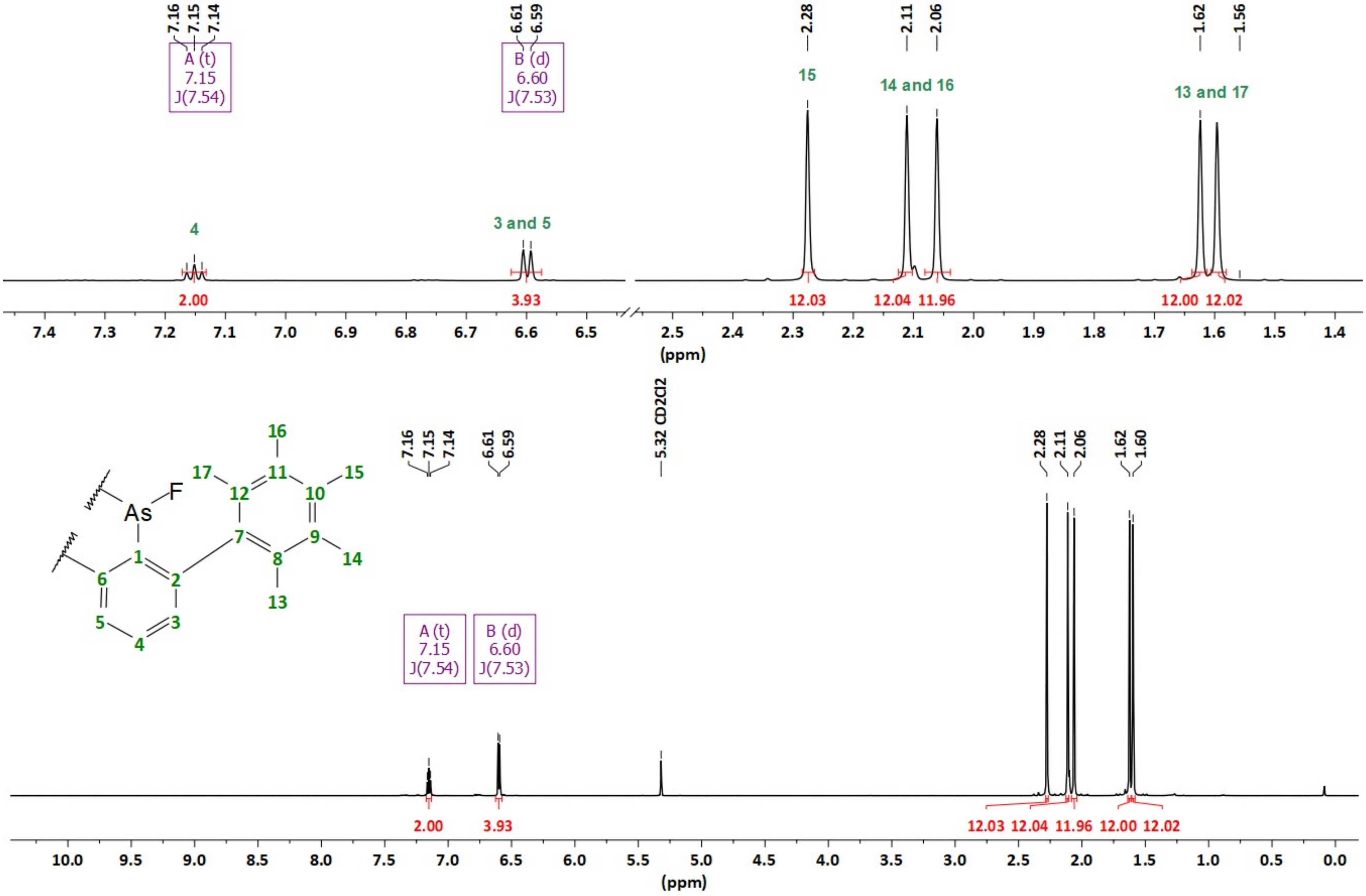

Figure S5. ^{1}H NMR (CD_2Cl_2, 600 MHz) spectrum of **1b**.

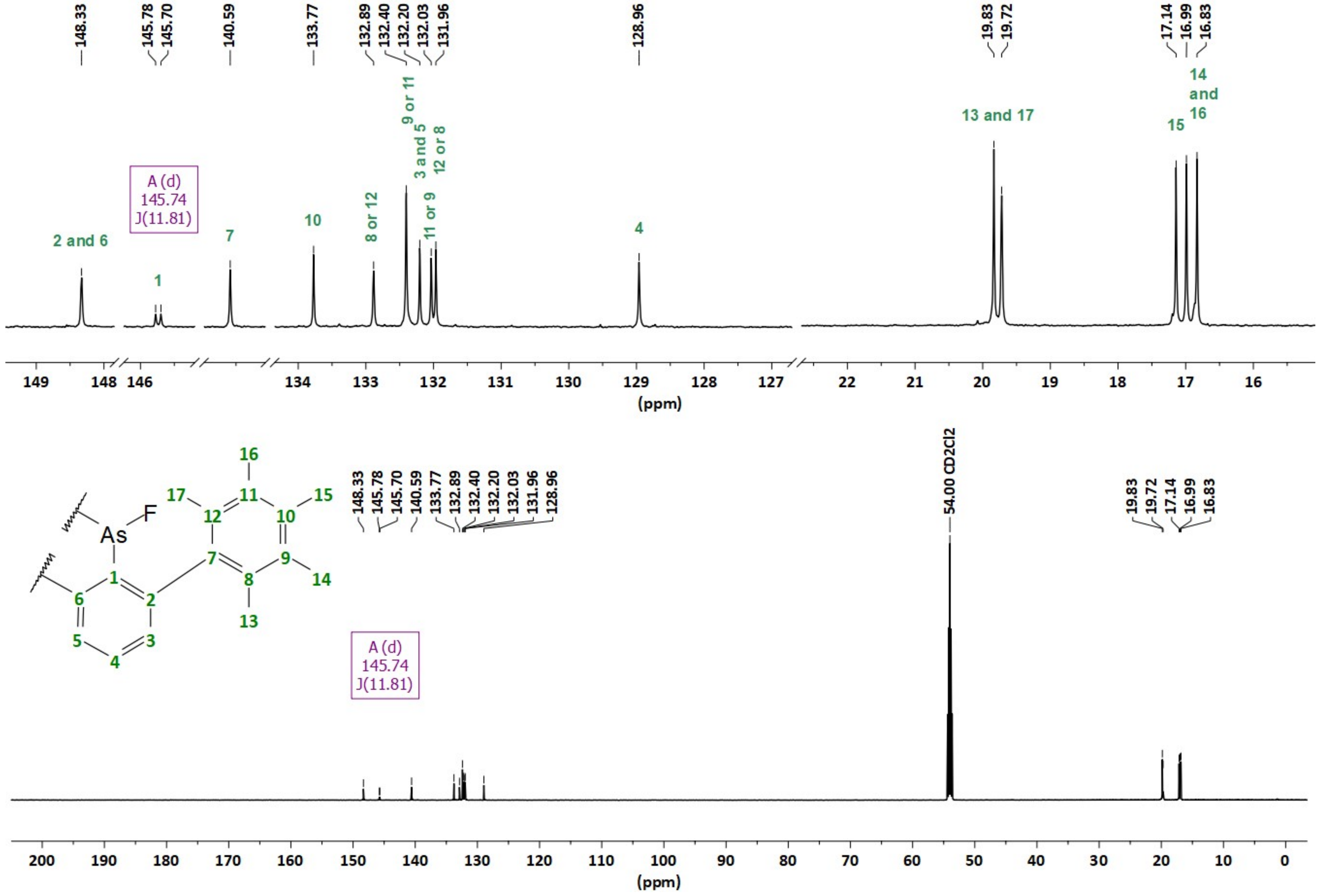

Figure S6. $^{13}C\{^1H\}$ NMR (CD_2Cl_2, 151 MHz) spectrum of **1b**.

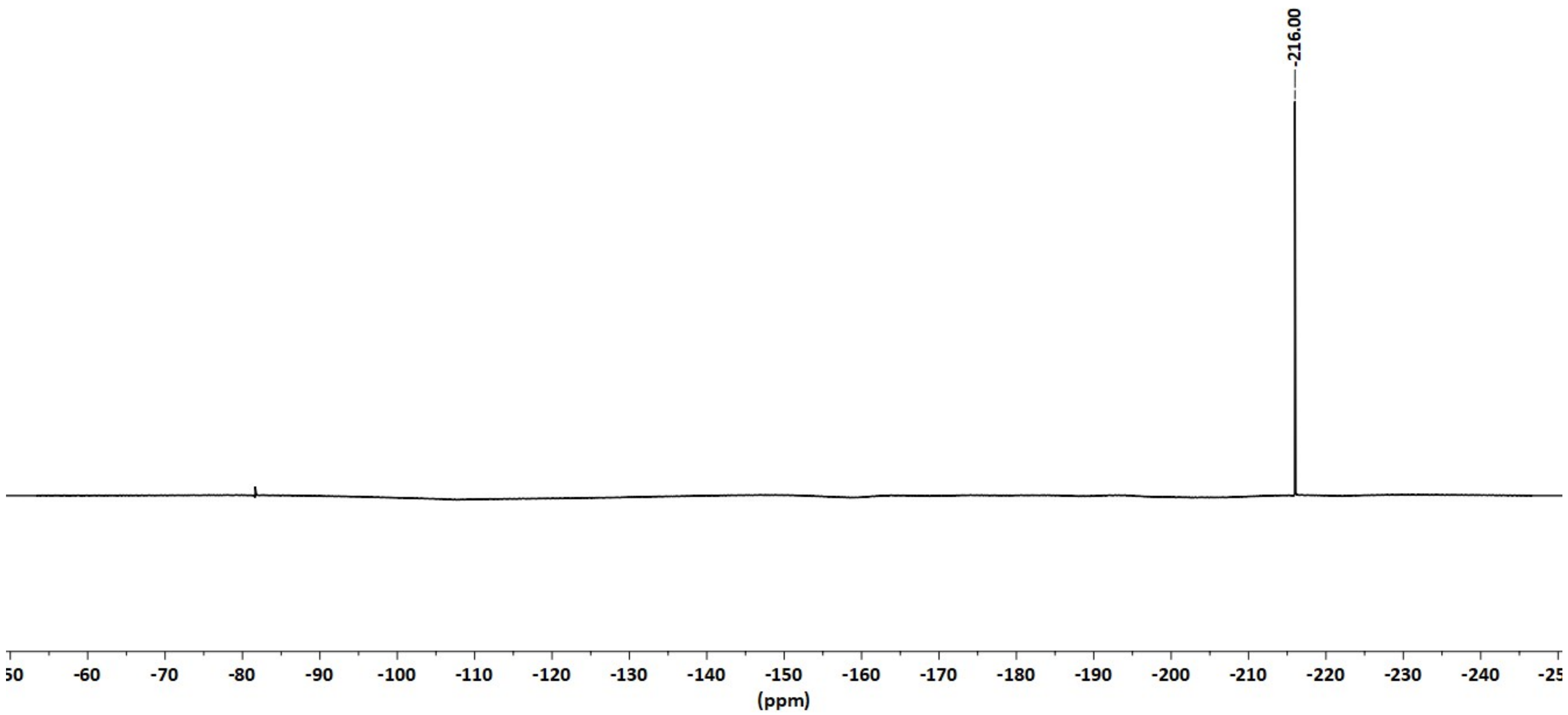

Figure S7. ^{19}F NMR (CD_2Cl_2, 565 MHz) spectrum of **1b**.

Synthesis and characterization of [5a][$AlCl_4$] (and [7a][$AlCl_4$])

To a suspension of **1a** (100 mg, 0.13 mmol) in pentane (3 mL) and CH_2Cl_2 (3 mL) a solution of $EtAlCl_2$ in heptane (0.9 M, 0.7 mL, 0.63 mmol) was added at room temperature. The reaction mixture was stirred at room temperature for 10 minutes. Pentane (20 mL) was layered and the Schlenk tube was placed in a refrigerator at 4 °C for 4–6 h. The crystals were separated from the solution and then dried under reduced pressure to obtain a dark red-brown crystalline solid (110 mg, virtually quantitative yield). The NMR spectra indicated a mixture of two products, [**5a**][$AlCl_4$] (major) and [**7a**][$AlCl_4$] (minor) always in the same molar ratio (*ca.* 1:0.35). From all crystallization experiments only [**5a**][$AlCl_4$] produced crystals suitable for single-crystal X-ray structure determination. The identity of [**7a**][$AlCl_4$] was inferred from NMR spectra.

Major isomer [**5a**]$^+$:

^{1}H NMR (600 MHz, CD_2Cl_2): δ =8.13 (d, $^3J(^1H–^1H)$ = 8 Hz, $^5J(^1H–^{31}P)$ = 1 Hz, 1H, H12), 7.68 (dd, $^3J(^1H–^1H)$ = 8, 7.3 Hz, 1H, H13), 7.44 (td, $^3J(^1H–^1H)$ = 8 Hz, $^5J(^1H–^{31}P)$ = 1 Hz, 1H, H63), 7.39 (ddd, $^3J(^1H–^1H)$ = 8 Hz, $^4J(^1H–^1H)$ = 4 Hz, $^5J(^1H–^{31}P)$ = 1 Hz, 1H, H14), 7.03 (ddd, $^3J(^1H–^1H)$ = 8 Hz, $^4J(^1H–^1H)$ = 5 Hz, $^5J(^1H–^{31}P)$ = 2 Hz, 1H, H64), 6.68 (dd, $^3J(^1H–^1H)$ = 8 Hz, $^5J(^1H–^{31}P)$ = 2 Hz, 1H, H62), 2.52 (d, $^6J(^1H–^{31}P)$ = 3 Hz, 3H, H29), 2.43 (d, $^3J(^1H–^{31}P)$ = 2 Hz, 3H, H26), 2.34 (s, 3H, H48), 2.28 (s, 3H, H78), 2.28 (s, 3H, H30), 2.27 (s, 3H, H97), 2.20 (s, 6H, H98, H47), 2.17 (s, 3H, H49), 2.09 (s, 3H, H79), 2.00 (s, 6H, H46, H99), 1.78 (s, 3H, H77),1.70 (s, br, 6H, H28, H96), 1.54 (s, 3H, H80), 1.25 (s, 3H, H27), 1.20 (s, 3H, H50), 1.16 (s, 3H, H76), 0.91 (s, 3H, H100) ppm. **$^{13}C\{^1H\}$ NMR (151 MHz, CD_2Cl_2)**: δ = 192.43 (d, $^2J(^{13}C–^{31}P)$ = 16 Hz, C22), 188.97 (d, $^4J(^{13}C–^{31}P)$ = 3 Hz, C24), 157.17 (s, C20), 155.58 (d, $^1J(^{13}C–^{31}P)$ = 12 Hz, C10), 154.80 (s, $^2J(^{13}C–^{31}P)$ = 46 Hz, C65), 150.48 (d, $^2J(^{13}C–^{31}P)$ = 22 Hz, C11), 150.00 (d, $^2J(^{13}C–^{31}P)$ = 7 Hz, C61), 146.12 (d, $^2J(^{13}C–^{31}P)$ = 4 Hz, C15), 143.39 (s, $^1J(^{13}C–^{31}P)$ = 11 Hz, C21), 142.42 (d, $^3J(^{13}C–^{31}P)$ = 6 Hz, C14), 139.48 (d, $^3J(^{13}C–^{31}P)$ = 10 Hz, C90), 138.92 (d, $^3J(^{13}C–^{31}P)$ = 2 Hz, C40), 137.75 (s, C70), 136.51 (s, C43), 135.46 (s, C73), 135.01 (s, C93), 134.94 (s, C74), 134.02 (s, C42), 133.81 (d, $^3J(^{13}C–^{31}P)$ = 3 Hz, C25), 133.46 (s, C44), 133.42 (s, C12), 133.24 (s, C72), 132.88 (s, C75), 132.73 (s, C71), 132.58 (s, C92), 132.56 (s, C62, C95), 132.19 (s, C94), 132.13 (s, C91), 132.13 (s, C63), 132.04 (s, C45), 131.90 (s, C41), 131.70 (d, $^3J(^{13}C–^{31}P)$ = 9 Hz, C64), 131.13 (s, C13), 127.01 (d, $^1J(^{13}C–^{31}P)$ = 34 Hz, C60), 54.24 (d, $^3J(^{13}C–^{31}P)$ = 3 Hz, C23), 30.90 (s, C27), 25.31 (d, $^3J(^{13}C–^{31}P)$ = 22 Hz, C26), 24.35 (s, C28), 22.11 (s, br, C29), 21.18 (s, C80), 20.06 (s, C96), 19.95 (s, C46), 19.52 (s, C76), 19.15 (s, C30). 17.98 (d, $^4J(^{13}C–^{31}P)$ = 8 Hz, C50), 17.44 (s, C78) 17.37 (d, $^5J(^{13}C–^{31}P)$ = 5 Hz, C100), 17.25 (s, C48), 17.11 (s, C49), 17.08 (s, C97),

16.92 (s, C77), 16.87 (s, C47, C98), 16.83 (s, C79), 16.29 (s, C99) ppm. **$^{31}P\{^1H\}$ NMR (CD_2Cl_2, 243 MHz)**: δ = −26.93 (s) ppm.

Minor isomer [**7b**]$^+$:

^1H NMR (600 MHz, CD_2Cl_2): δ = 7.96 (d, $^3J(^1H–^1H)$ = 8 Hz, $^5J(^1H–^{31}P)$ = 1 Hz, 1H, H12), 7.74 (dd, $^3J(^1H–^1H)$ = 8, 7.4 Hz, 1H, H13), 7.46 (td, $^3J(^1H–^1H)$ = 8 Hz, $^5J(^1H–^{31}P)$ = 1 Hz, H, H63), 7.31 (ddd, $^3J(^1H–^1H)$ = 7 Hz, $^4J(^1H–^1H)$ = 4 Hz, $^5J(^1H–^{31}P)$ = 1 Hz, 1H, H14), 7.09 (ddd, $^3J(^1H–^1H)$ = 8 Hz, $^4J(^1H–^1H)$ = 6 Hz, $^5J(^1H–^{31}P)$ = 2 Hz, 1H, H64), 6.62 (dd, $^3J(^1H–^1H)$ = 8 Hz, $^5J(^1H–^{31}P)$ = 2 Hz, 1H, H62), 2.74 (d, $^3J(^1H–^{31}P)$ = 1 Hz, 3H, H26), 2.51 (s, br, 3H, H28), 2.34 (s, 3H, H34), 2.29 (s, 3H, H27), 2.26 (s, 3H, H98), 2.21 (s, 3H, H47), 2.20 (s, 3H, H97), 2.15 (s, 3H, H49), 2.10 (s, 3H, H78), 2.01 (s, 3H, H46), 2.00 (s, 3H, H99), 1.93 (s, 3H, H79), 1.84 (s, 3H, H77), 1.79 (s, 3H, H96), 1.49 (s, 3H, H30), 1.43 (s, 3H, H80), 1.29 (s, 3H, H76), 1.26 (s, 3H, H29), 1.15 (s, 3H, H50), 0.94 (s, 3H, H100) ppm. **$^{13}C\{^1H\}$ NMR (151 MHz, CD_2Cl_2)**: δ = 191.61 (d, $^2J(^{13}C–^{31}P)$ = 7 Hz, C20), 189.39 (s, C24), 183.70 (d, $^2J(^{13}C–^{31}P)$ = 18 Hz, C22), 154.90 (d, $^2J(^{13}C–^{31}P)$ = 45 Hz, C65), 151.15 (d, $^2J(^{13}C–^{31}P)$ = 6 Hz, C61), 150.47 (d, $^1J(^{13}C–^{31}P)$ = 4 Hz, C10), 150.37 (s, $^2J(^{13}C–^{31}P)$ = 15 Hz, C11), 145.92 (d, $^1J(^{13}C–^{31}P)$ = 10 Hz, C21), 145.75 (s, $^2J(^{13}C–^{31}P)$ = 3 Hz, C15), 139.94 (d, $^3J(^{13}C–^{31}P)$ = 7 Hz, C14), 139.46 (d, $^3J(^{13}C–^{31}P)$ = 9 Hz, C90), 139.27 (s, C40), 137.43 (s, C70), 136.46 (s, C43), 135.82 (d, $^3J(^{13}C–^{31}P)$ = 5 Hz, C23), 135.46 (s, C73), 135.15 (s, C93), 133.95 (s, C42), 133.50 (s, C44), 133.15 (s, C74), 132.86 (s, C71) 132.70 (d, $^4J(^{13}C–^{31}P)$ = 2 Hz, C95), 132.67 (s, C92), 132.50 (d, $^3J(^{13}C–^{31}P)$ = 2 Hz, C62), 132.35 (s, C75), 132.33 (s, C94), 132.30 (d, $^4J(^{13}C–^{31}P)$ = 2 Hz, C63), 132.20 (s, C45), 131.99 (d, $^3J(^{13}C–^{31}P)$ = 10 Hz, C64), 131.94 (s, C91, C41), 131.53 (s, C72), 131.00 (s, C13), 129.39 (s, C12), 125.45 (d, $^1J(^{13}C–^{31}P)$ = 25 Hz, C60), 52.41 (d, $^3J(^{13}C–^{31}P)$ = 2 Hz, C25) 35.11 (d, $^4J(^{13}C–^{31}P)$ = 3 Hz, C29), 28.69 (d,$^3J(^{13}C–^{31}P)$ = 17 Hz, C26), 25.17 (d, $^4J(^{13}C–^{31}P)$ = 2 Hz, C30), 21.75 (s, C28), 20.61 (s, C80), 20.26 (s, C96), 19.84 (s, C46), 19.65 (s, C76), 17.71 (d, $^5J(^{13}C–^{31}P)$ = 7 Hz, C50), 17.48 (d, $^5J(^{13}C–^{31}P)$ = 4 Hz, C100), 17.25 (s, C48), 17.14 (s, C78), 17.08 (s, C49, C98), 16.87 (s, C97), 16.84 (s, br, C47, C77), 16.52 (s, C79), 16.31 (s, C99), 15.83 (d, $^4J(^{13}C–^{31}P)$ = 2 Hz, C27) ppm. **$^{31}P\{^1H\}$ NMR (243 MHz, CD_2Cl_2)**: δ = −22.15 (s) ppm.

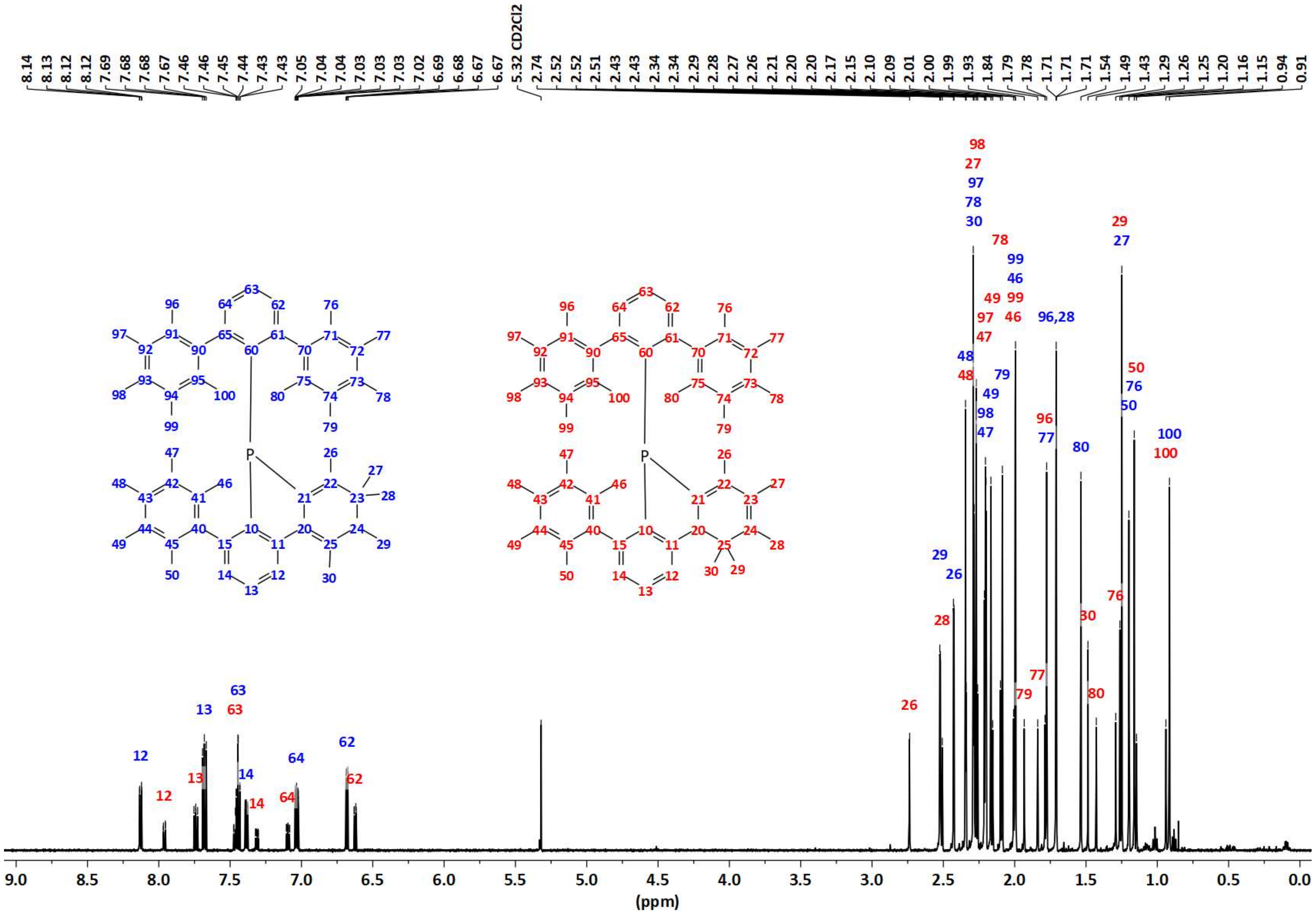

Figure S8. ^{1}H NMR (CD_2Cl_2, 600 MHz) spectrum (full) of [**5a**]$^+$ and [**7a**]$^+$.

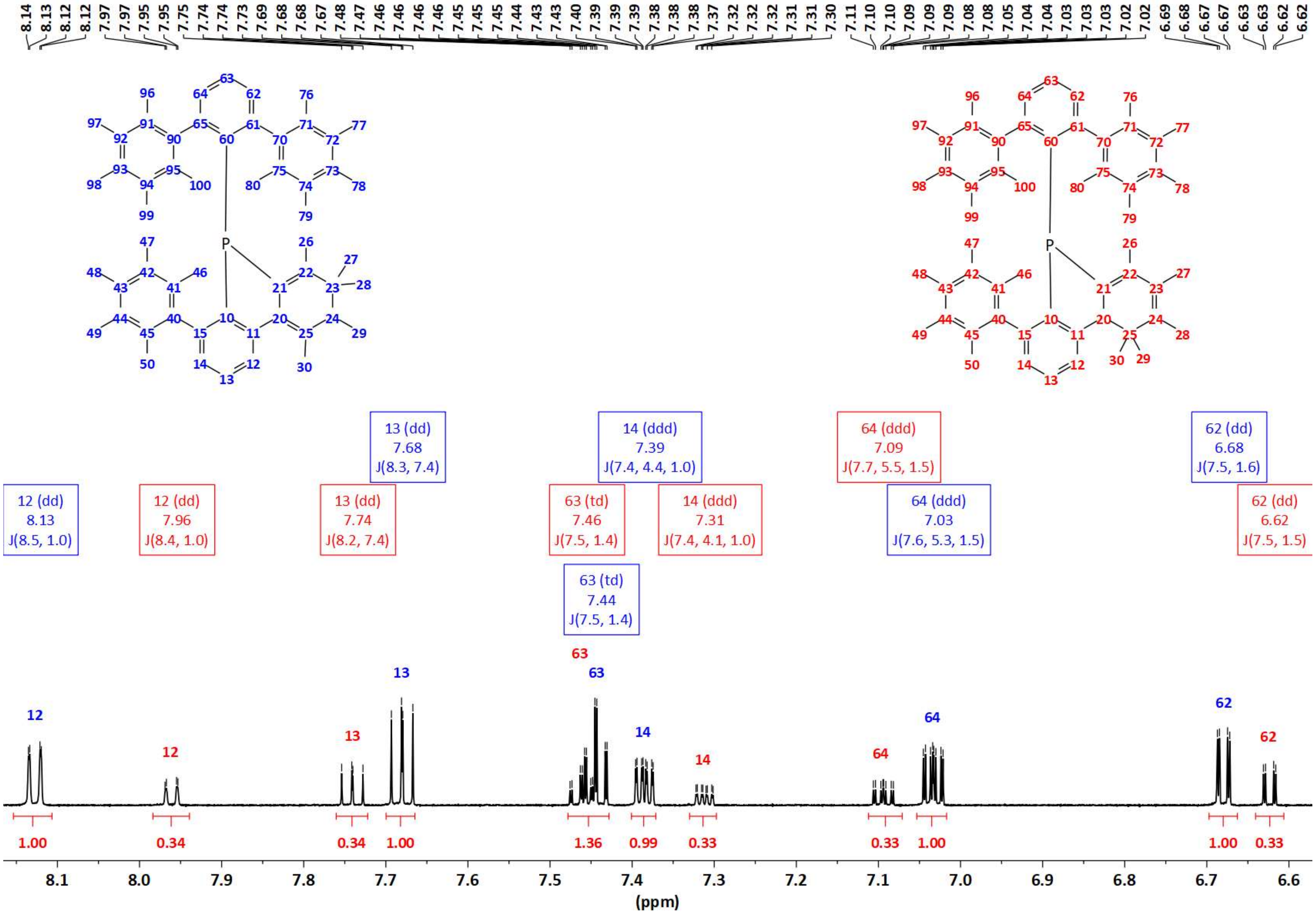

Figure S9. ^{1}H NMR (CD_2Cl_2, 600 MHz) spectrum (detail) of [**5a**]$^{+}$ and [**7a**]$^{+}$.

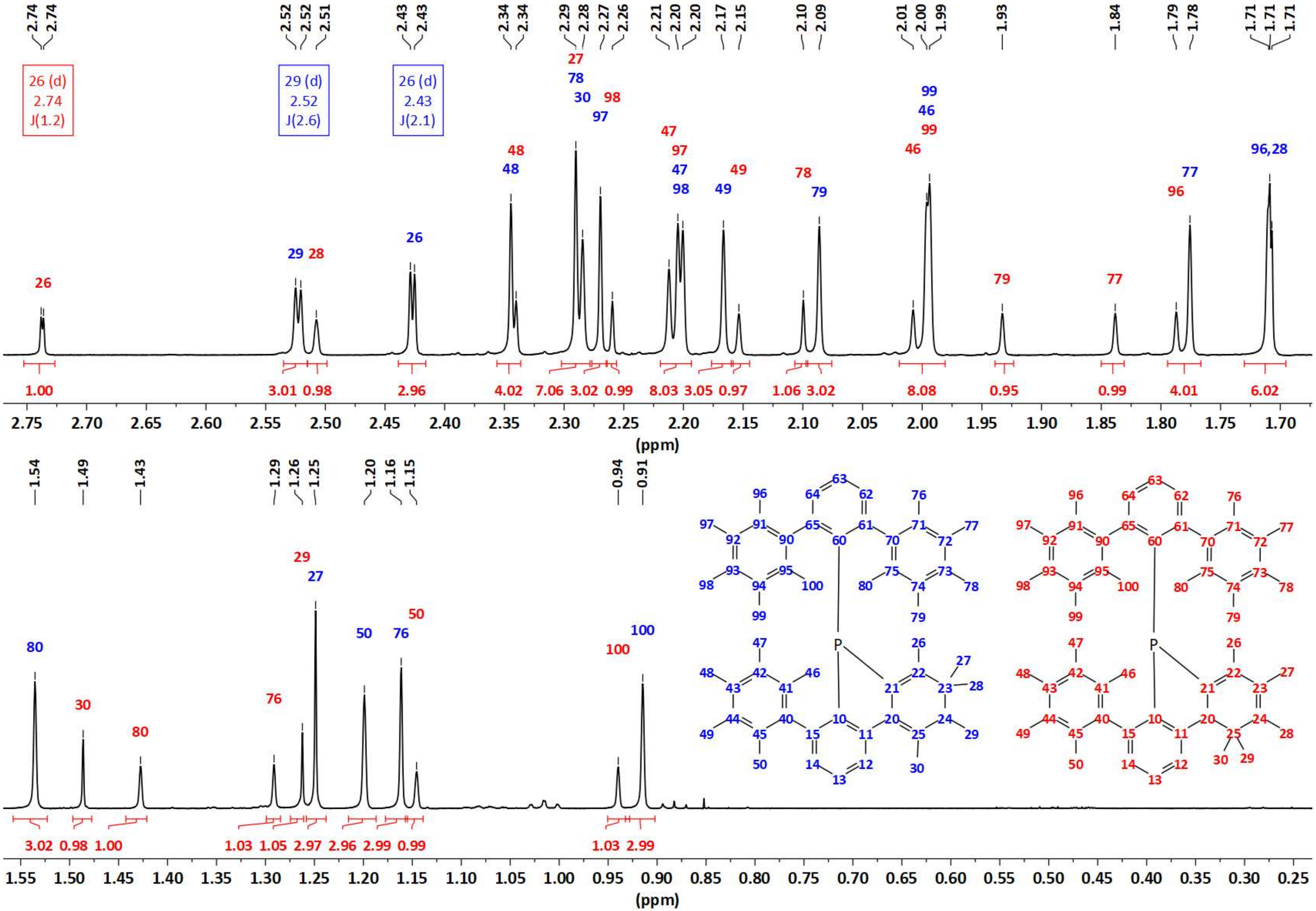

Figure S10. ^{1}H NMR (CD_2Cl_2, 600 MHz) spectrum (detail) of [**5a**]$^{+}$ and [**7a**]$^{+}$.

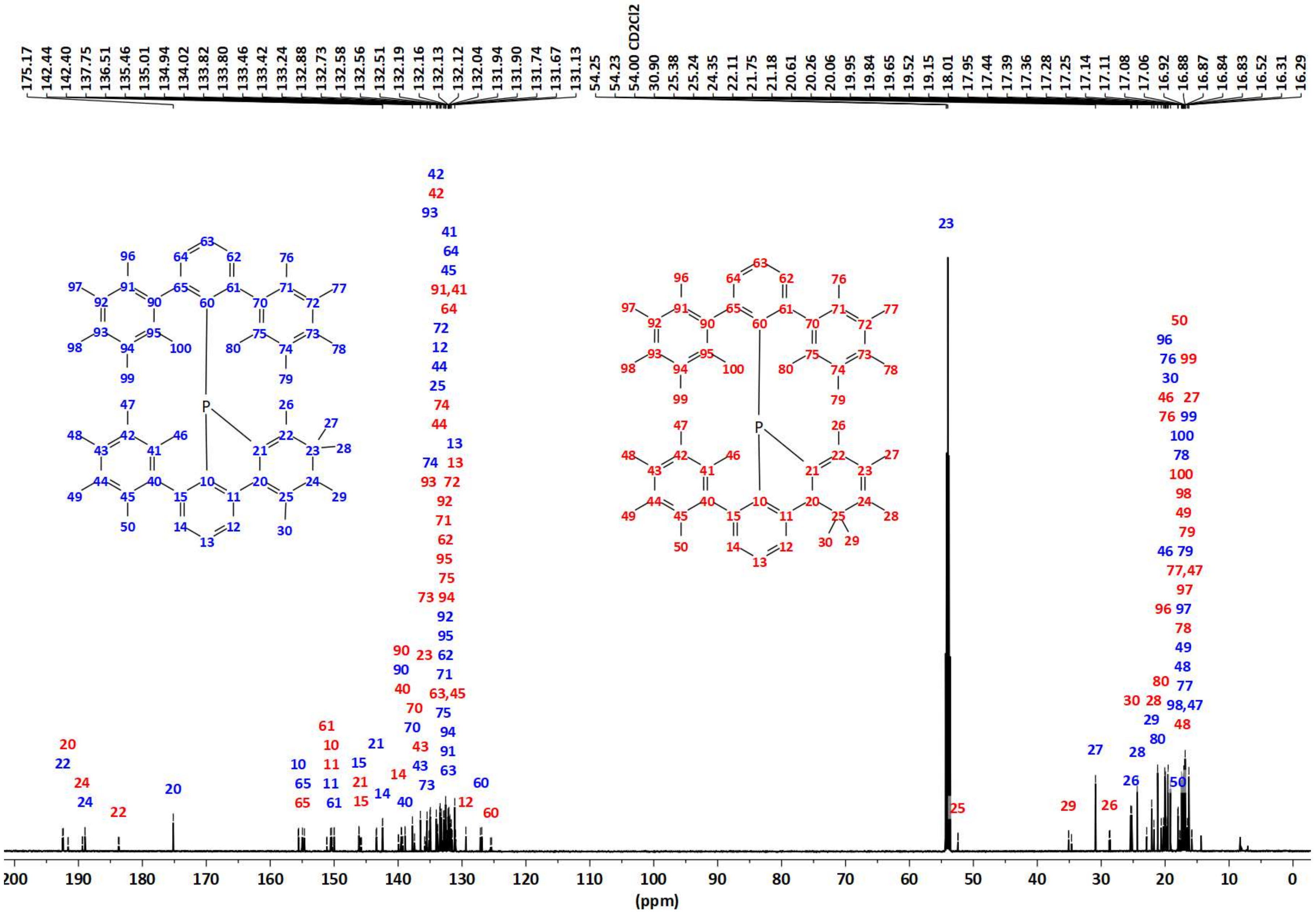

Figure S11. ^{13}C NMR (CD_2Cl_2, 151 MHz) spectrum (full) of [**5a**]$^{+}$ and [**7a**]$^{+}$.

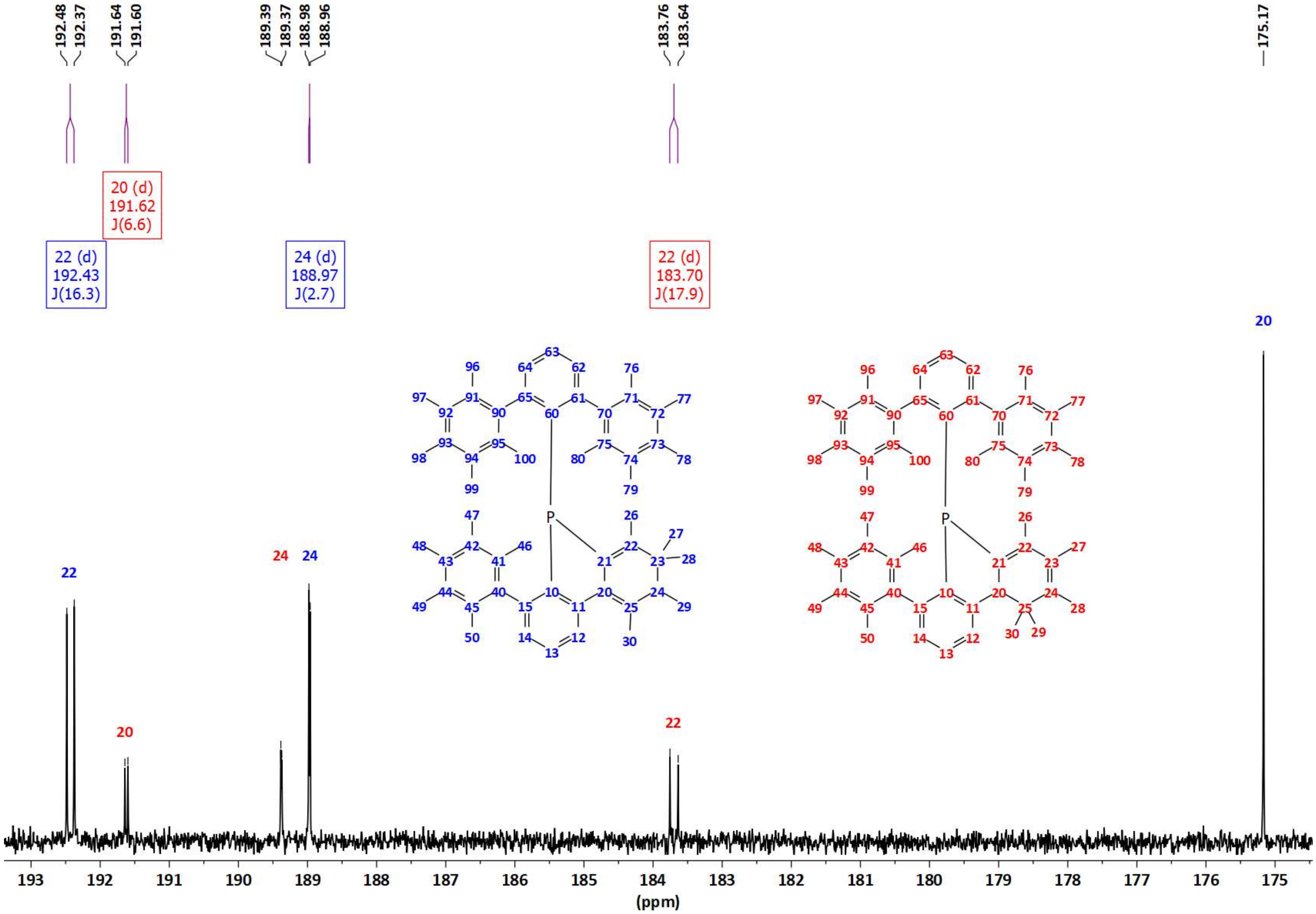

Figure S12. ^{13}C NMR (CD_2Cl_2, 151 MHz) spectrum (detail) of [**5a**]$^+$ and [**7a**]$^+$.

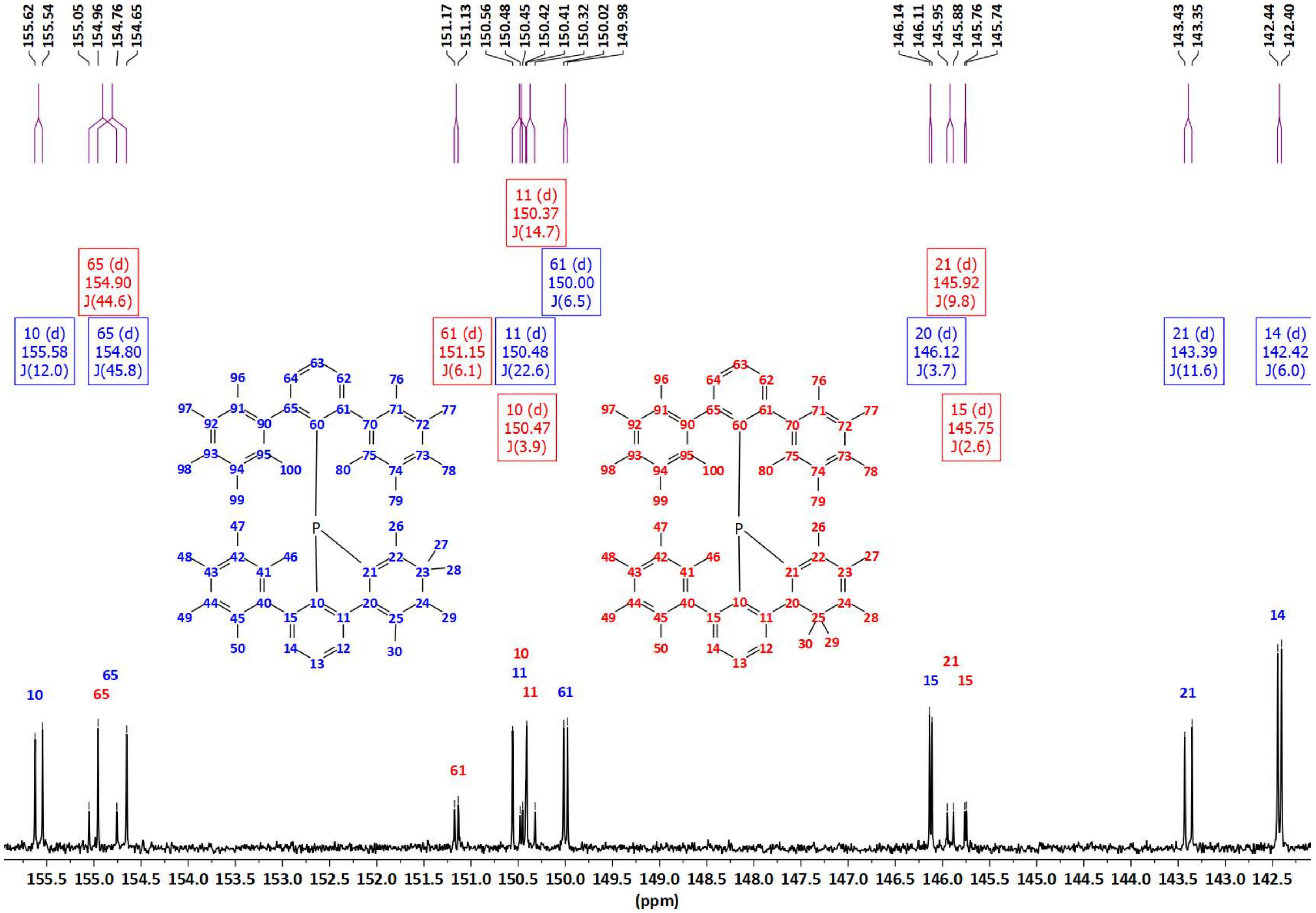

Figure S13. ^{13}C NMR (CD_2Cl_2, 151 MHz) spectrum (detail) of [**5a**]$^+$ and [**7a**]$^+$.

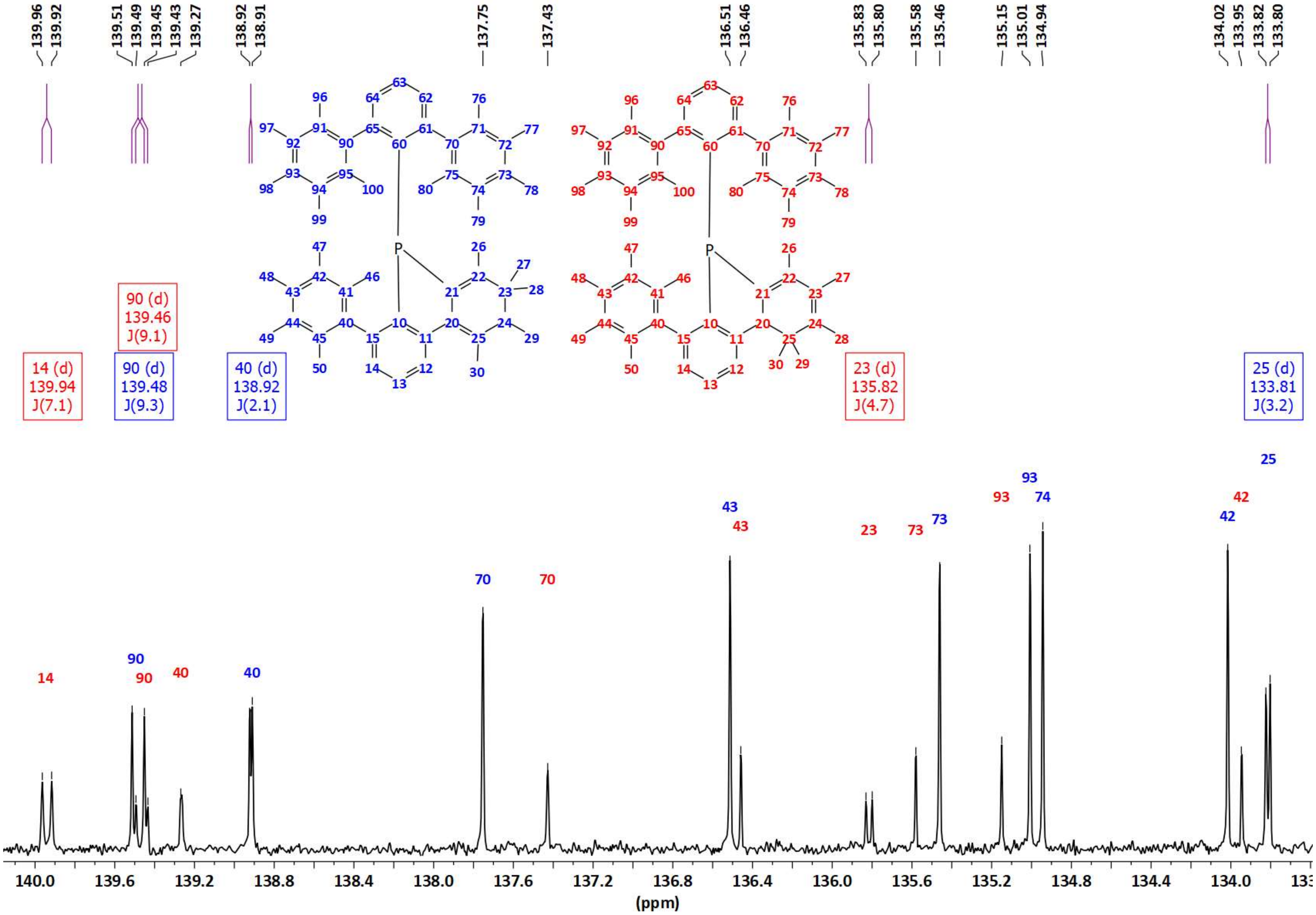

Figure S14. ^{13}C NMR (CD_2Cl_2, 151 MHz) spectrum (detail) of [**5a**]$^+$ and [**7a**]$^+$.

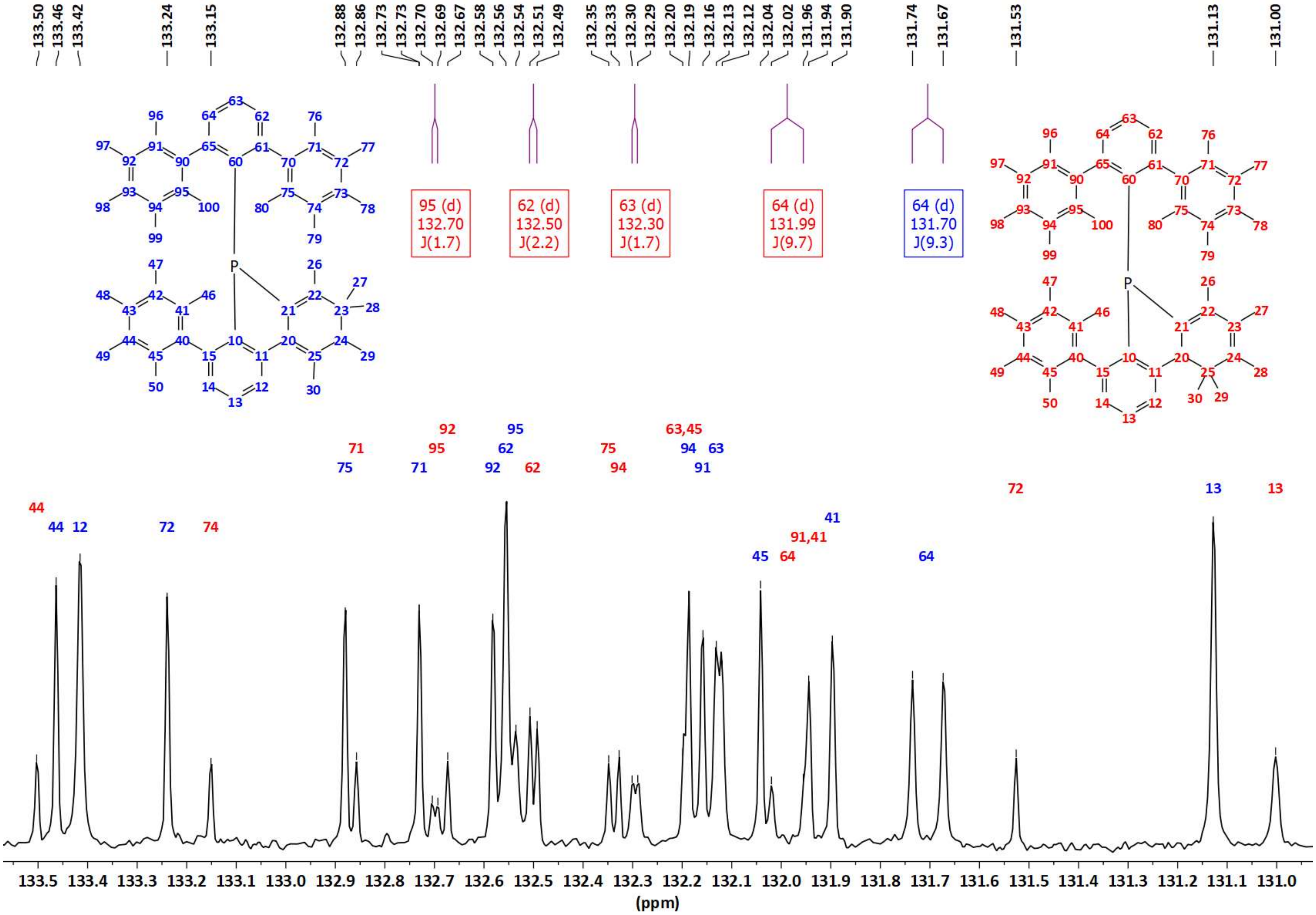

Figure S15. ^{13}C NMR (CD_2Cl_2, 151 MHz) spectrum (detail) of [**5a**]$^+$ and [**7a**]$^+$.

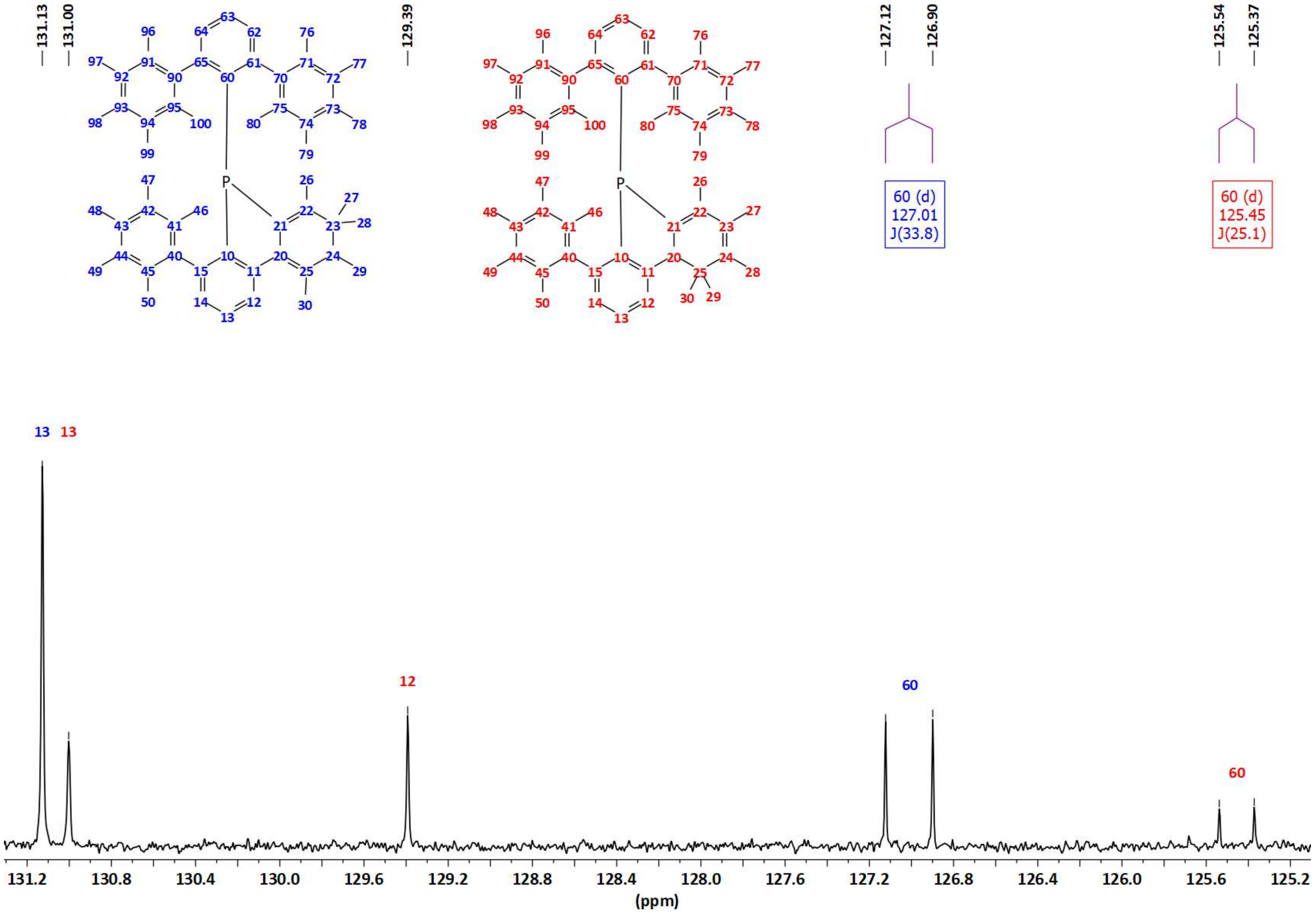

Figure S16. ^{13}C NMR (CD_2Cl_2, 151 MHz) spectrum (detail) of [**5a**]$^+$ and [**7a**]$^+$.

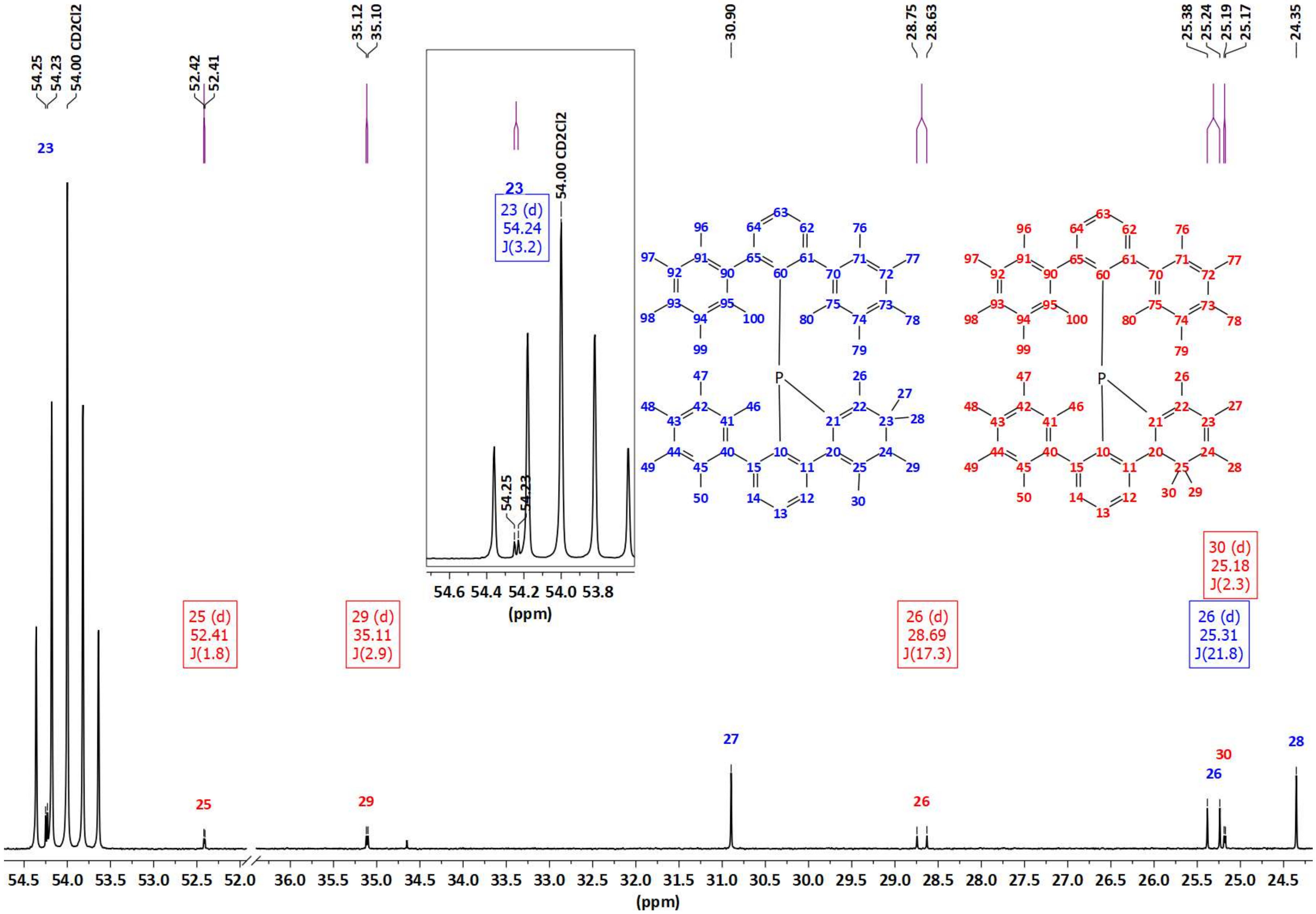

Figure S17. ^{13}C NMR (CD_2Cl_2, 151 MHz) spectrum (detail) of [**5a**]$^+$ and [**7a**]$^+$.

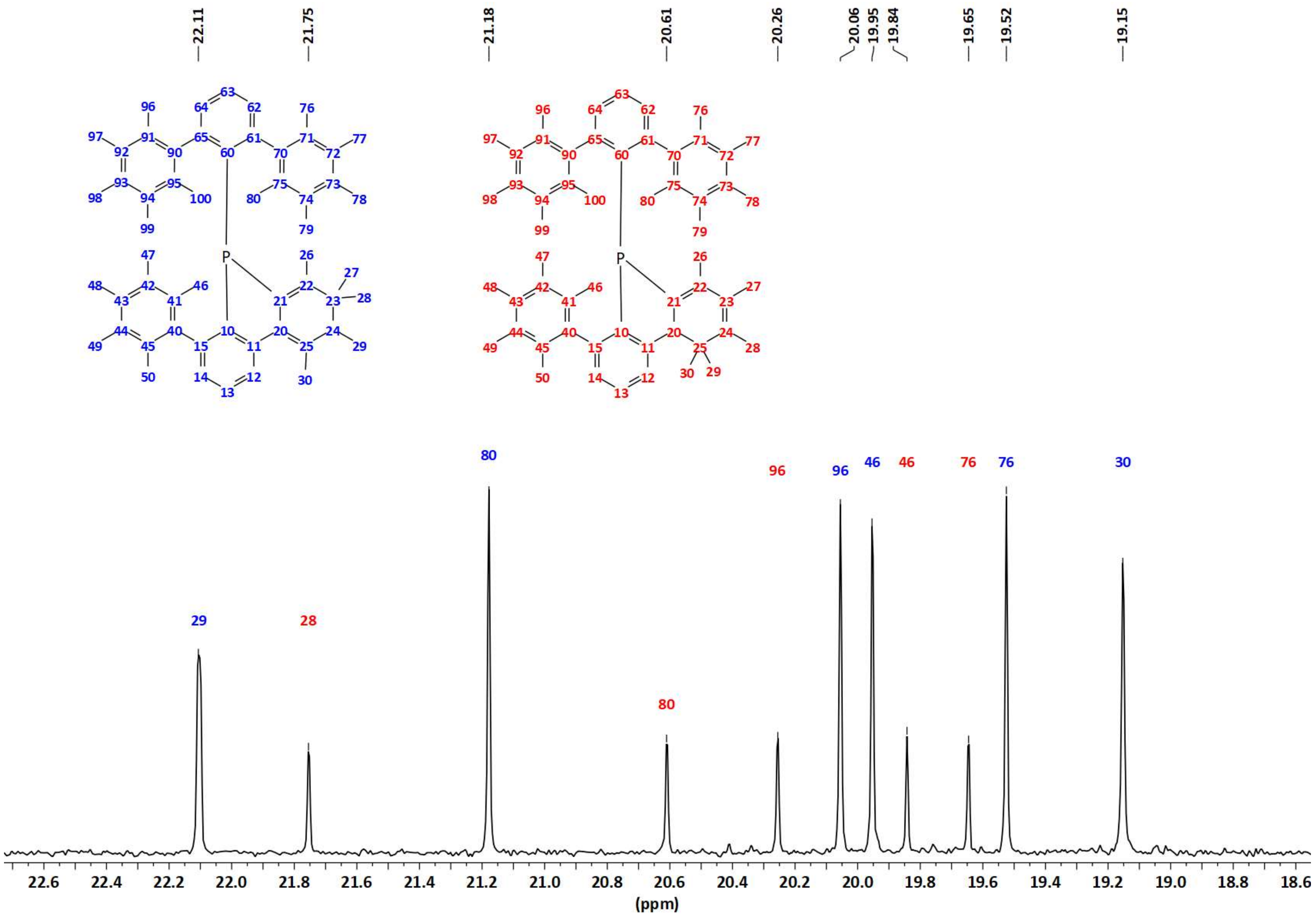

Figure S18. ^{13}C NMR (CD_2Cl_2, 151 MHz) spectrum (detail) of [**5a**]$^+$ and [**7a**]$^+$.

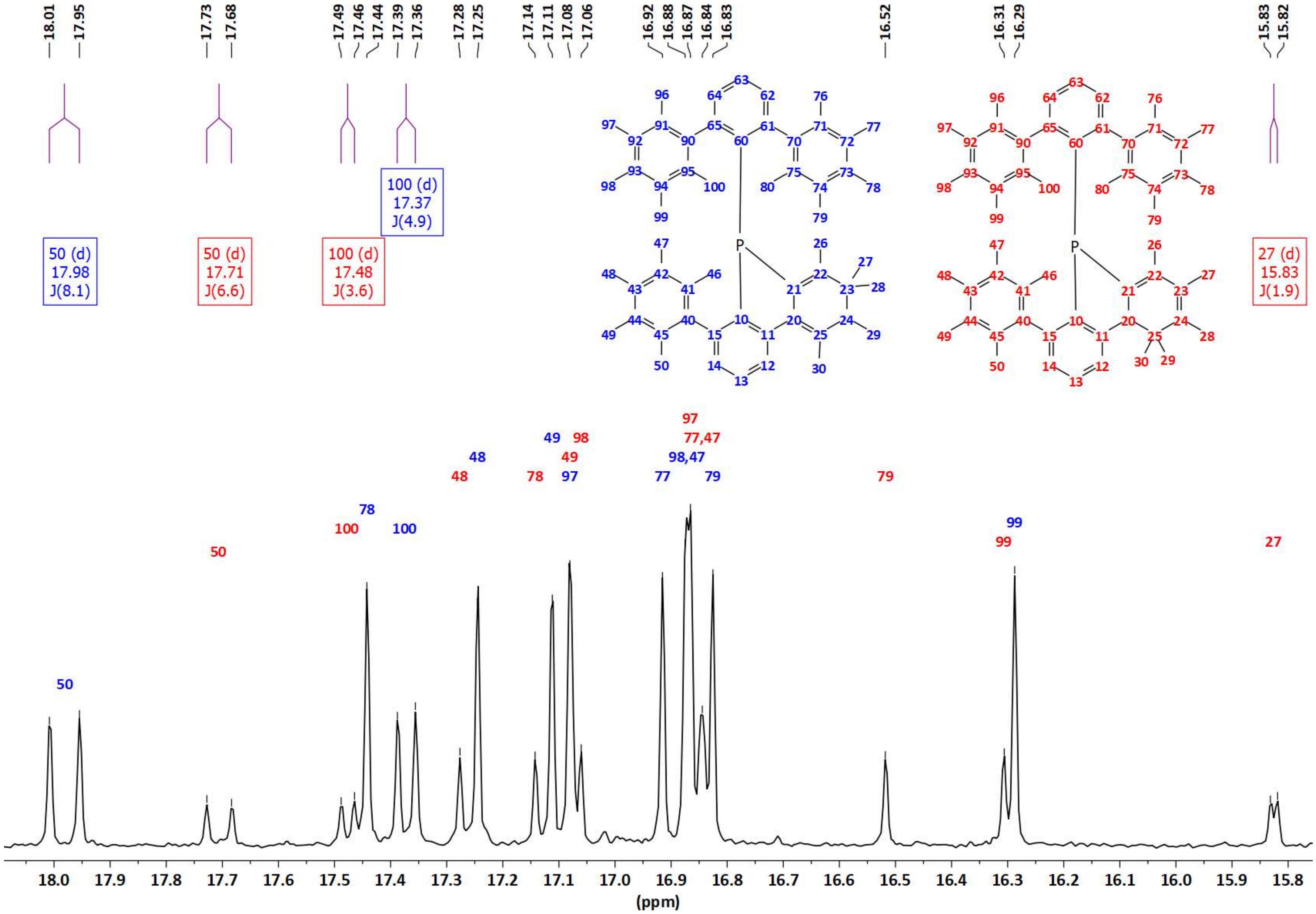

Figure S19. ^{13}C NMR (CD_2Cl_2, 151 MHz) spectrum (detail) of [**5a**]$^+$ and [**7a**]$^+$.

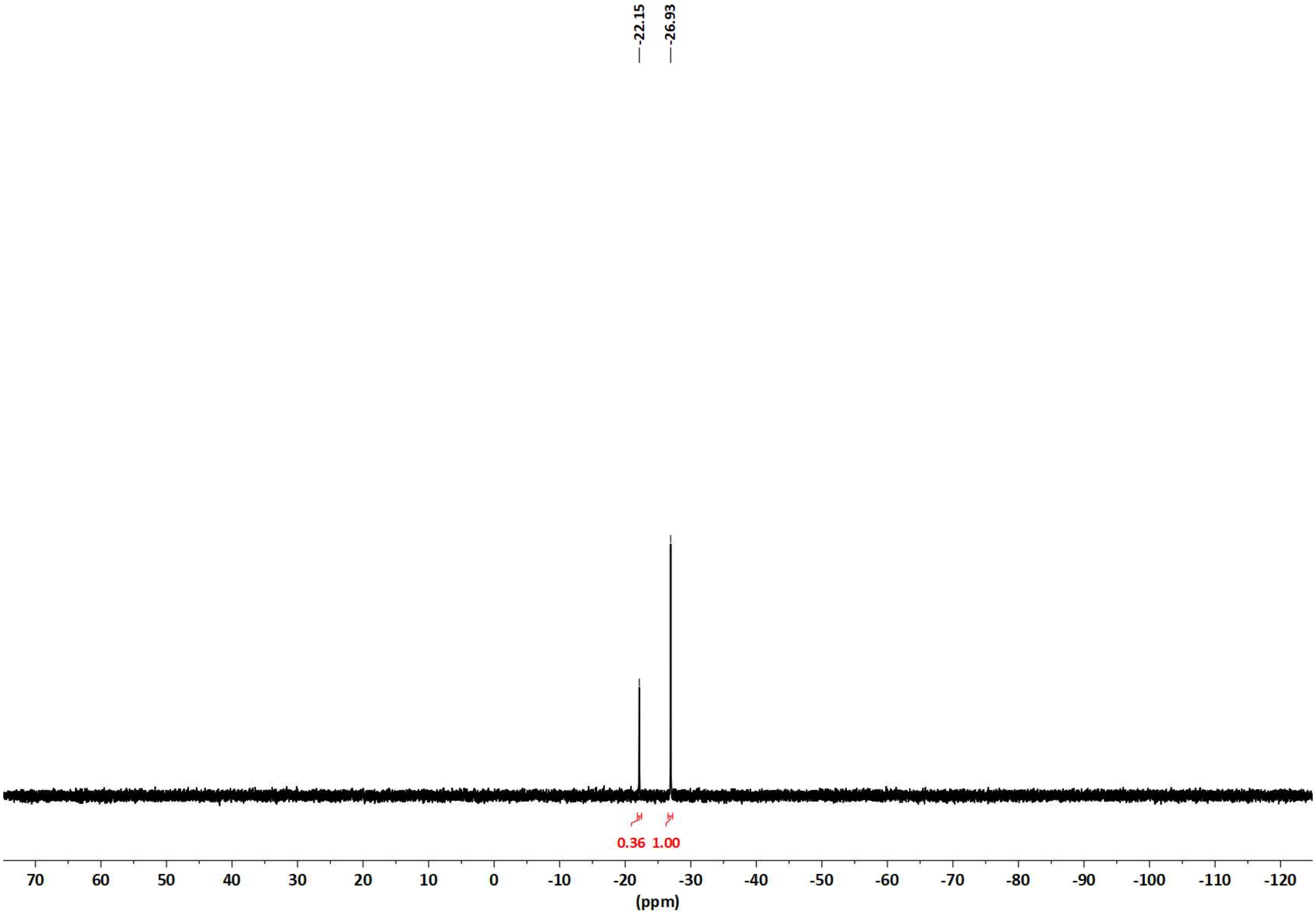

Figure S20. $^{31}P\{1H\}$ NMR (CD_2Cl_2, 243 MHz) spectrum of [**5a**]$^+$ and [**7a**]$^+$.

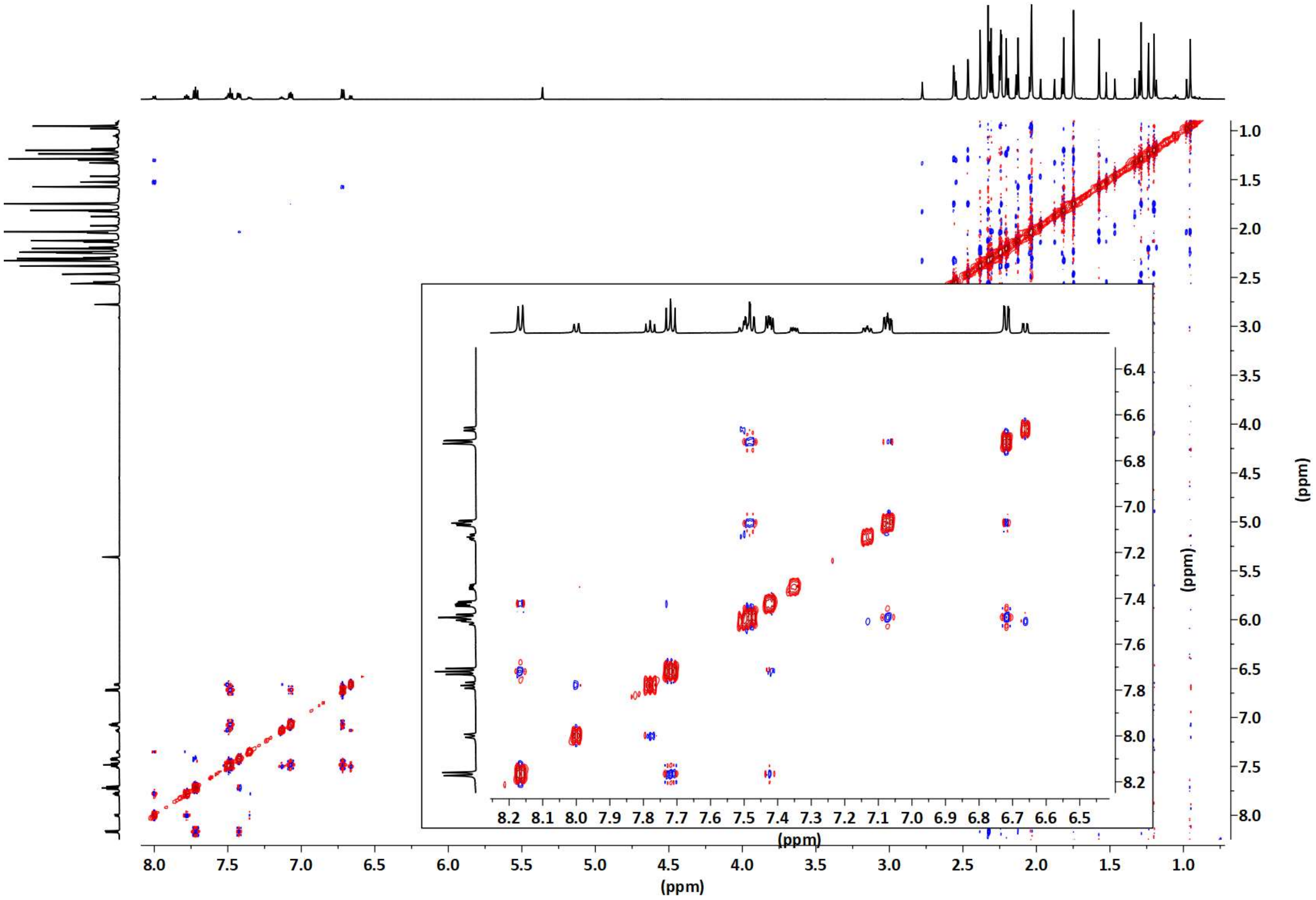

Figure S21. NOESY (CD_2Cl_2, 600 MHz) spectrum showing no exchange between $[\mathbf{5a}]^+$ and $[\mathbf{7a}]^+$.(mixing time = 0.2 s).

Synthesis and characterization of [5b][$AlCl_4$] (and [7b][$AlCl_4$])

1b (50 mg, 0.06 mmol) and $AlCl_3$ (8 mg, 0.06 mmol) were placed in a Schlenk tube and dry CH_2Cl_2 (4 mL) was added. Upon addition of the solvent the reaction turns immediately deep red. The solution was stirred for 1h at room temperature and after that it was filtered through a syringe filter to remove any insoluble materials that might form. The solution was layered with hexane (20 mL) and after diffusion the solvent was removed. The remaining crystalline solid was dried under vacuum to yield a deep red crystalline solid (58 mg, quantitative yield). NMR analysis indicated that always a mixture of [**5b**][$AlCl_4$] and [**7b**][$AlCl_4$] in a molar ratio of *ca.* 0.70:0.30 was obtained. From all crystallization experiments only [**5b**][$AlCl_4$] produced crystals suitable for single-crystal X-ray structure determination. The identity of [**7b**][$AlCl_4$] was inferred from NMR spectra.

Major isomer [**5b**]$^+$:

^{1}H NMR (600 MHz, CD_2Cl_2): δ = 8.20 (d, $^3J(^1H–^1H)$ = 8.30 Hz, 1H, H12), 7.68 (t, $^3J(^1H–^1H)$ = 7.65 Hz, 1H, H13), 7.40 (d, $^3J(^1H–^1H)$ = 8 Hz, 1H, H14), 7.38 (t, $^3J(^1H–^1H)$ = 7.50 Hz, 1H, H63), 7.00 (d, $^3J(^1H–^1H)$ = 7.50 Hz, $^4J(^1H–^1H)$ = 1.30 Hz, 1H, H64), 6.59 (d, $^3J(^1H–^1H)$ = 7.50 Hz, $^4J(^1H–^1H)$ = 1.30 Hz, 1H, H62), 2.53 (s, 3H, H26), 2.48 (s, 3H, H29), 2.36 (s, 3H, H48), 2.29 (s, 3H, H30), 2.28 (s, 3H, H78), 2.27 (s 3H, H98), 2.23 (s, 3H, H49), 2.21 (s, 3H, H97), 2.16 (s, 3H, H47), 2.08 (s, 3H, H46), 2.06 (s, 3H, H79), 1.98 (s, 3H, H99), 1.81 (s, 3H, H77) 1.79 (s, 3H, H96) 1.71 (s, 3H, H27), 1.50 (s, 3H, H80), 1.29 (s, 3H, H28), 1.28 (s, 3H, H50) 1.21 (s, 3H, H76), 0.97 (s, 3H, H100) ppm. **^{13}C NMR (151 MHz, CD_2Cl_2)**: δ = 192.08 (s, C22), 190.71 (s, C24), 179.49 (s, C20), 160.21 (s, C10), 152.75 (s, C65), 150.90 (s, C11), 149.58 (s, C61), 147.62 (s, C15), 146.76 (s, C21), 141.68 (s, C14), 139.74 (s, C90), 139.07 (s, C40), 137.94 (s, C70), 136.52 (s, C43), 135.37 (s, C73), 135.06 (s, C93), 134.80 (s, C25), 134.68 (s, C12), 134.36 (s, C74), 134.18 (s, C45), 133.63 (s, C41), 132.94 (s, C75), 132.84 (s, C71), 132.77 (s, C91), 132.57 (s, C95), 132.54 (s, C60), 132.35 (s, C72), 132.31 (s, C94), 132.22 (s, C62), 132.14 (s, C42), 131.83 (s, C44), 131.71 (s, C92), 131.38 (s, C13 and C63), 131.12 (s, C64), 54.27 (s, C23), 30.95 (s, C28), 27.05 (s, C29), 24.46 (s, C27), 22.46 (s, C26), 21.15 (s, C80), 20.00 (s, C50), 19.88 (s, C30), 19.85 (s, C96), 19.48 (s, C76), 18.42 (s, C46), 17.50 (s, C78), 17.30 (s, C48), 17.24 (s, C100), 17.19 (s, C98), 17.13 (s, C47), 16.95 (s, C49), 16.91 (s, C77), 16.85 (s, C97), 16.73 (s, C79), 16.32 (s, C99) ppm.

Minor isomer [**7b**]$^+$:

^{1}H NMR (600 MHz, CD_2Cl_2): *δ* = 7.96 (d, $^3J(^1H–^1H)$ = 7.80 Hz, H12), 7.73 (t, $^3J(^1H–^1H)$ = 7.80 Hz, H13), 7.37 (m, H63), 7.33 (d, $^3J(^1H–^1H)$ = 7.80 Hz, H14), 7.04 (dd, $^3J(^1H–^1H)$ = 7.50 Hz, $^4J(^1H–^1H)$ = 1.30 Hz, H64), 6.54 ($^3J(^1H–^1H)$ = 7.50 Hz, $^4J(^1H–^1H)$ = 1.30 Hz, H62), 2.78 (s, 3H,

H26), 2.53 (s, 3H, H28), 2.36 (s, 3H, H48), 2.31 (s, 3H, H27), 2.28 (s, 3H, H78), 2.27 (s, 3H, H98), 2.23 (s, 3H, H49), 2.22 (s, 3H, H97), 2.15 (s, 3H, H47), 2.11 (s, 3H, H50), 2.10 (s, 3H, H99), 1.92 (s, 3H, H79), 1.85 (s, 6H, H77 and H96), 1.50 (s, 3H, H30), 1.41 (s, 3H, H80), 1.32 (s, 3H, H29), 1.32 (s, 3H, H76), 1.24 (s, 3H, H46), 0.95 (s, 3H, H100) ppm. **^{13}C{^{1}H} NMR (151 MHz, CD_2Cl_2)**: δ = 196.62 (s, C20), 190.75 (s, C24), 183.96 (s, C22), 154.97 (s, C10), 152.94 (s, C65), 150.71 (s, C11), 150.31 (s, C21), 150.24 (s, C61), 147.62 (s, C15), 139.85 (s, C90), 139.42 (s, C40), 139.39 (s, C14), 137.80 (s, C70), 136.46 (s, C43), 135.56 (s, C23), 135.42 (s, C73), 135.14 (s, C93), 134.15 (s, C45), 133.63 (s, C41), 132.84 (s, C95 and C72), 132.77 (s, C75), 132.57 (s, C94), 132.47 (s, C74), 132.39 (s, C62), 132.16 (s, C71), 132.14 (s, C91), 131.92 (s, C42), 131.89 (s, C44), 131.71 (s, C60), 131.46 (s, C64), 131.42 (s, C92), 131.38 (s, C63) 130.87 (s. C13), 130.87 (s, C12), 53.59 (s, C25), 35.91 (s, C29), 30.56 (s, C26), 25.36 (s, C30), 22.11 (s, C28), 20.78 (s, C80), 20.00 (s, C50 and C96), 19.62 (s, C76), 18.24 (s, C46), 17.31 (s, C78), 17.26 (s, C48), 17.19 (s, C98), 17.16 (s, C100), 17.13 (s, C47), 17.09 (s, C99), 16.95 (s, C49), 16.85 (s, C97), 16.73 (s, C77), 16.41 (s, C79), 15.93 (s, C27) ppm.

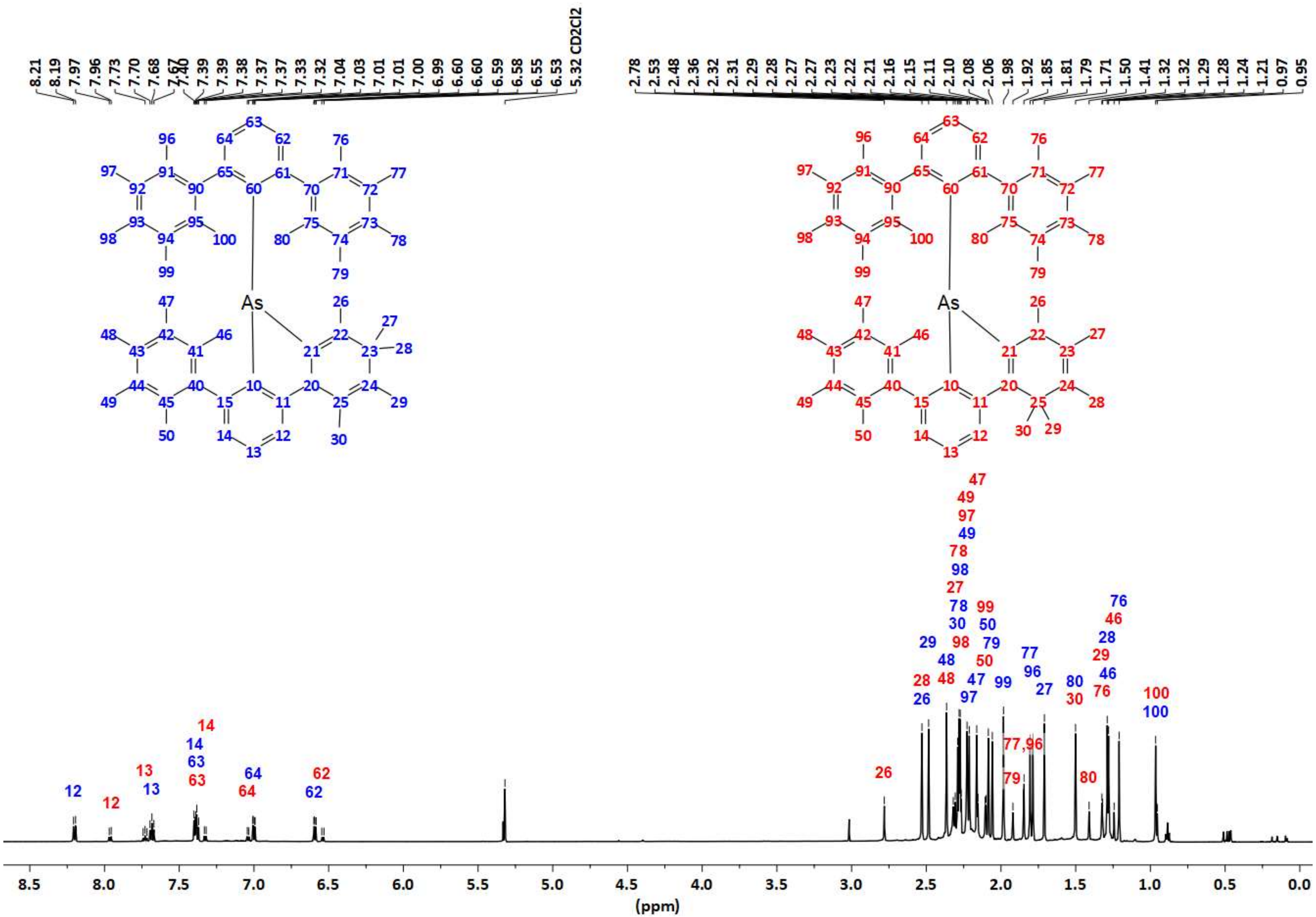

Figure S22. ^{1}H NMR (CD_2Cl_2, 600 MHz) spectrum of [**5b**]$^{+}$ and [**7b**]$^{+}$.

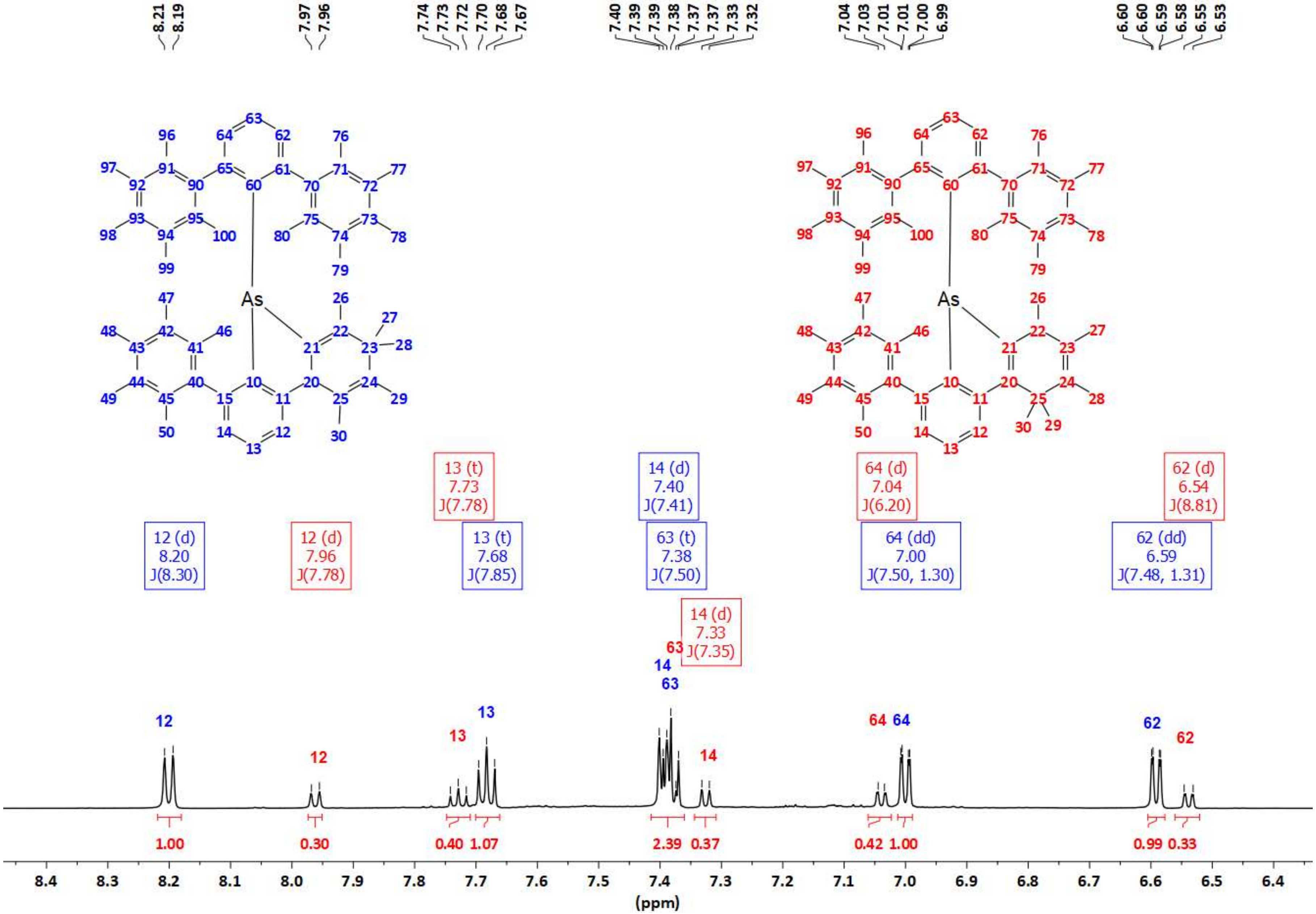

Figure S23. ^{1}H NMR (CD_2Cl_2, 600 MHz) spectrum (detail) of [**5b**]$^+$ and [**7b**]$^+$.

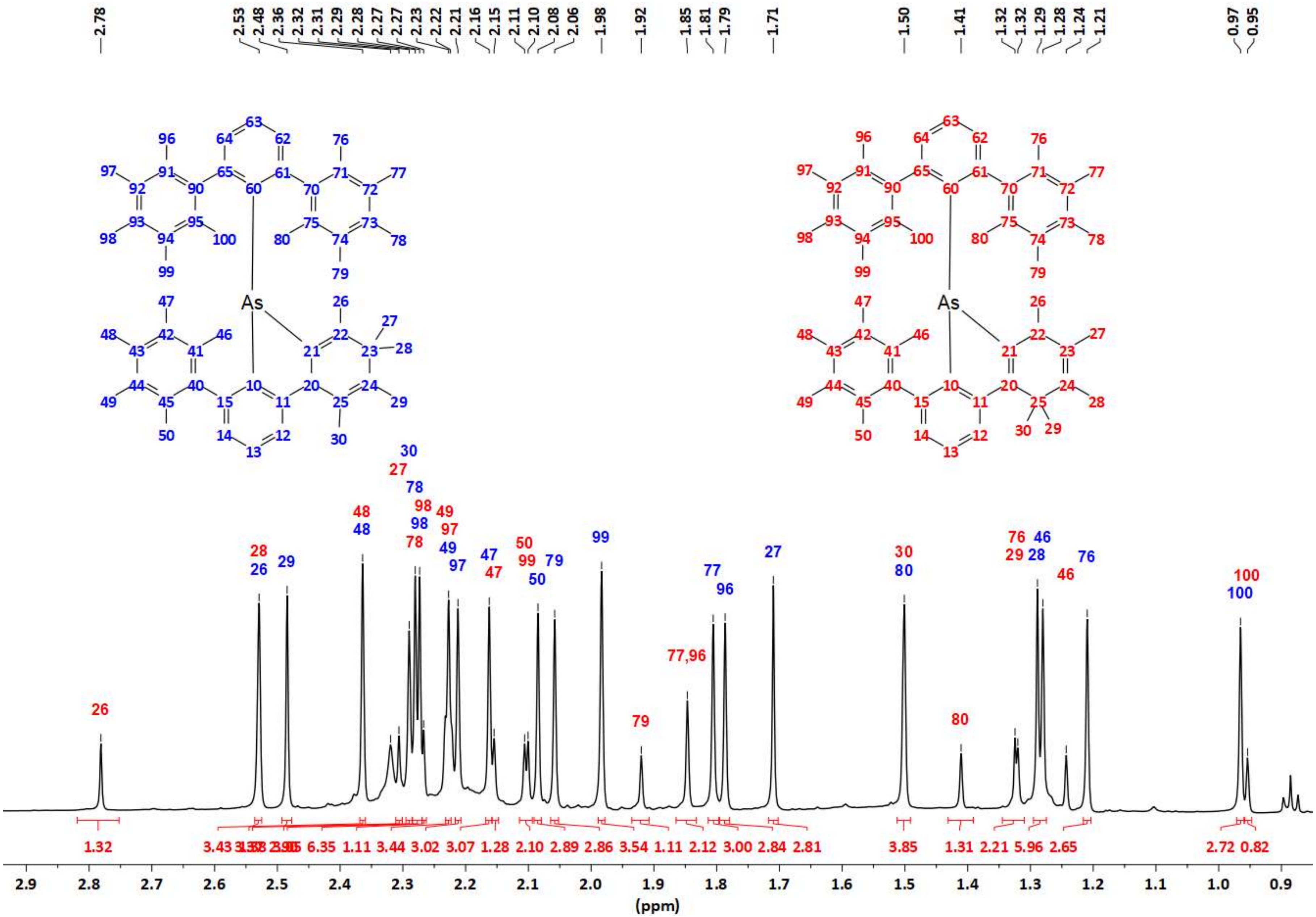

Figure S24. ^{1}H NMR (CD_2Cl_2, 600 MHz) spectrum (detail) of [**5b**]$^{+}$ and [**7b**]$^{+}$.

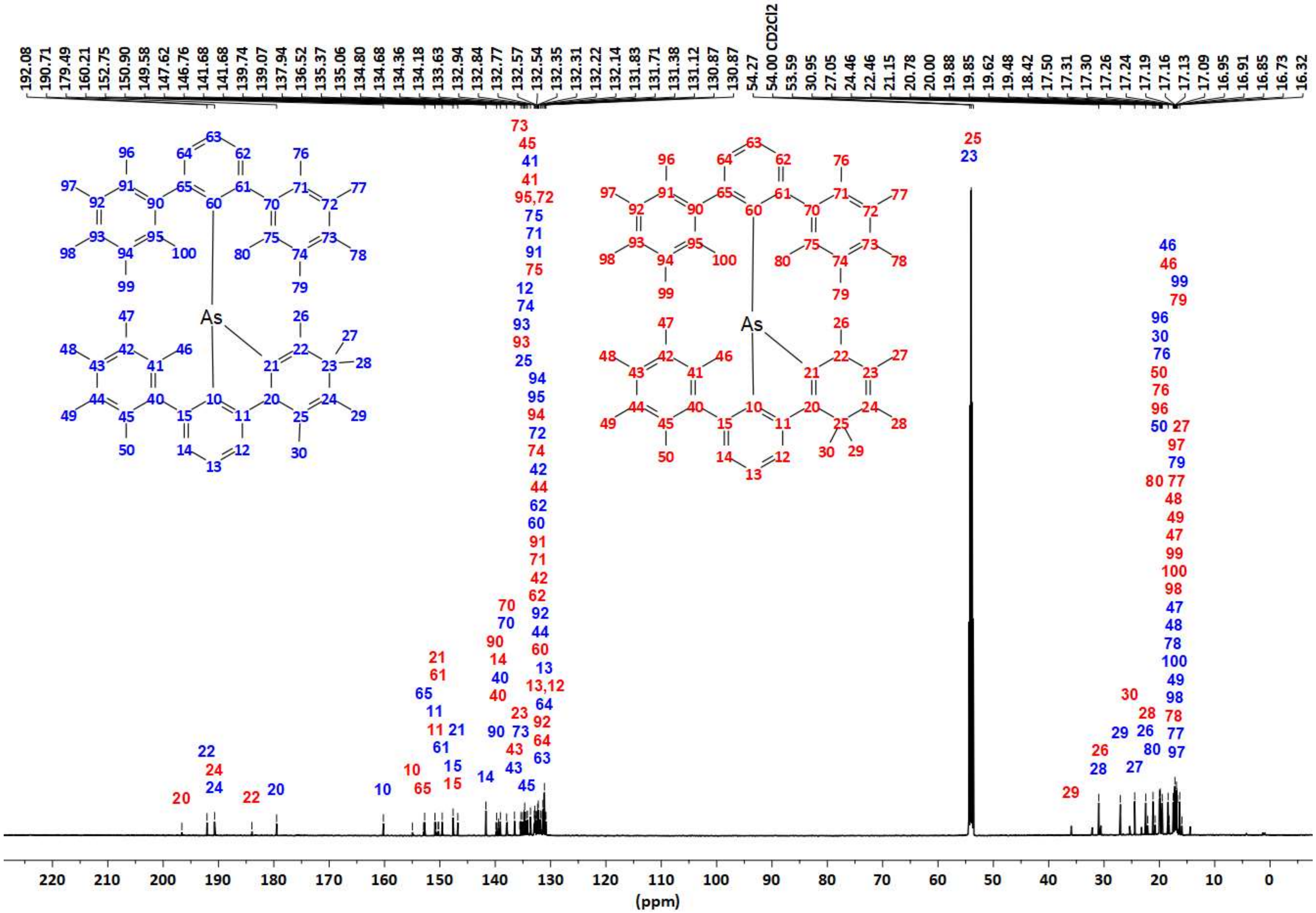

Figure S25. ^{13}C NMR (CD_2Cl_2, 151 MHz) spectrum of [**5b**]$^+$ and [**7b**]$^+$.

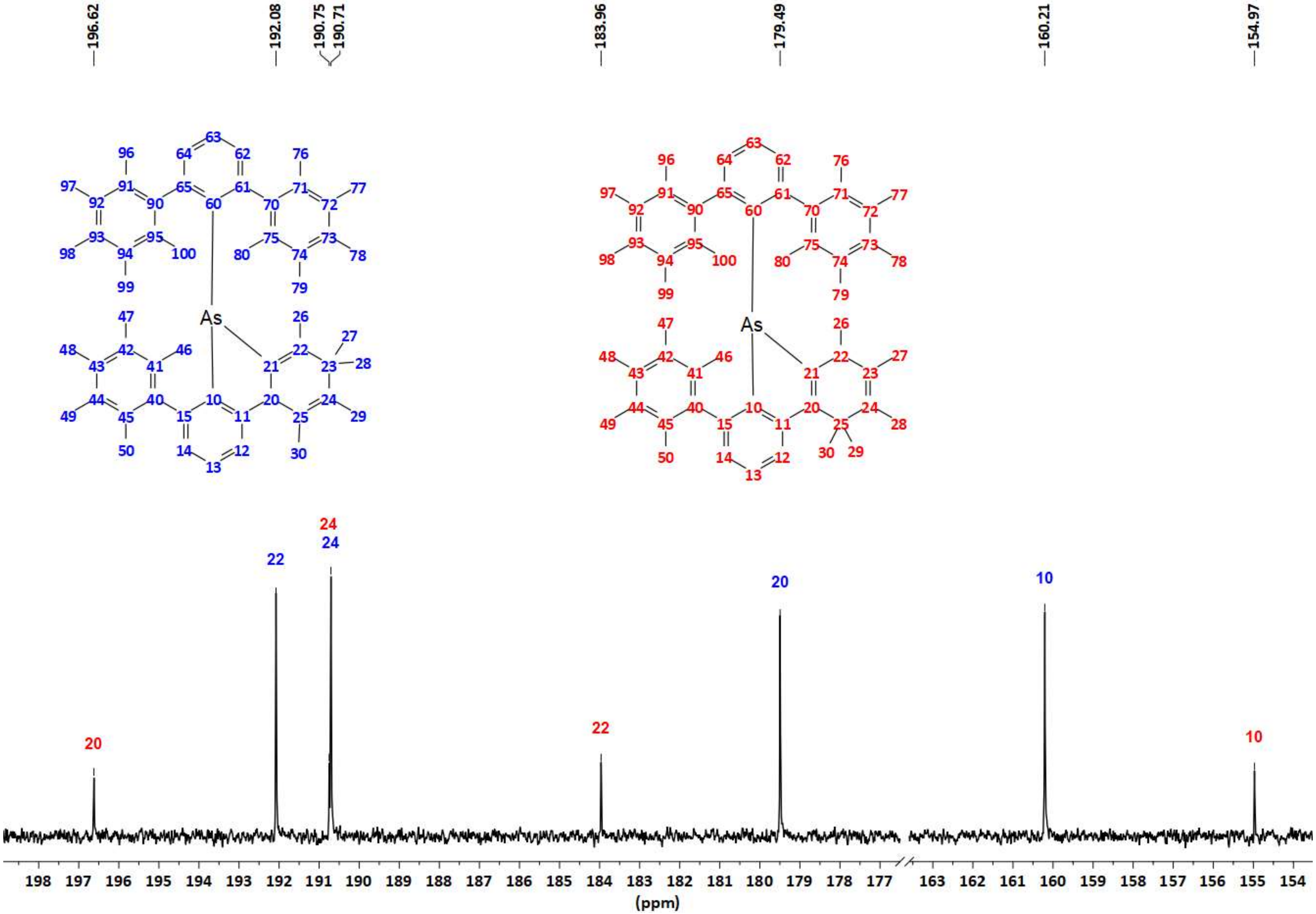

Figure S26. ^{13}C NMR (CD_2Cl_2, 151 MHz) spectrum (detail) of [**5b**]$^{+}$ and [**7b**]$^{+}$.

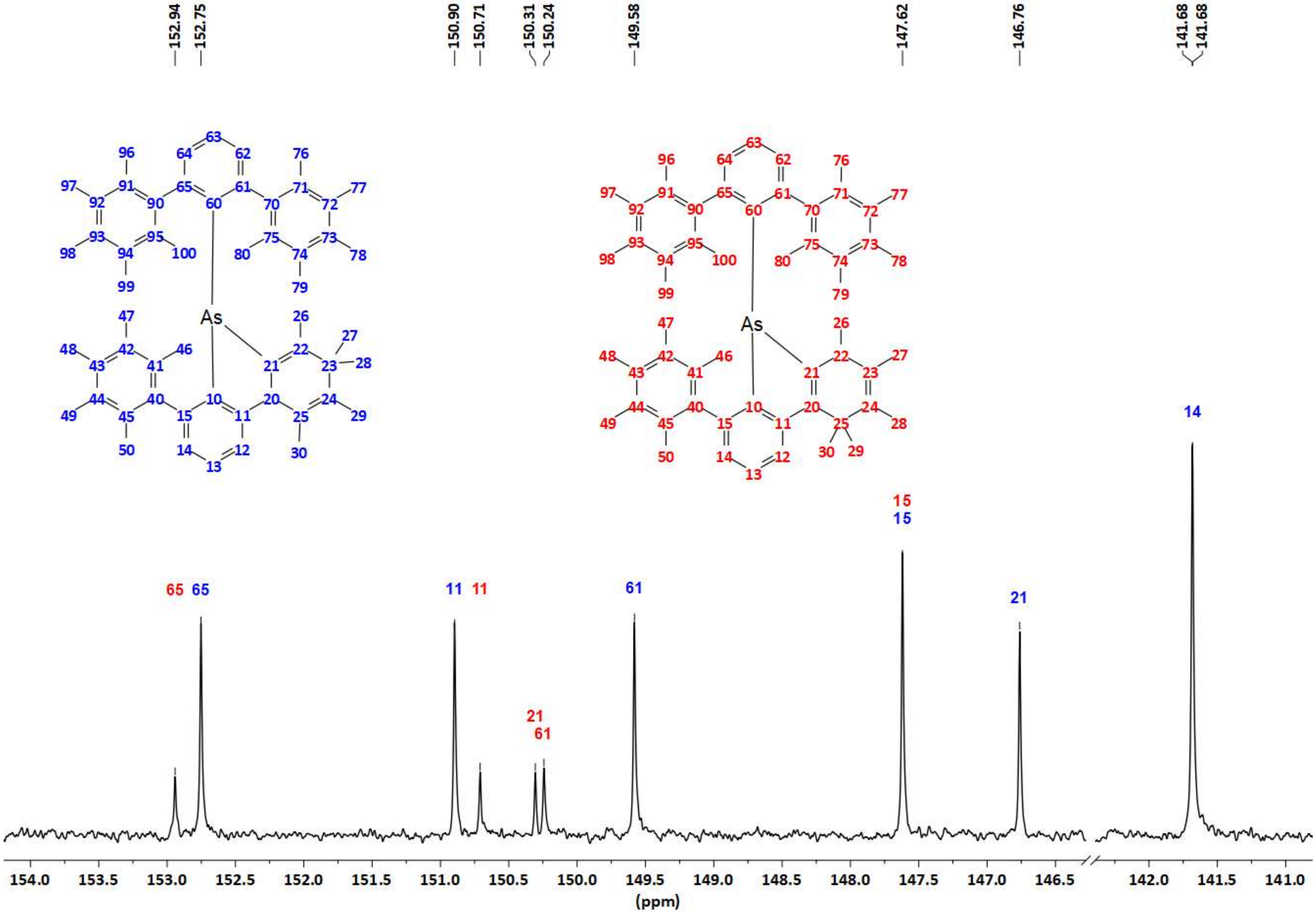

Figure S27. ^{13}C NMR (CD_2Cl_2, 151 MHz) spectrum (detail) of [**5b**]$^+$ and [**7b**]$^+$.

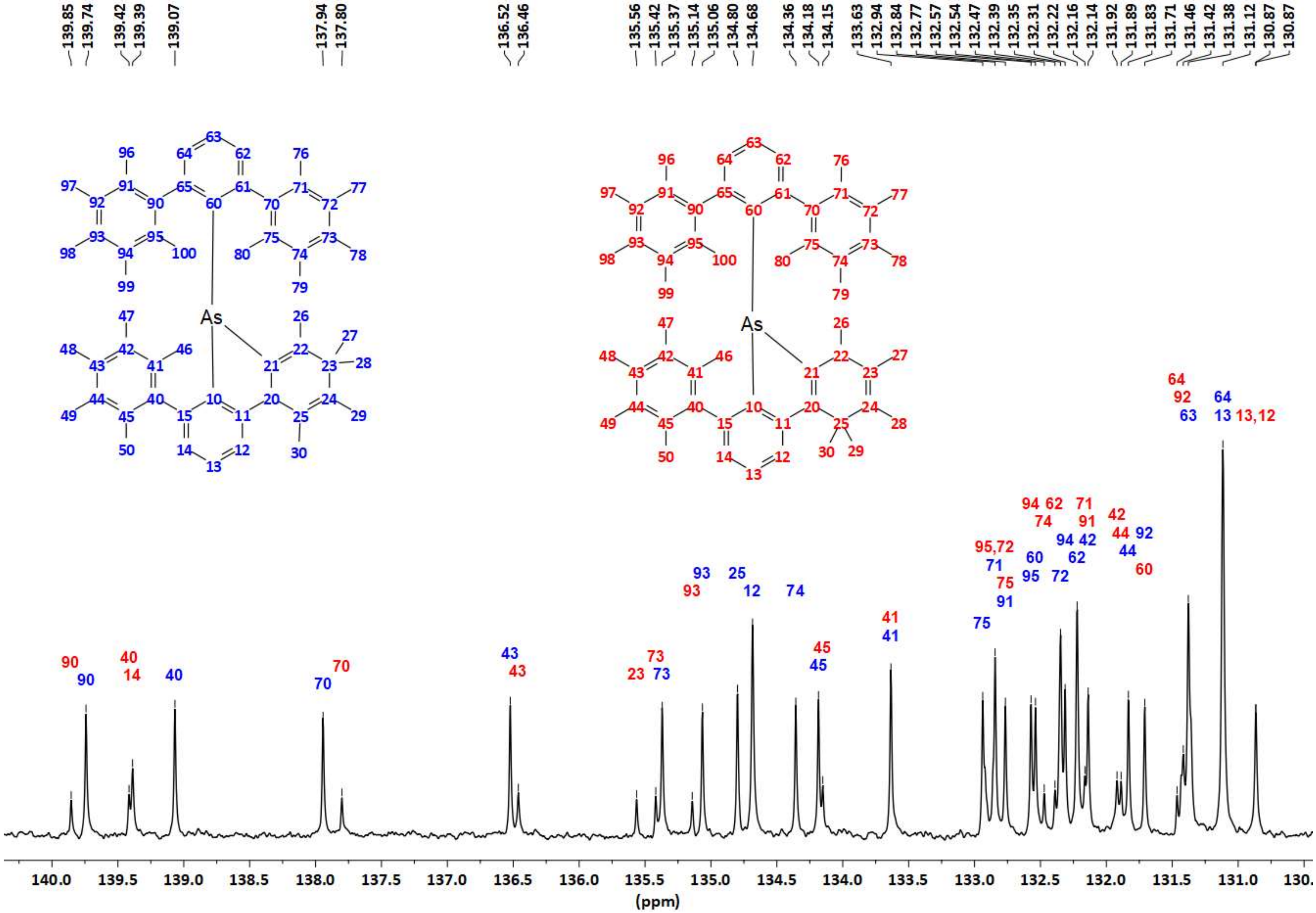

Figure S28. ^{13}C NMR (CD_2Cl_2, 151 MHz) spectrum (detail) of [**5b**]$^+$ and [**7b**]$^+$.

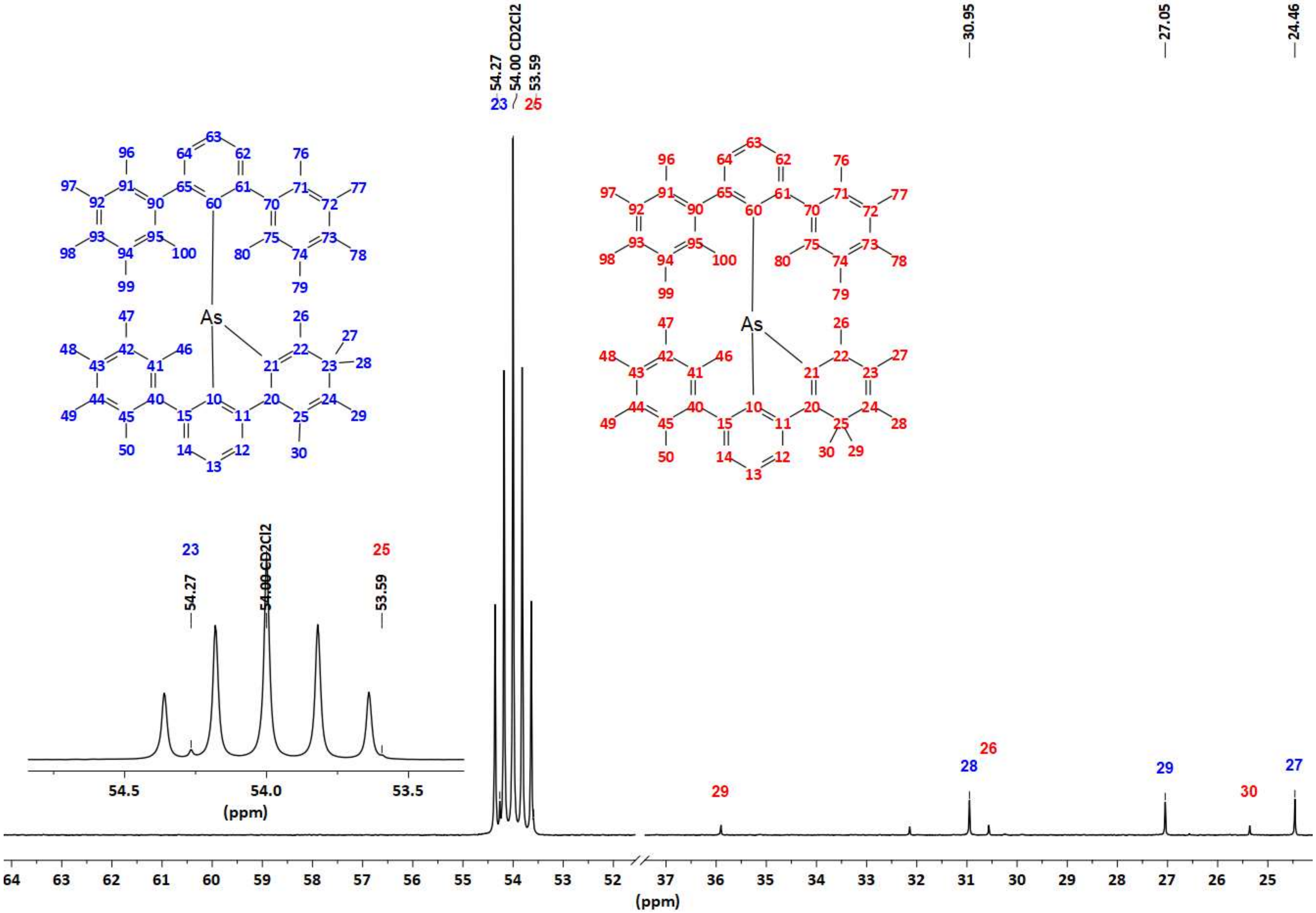

Figure S29. ^{13}C NMR (CD_2Cl_2, 151 MHz) spectrum (detail) of [**5b**]$^+$ and [**7b**]$^+$.

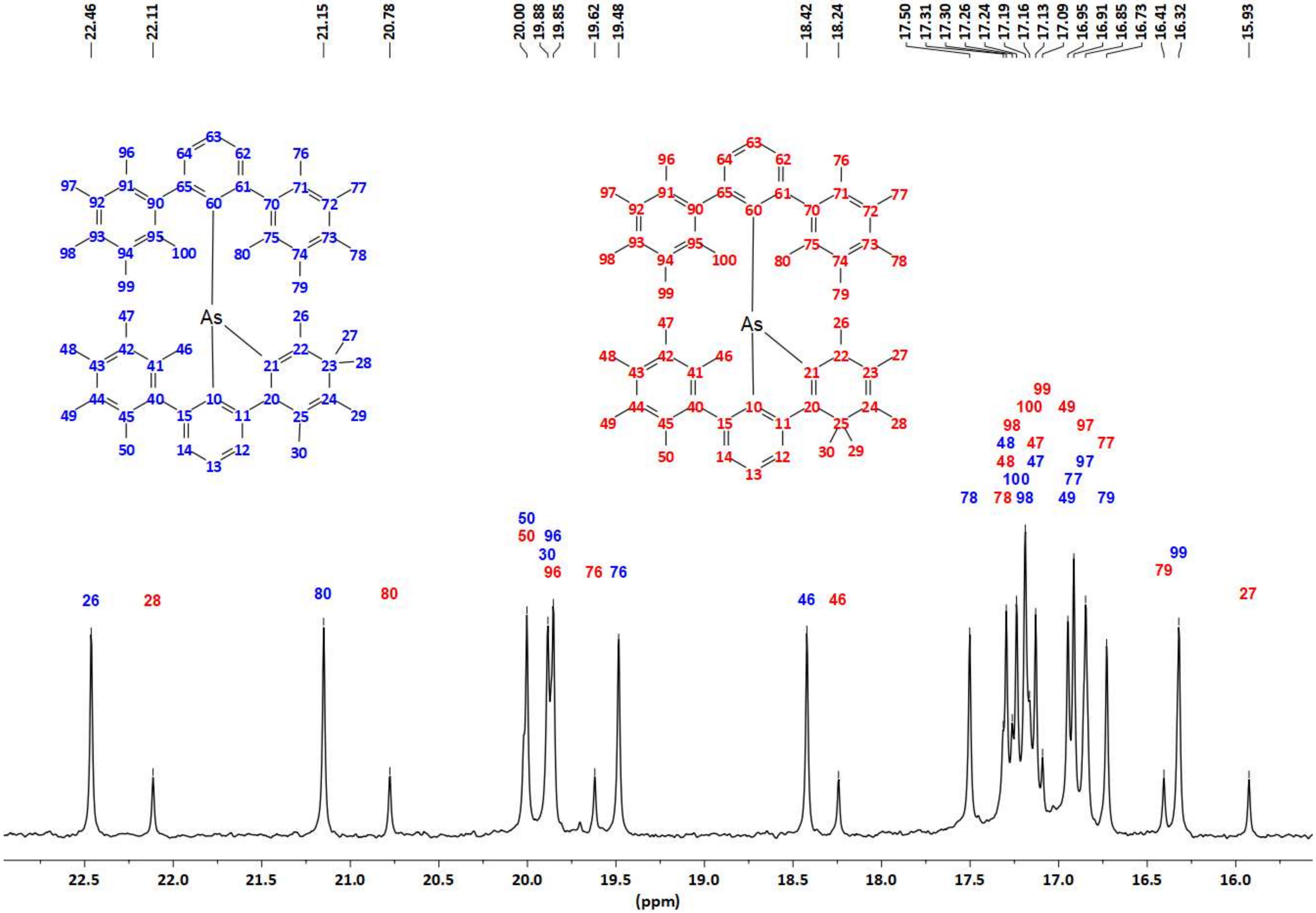

Figure S30. ^{13}C NMR (CD_2Cl_2, 151 MHz) spectrum (detail) of [**5b**]$^+$ and [**7b**]$^+$.

Synthesis and characterization of [8a][AlCl4]

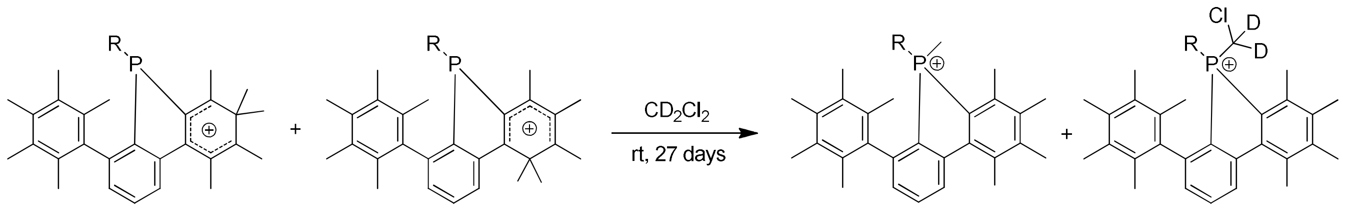

[5a]+ [7a]+ [8a]+ Major product [10a]+ Trace by-product

R = 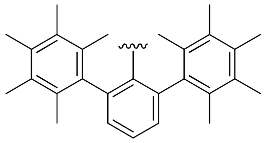

A J. Young NMR tube charged with a mixture of [**5a**][AlCl4] and [**7a**][AlCl4] (29 mg), in CD_2Cl_2 (0.6 mL) was allowed to stand under argon at room temperature in ambient light for *ca.* 1 month during which time the reaction was monitored by NMR spectroscopy.[§] Compound [**8a**][AlCl4] was the major product that was observed. A minor byproduct ([**10a**]$^+$) formed as well. Crystals suitable for single crystal X-ray diffraction (20 mg) were grown by diffusing hexane into the CD_2Cl_2 solution. Neither multiple re-crystallizations nor other attempts (including chromatography) were successful at obtaining [**8a**][AlCl4] as a pure compound. **^{1}H NMR (600 MHz, CD_2Cl_2)**: δ = 7.89 (dd, $^{3}J(^{1}H–^{1}H)$ = 8 Hz, $^{4}J(^{1}H–^{31}P)$ = 3 Hz, 1H, H12), 7.76 (m, 1H, H13), 7.74 (dd, $^{3}J(^{1}H–^{1}H)$ = 8 Hz, $^{5}J(^{1}H–^{31}P)$ = 2 Hz, 1H, H63), 7.28 (ddd, $^{3}J(^{1}H–^{1}H)$ = 8 Hz, $^{4}J(^{1}H–^{1}H)$ = 5 Hz, $^{4}J(^{1}H–^{31}P)$ = 2 Hz, 1H, H64), 7.03 (ddd, $^{3}J(^{1}H–^{1}H)$ = 8 Hz, $^{4}J(^{1}H–^{1}H)$ = 5 Hz, $^{4}J(^{1}H–^{31}P)$ = 2 Hz, 1H, H62), 6.84 (ddd, $^{3}J(^{1}H–^{1}H)$ = 8 Hz, $^{4}J(^{1}H–^{1}H)$ = 5 Hz, $^{4}J(^{1}H–^{31}P)$ = 1 Hz, 1H, H14), 2.36 (s, 3H, H48), 2.34 (s, 6H, H29, H28), 2.32 (s, 3H, H26), 2.28 (s, 3H, H98), 2.25 (s, 3H, H27), 2.22 (s, 3H, H99), 2.19 (s, 3H, H47), 2.18 (s, 6H, H49, H78), 2.05 (s, 3H, H77), 2.00 (s, 3H, H97), 1.77 (s, 3H, H100), 1.74 (s, 3H, H46), 1.66 (s, 3H, H79), 1.39 (s, 3H, H76), 1.34 (s, 3H, H50), 1.27 (d, $^{4}J(^{1}H–^{31}P)$ = 13 Hz, 3H, H1), 1.26 (s, 3H, H80), 1.11 (s, 3H, H96) ppm. **^{13}C{^{1}H} NMR (151 MHz, CD_2Cl_2)**: δ = 152.57 (d, $^{2}J(^{13}C–^{31}P)$ = 11 Hz, C61), 152.41 (d, $^{2}J(^{13}C–^{31}P)$ = 11 Hz, C65), 149.82 (d, $^{2}J(^{13}C–^{31}P)$ = 10 Hz, C15), 149.41 (d, $^{2}J(^{13}C–^{31}P)$ = 20 Hz, C11), 144.80 (d, $^{4}J(^{13}C–^{31}P)$ = 3 Hz, C24), 138.82 (d, $^{2}J(^{13}C–^{31}P)$ = 18 Hz, C20), 137.51 (d, $^{3}J(^{13}C–^{31}P)$ = 11 Hz, C23), 137.14 (s, C43), 136.90 (s, C93), 136.89 (d, $^{3}J(^{13}C–^{31}P)$ = 3 Hz, C40), 136.31 (d, $^{3}J(^{13}C–^{31}P)$ = 2 Hz, C90), 136.28 (s, C22), 136.25 (s, C73), 135.78 (d, $^{4}J(^{13}C–^{31}P)$ = 2 Hz, C13), 135.51 (d, $^{3}J(^{13}C–^{31}P)$ = 4 Hz, C70), 135.04 (s, C42), 134.92 (d, $^{4}J(^{13}C–^{31}P)$ = 3 Hz, C63), 134.24 (s, C94), 134.21 (d, $^{3}J(^{13}C–^{31}P)$ = 10 Hz, C64), 134.18 (d, $^{3}J(^{13}C–^{31}P)$ = 10 Hz, C25), 134.14 (d, $^{3}J(^{13}C–^{31}P)$ = 9 Hz, C62), 133.86 (s, C44), 133.30 (s,

[§] A control reaction was ran in parallel in complete darkness. The progression of this reaction was significantly slower, and formation of the P-methylated product appeared to be inhibited.

C74), 133.22 (s, C71), 133.17 (s, C45, C92), 133.09 (d, $^{3}J(^{13}C–^{31}P)$ = 11 Hz, C14), 132.79 (s, C95), 132.68 (s, C75), 132.52 (s, C41), 132.18 (s, C72), 131.81 (s, C91), 125.47 (d, $^{3}J(^{13}C–^{31}P)$ = 10 Hz, C12), 123.05 (d, $^{1}J(^{13}C–^{31}P)$ = 83 Hz, C21), 122.45 (d, $^{1}J(^{13}C–^{31}P)$ = 85 Hz, C10), 116.34 (d, $^{1}J(^{13}C–^{31}P)$ = 82 Hz, C60), 22.51 (d, $^{3}J(^{13}C–^{31}P)$ = 7 Hz, C26), 20.14 (s, C100), 19.76 (d, C46, C76), 19.61 (s, C80), 19.26 (s, C29), 19.23 (s, C50), 18.25 (s, C28), 17.93 (s, C96), 17.40 (s, C78), 17.16 (s, C48), 17.13 (s, C98), 16.98 (s, C77), 16.95 (s, C27, C47), 16.86 (s, C49), 16.80 (s, C99), 16.30 (s, C79), 16.80 (s, C97), 10.30 (d,$^{1}J(^{13}C–^{31}P)$ = 49 Hz, C1) ppm. **$^{31}P\{^{1}H\}$ NMR (243 MHz, CD_2Cl_2)**: δ = 21.68 ppm. **HRMS ESI (m/z)**: $[M]^{+}$ calculated for $C_{56}H_{66}P$, 769.48967; found, 769.48911; $[M-CH_3+CD_2Cl]^{+}$ calculated. for $C_{56}H_{66}D_2PCl$, 805.46325; found, 805.46278.

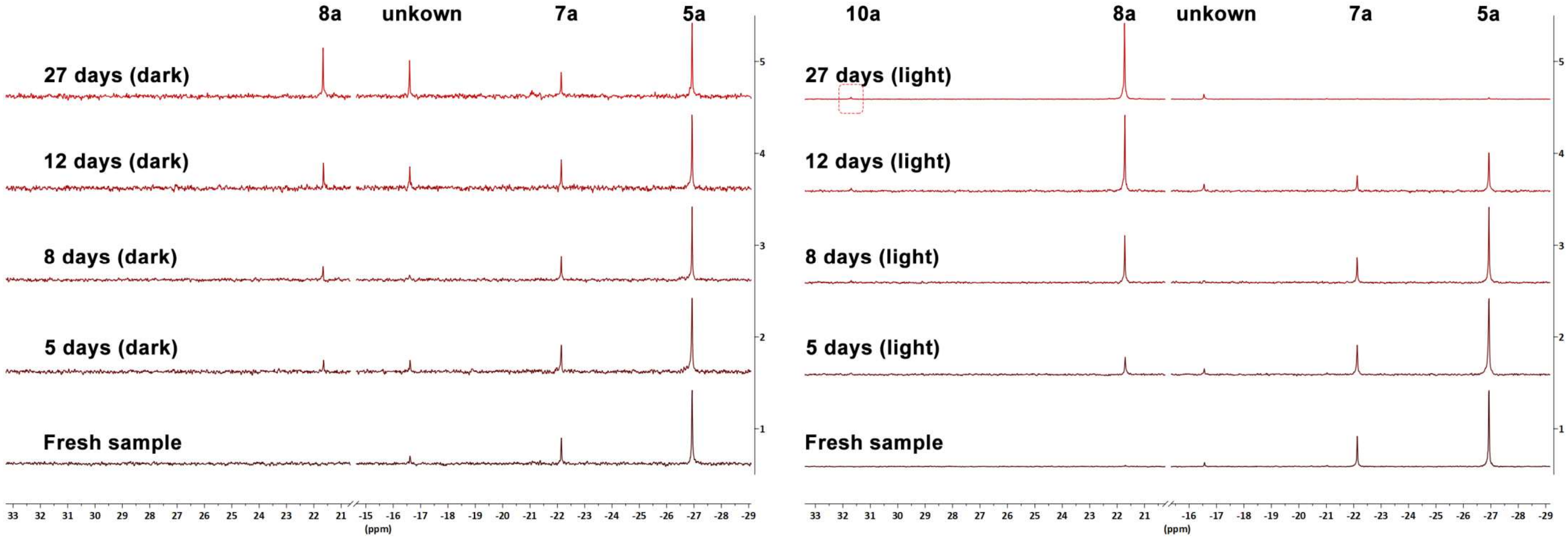

Figure S31. ^{31}P NMR (CD_2Cl_2, 600 MHz) spectra showing the conversion progress of a mixture of [**5a**]$^+$ and [**7a**]$^+$ into [**8a**]$^+$.

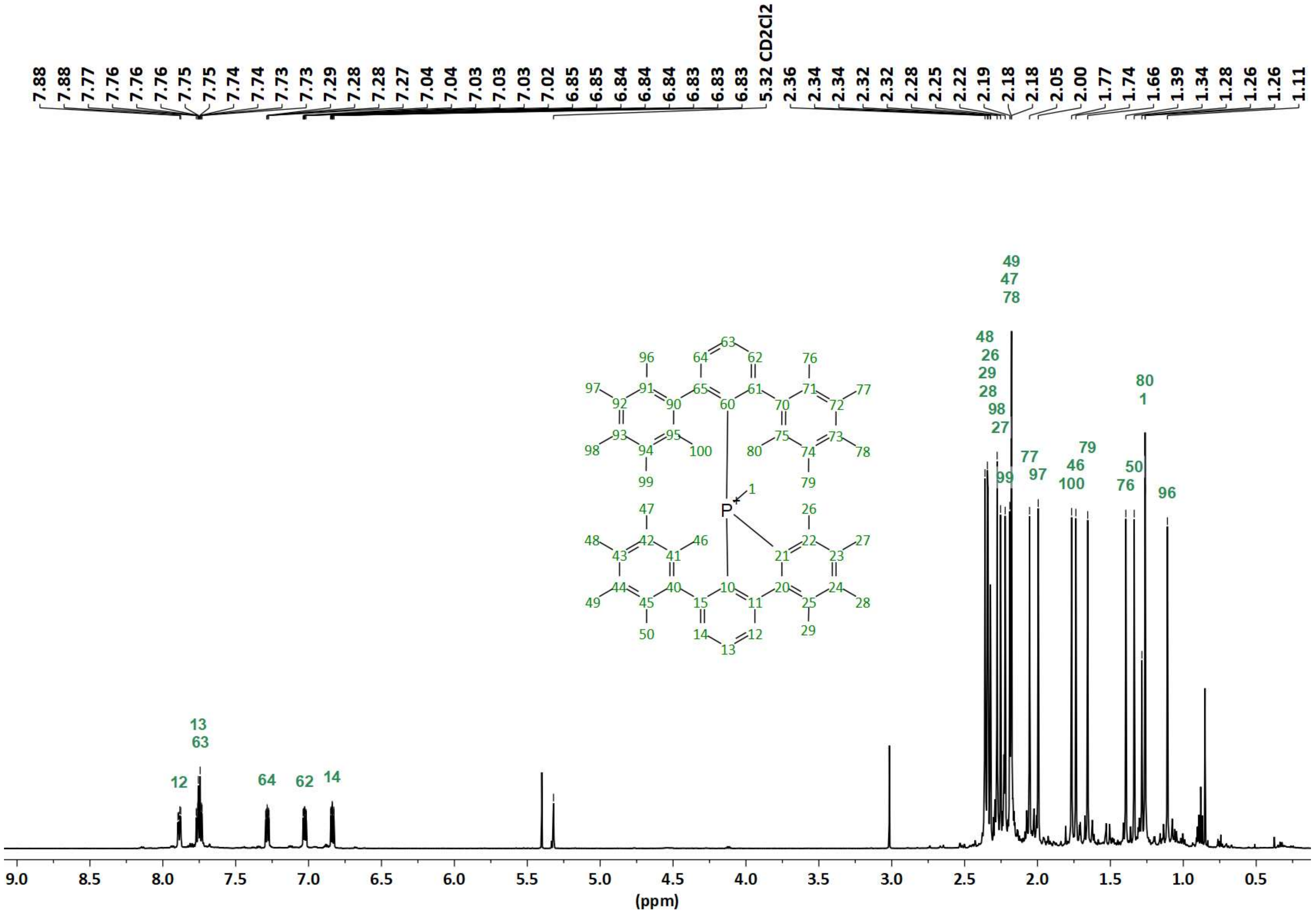

Figure S32. ^{1}H NMR (CD_2Cl_2, 600 MHz) spectrum (full) of [**8a**]$^{+}$.

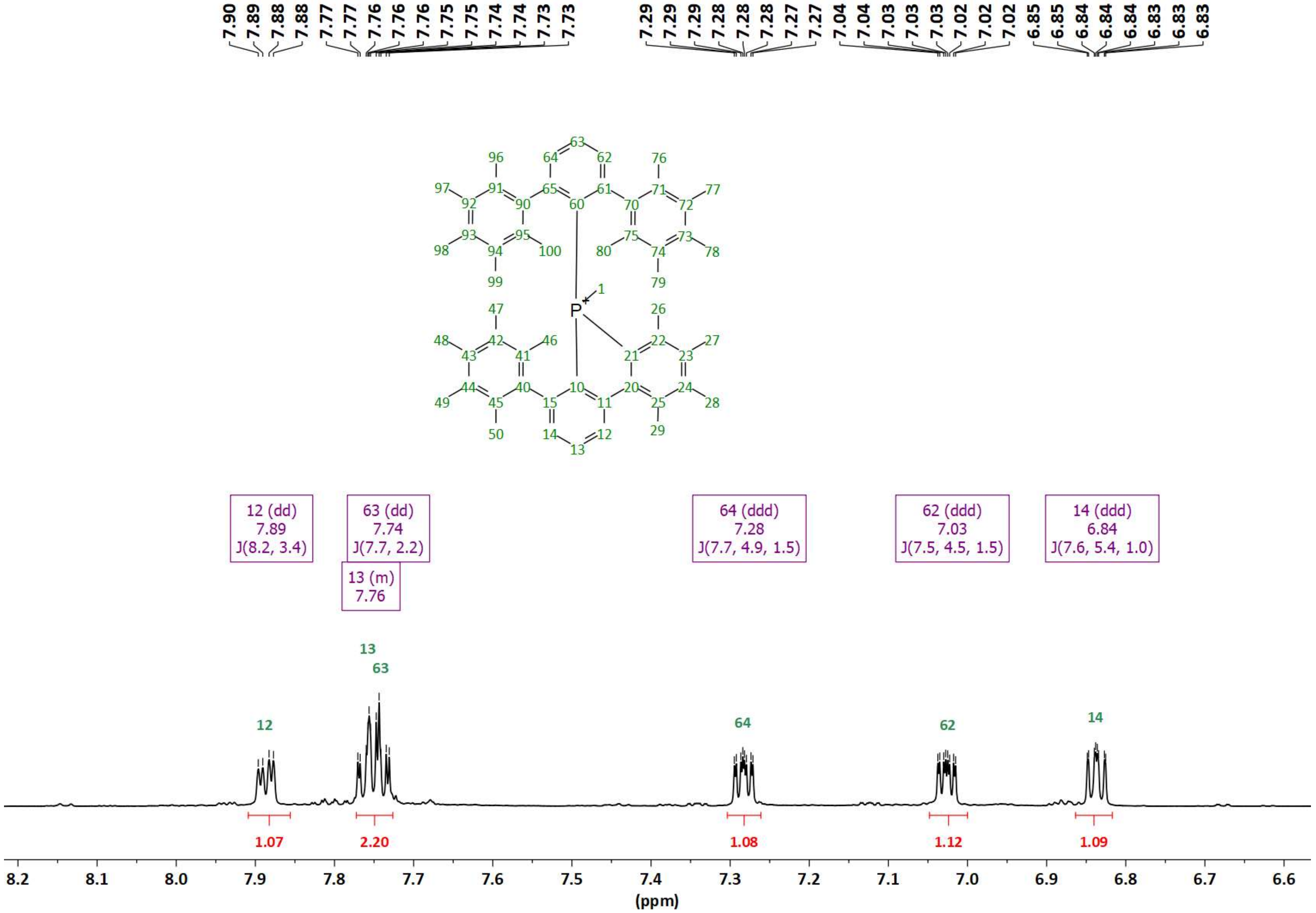

Figure S33. ^{1}H NMR (CD_2Cl_2, 600 MHz) spectrum (detail) of **[8a]**$^{+}$.

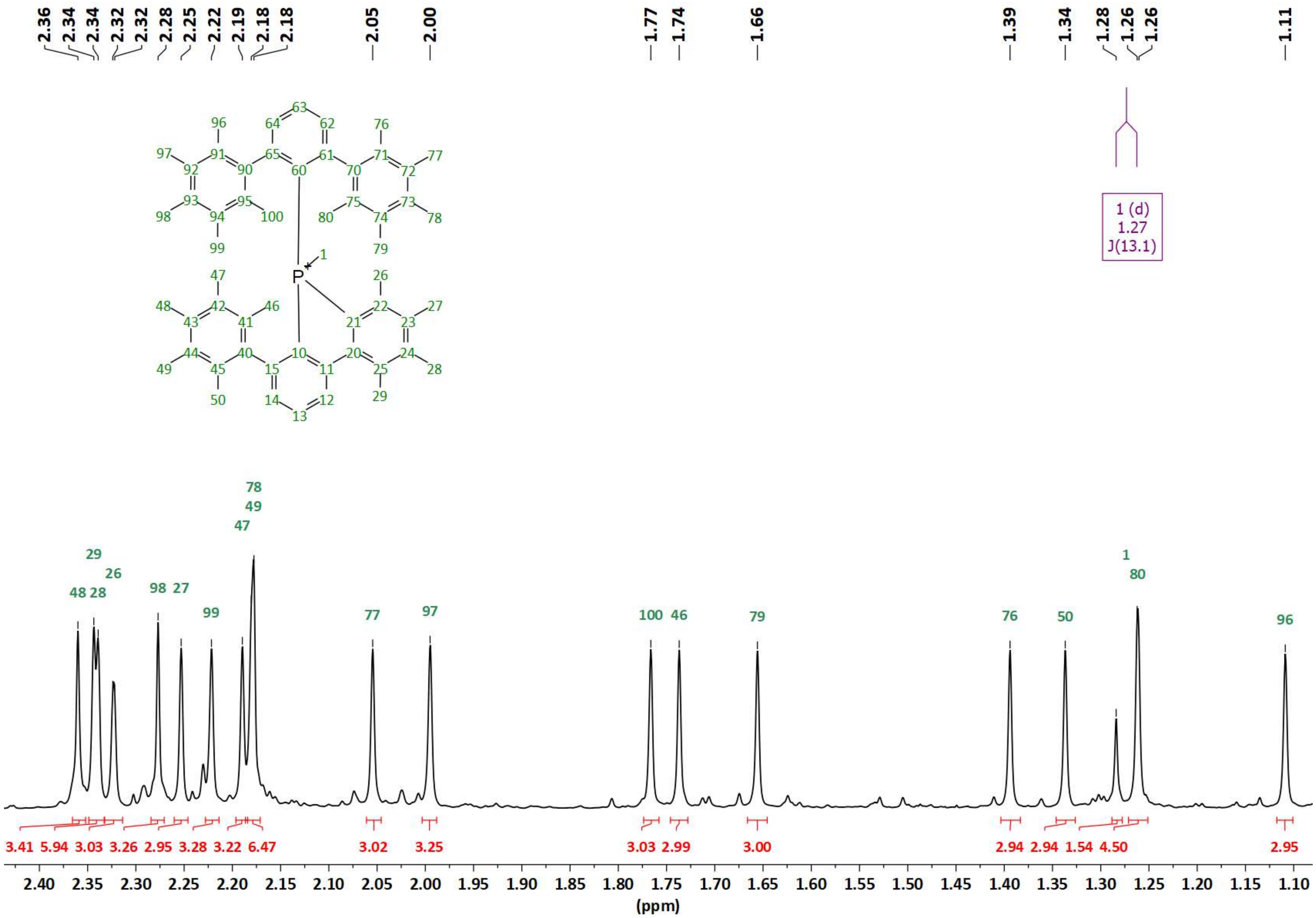

Figure S34. ^{1}H NMR (CD_2Cl_2, 600 MHz) spectrum (detail) of [**8a**]$^{+}$.

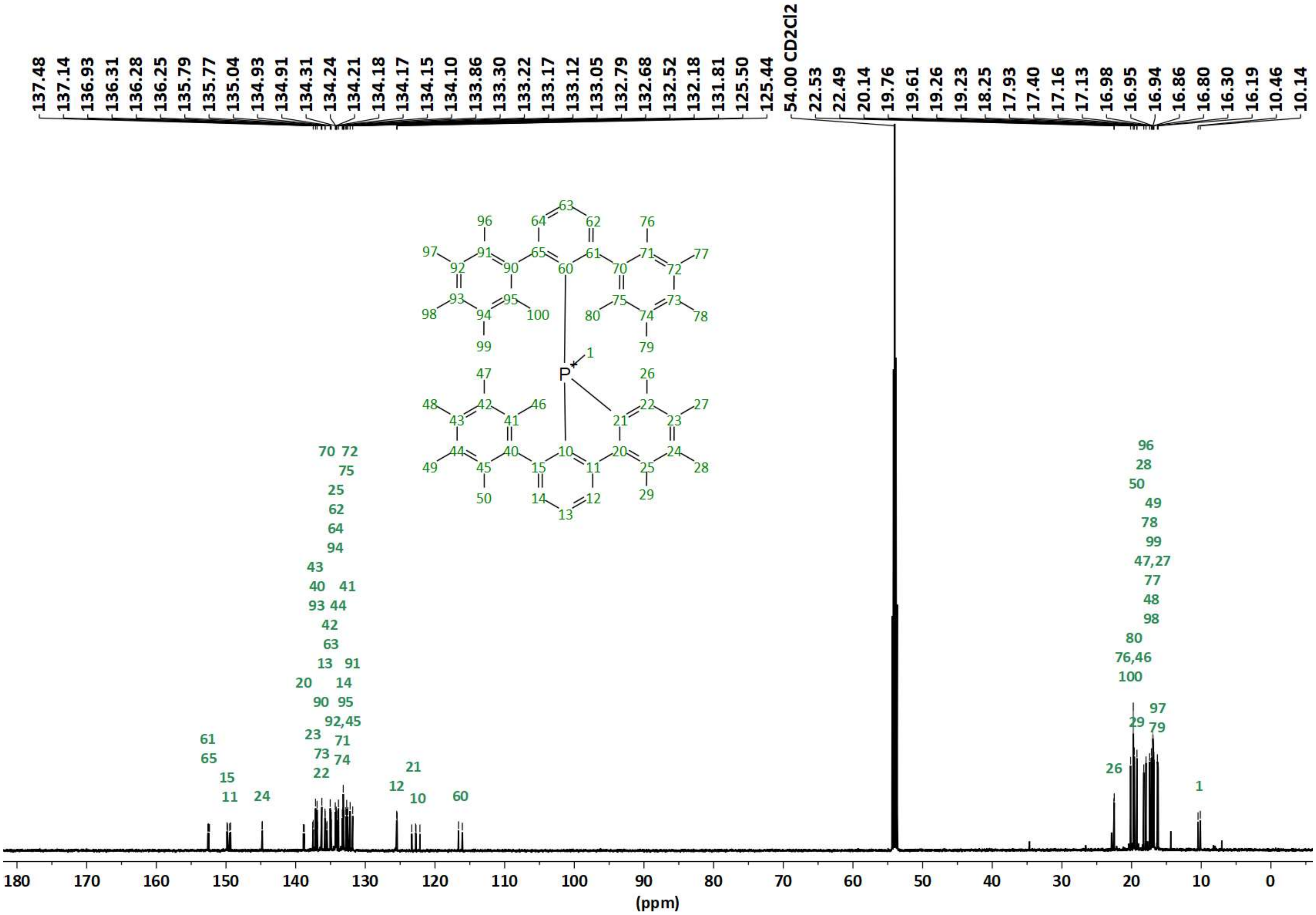

Figure S35. $^{13}C\{^{1}H\}$ NMR (CD_2Cl_2, 151 MHz) spectrum (full) of **[8a]**$^{+}$.

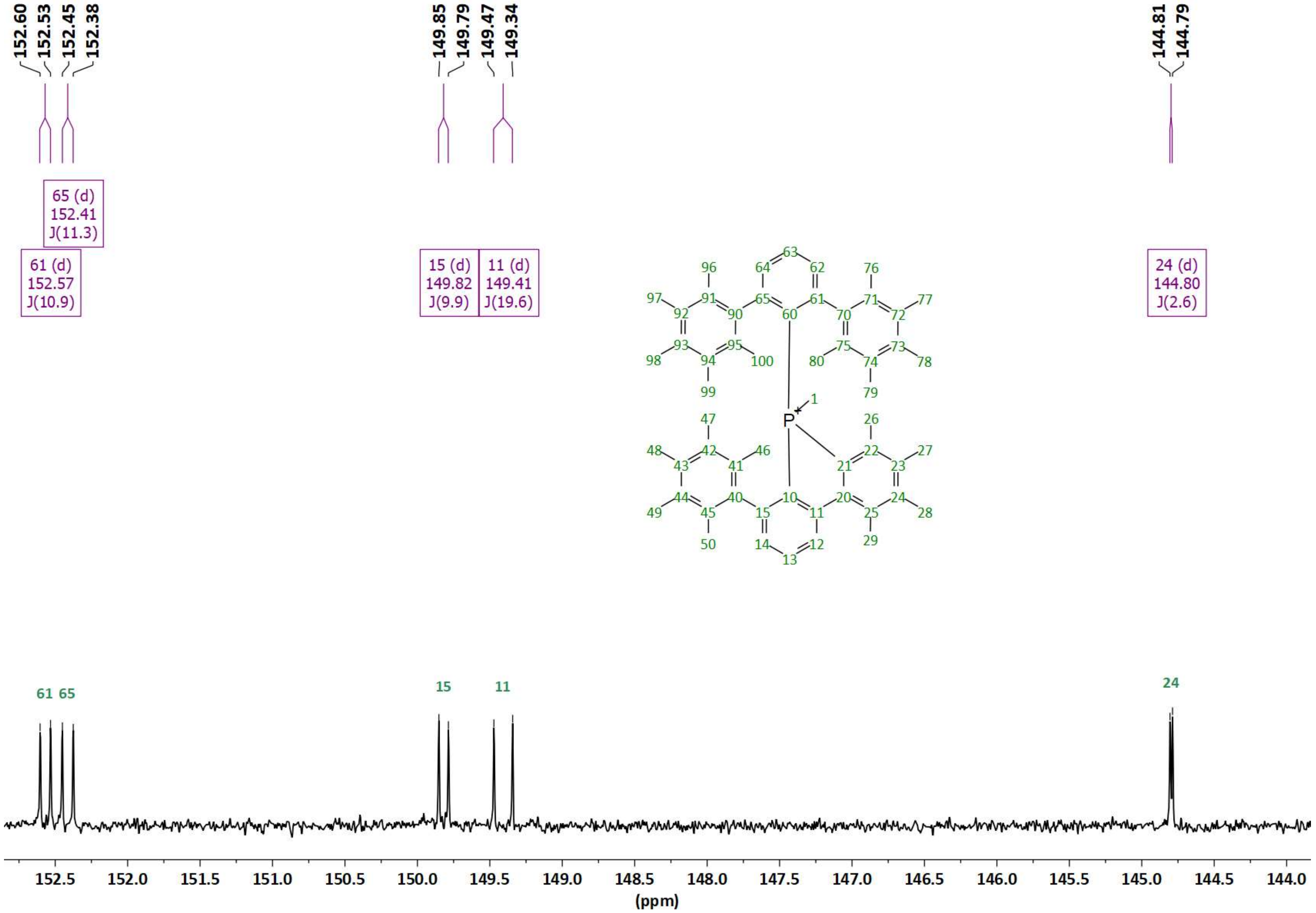

Figure S36. $^{13}C\{^{1}H\}$ NMR (CD_2Cl_2, 151 MHz) spectrum (detail) of [**8a**]$^{+}$.

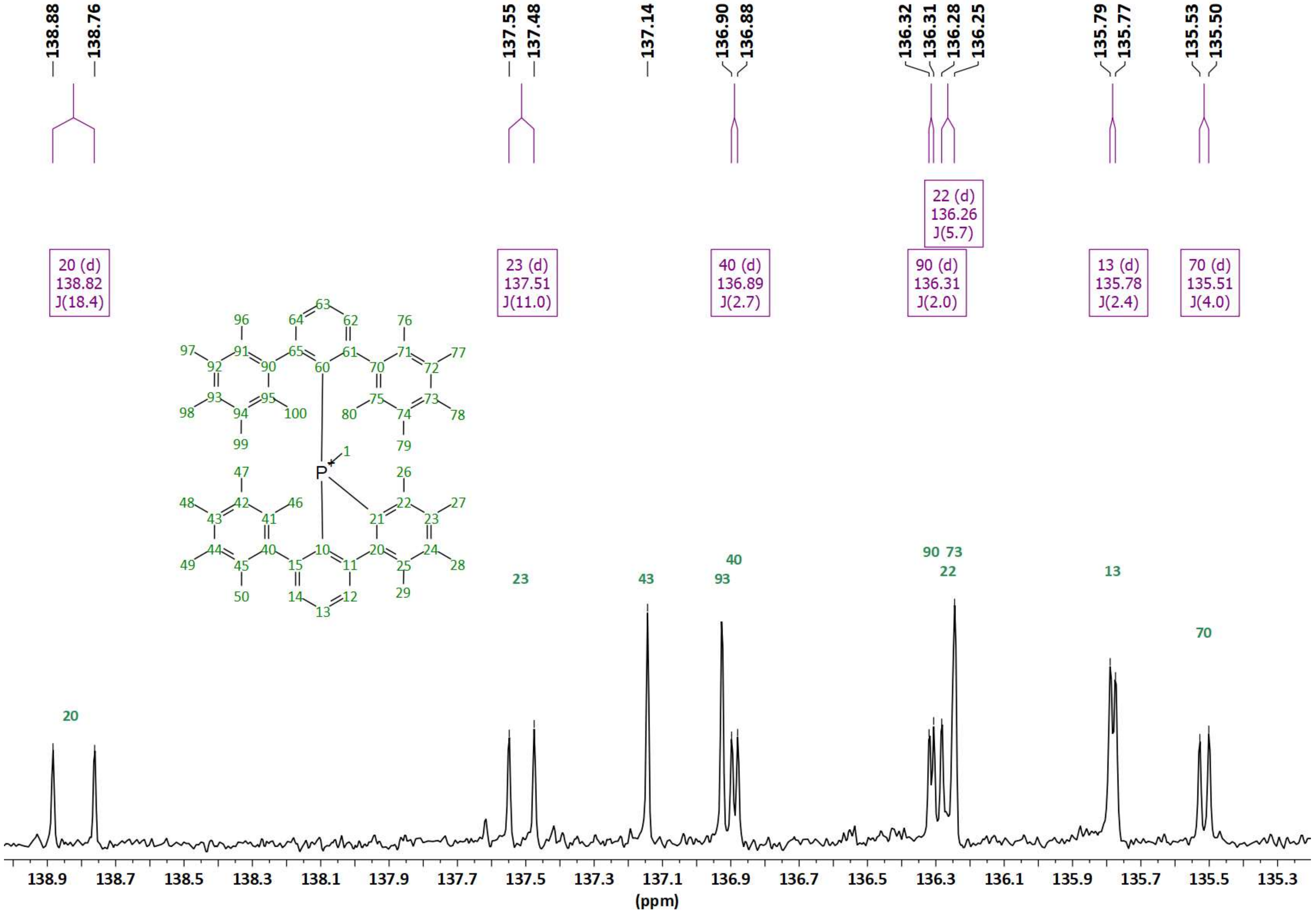

Figure S37. $^{13}C\{^{1}H\}$ NMR (CD_2Cl_2, 151 MHz) spectrum (detail) of [**8a**]$^{+}$.

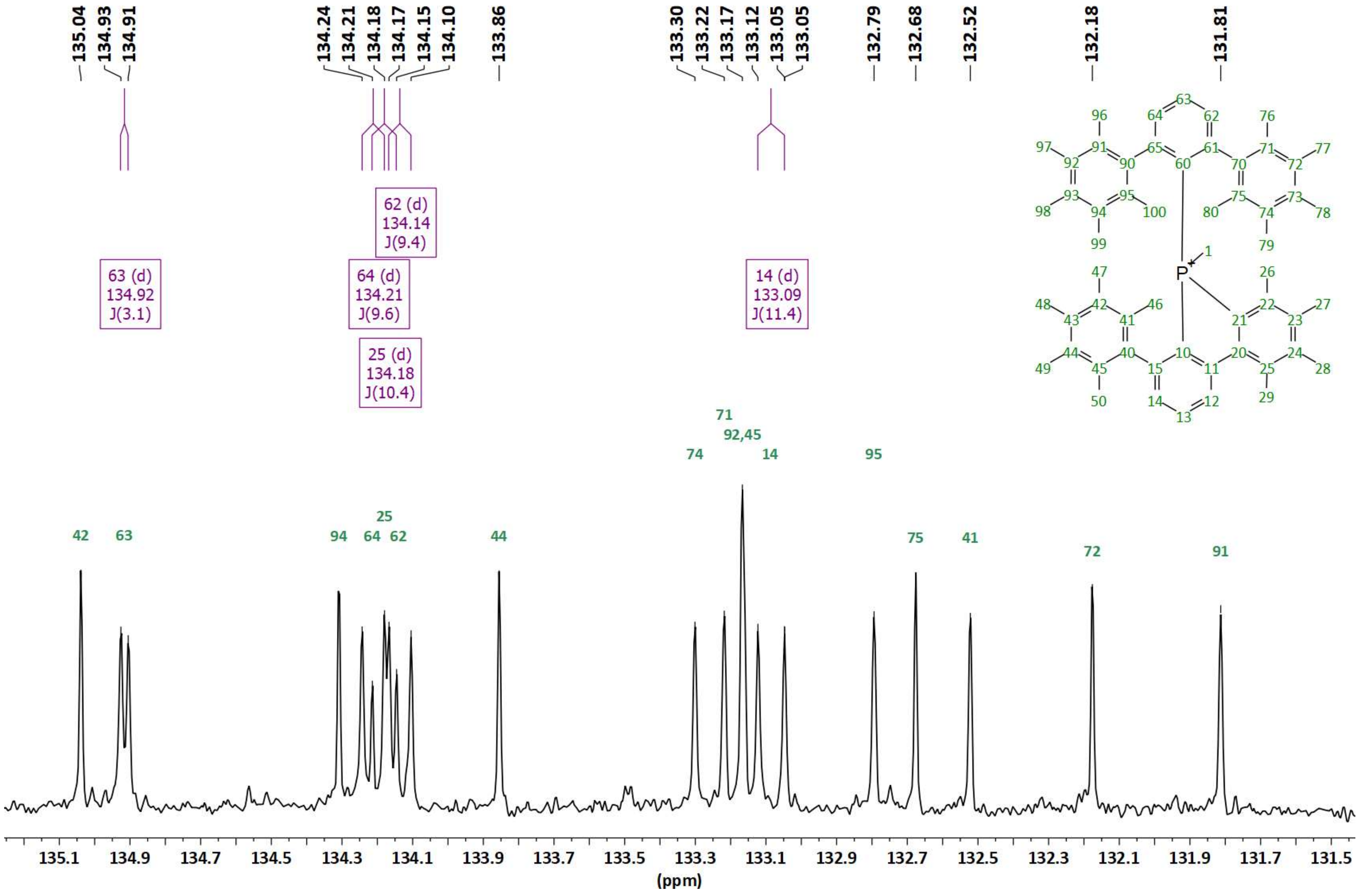

Figure S38. $^{13}C\{^{1}H\}$ NMR (CD_2Cl_2, 151 MHz) spectrum (detail) of [**8a**]$^{+}$.

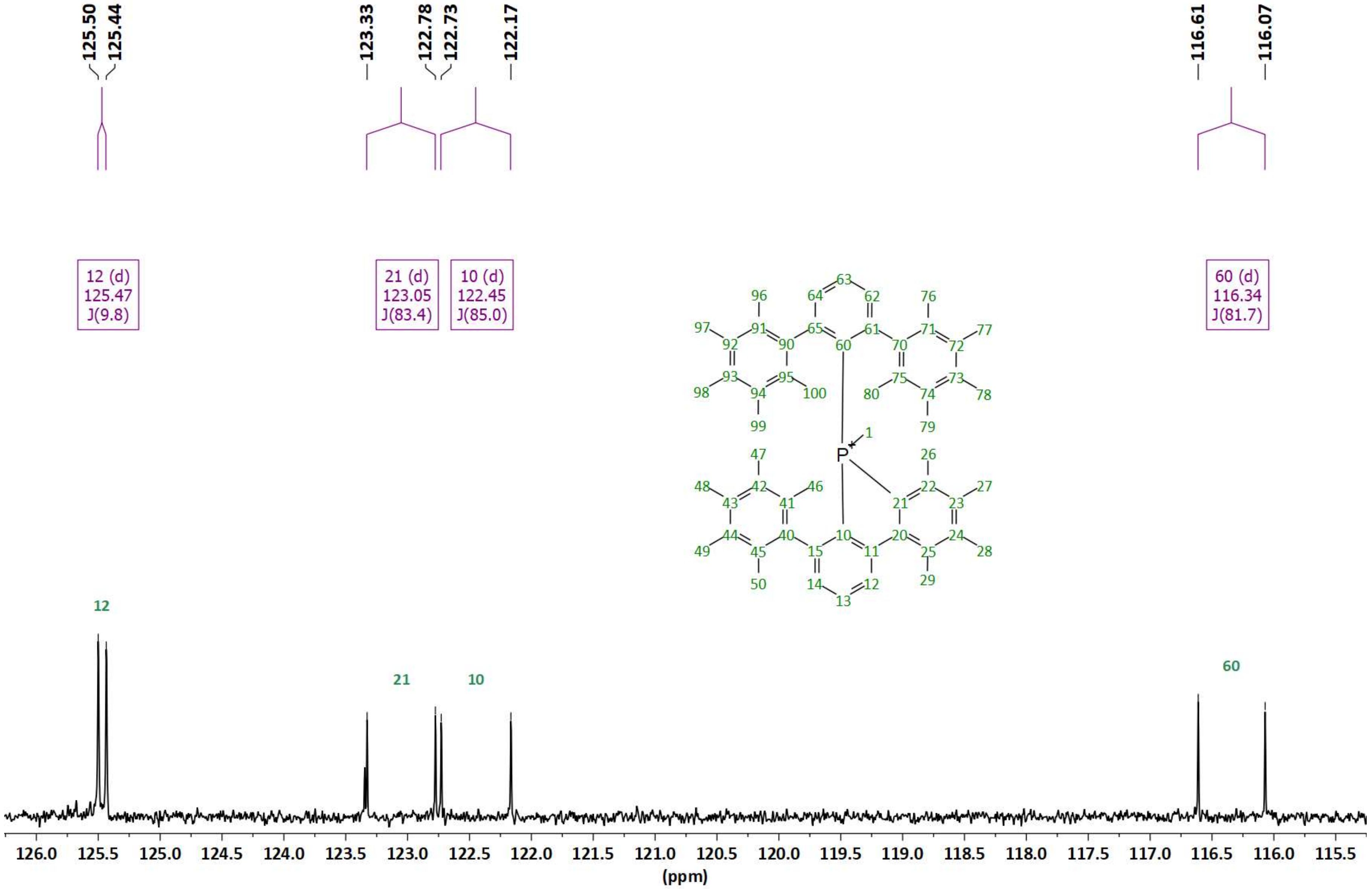

Figure S39. $^{13}C\{^{1}H\}$ NMR (CD_2Cl_2, 151 MHz) spectrum (detail) of [**8a**]$^{+}$.

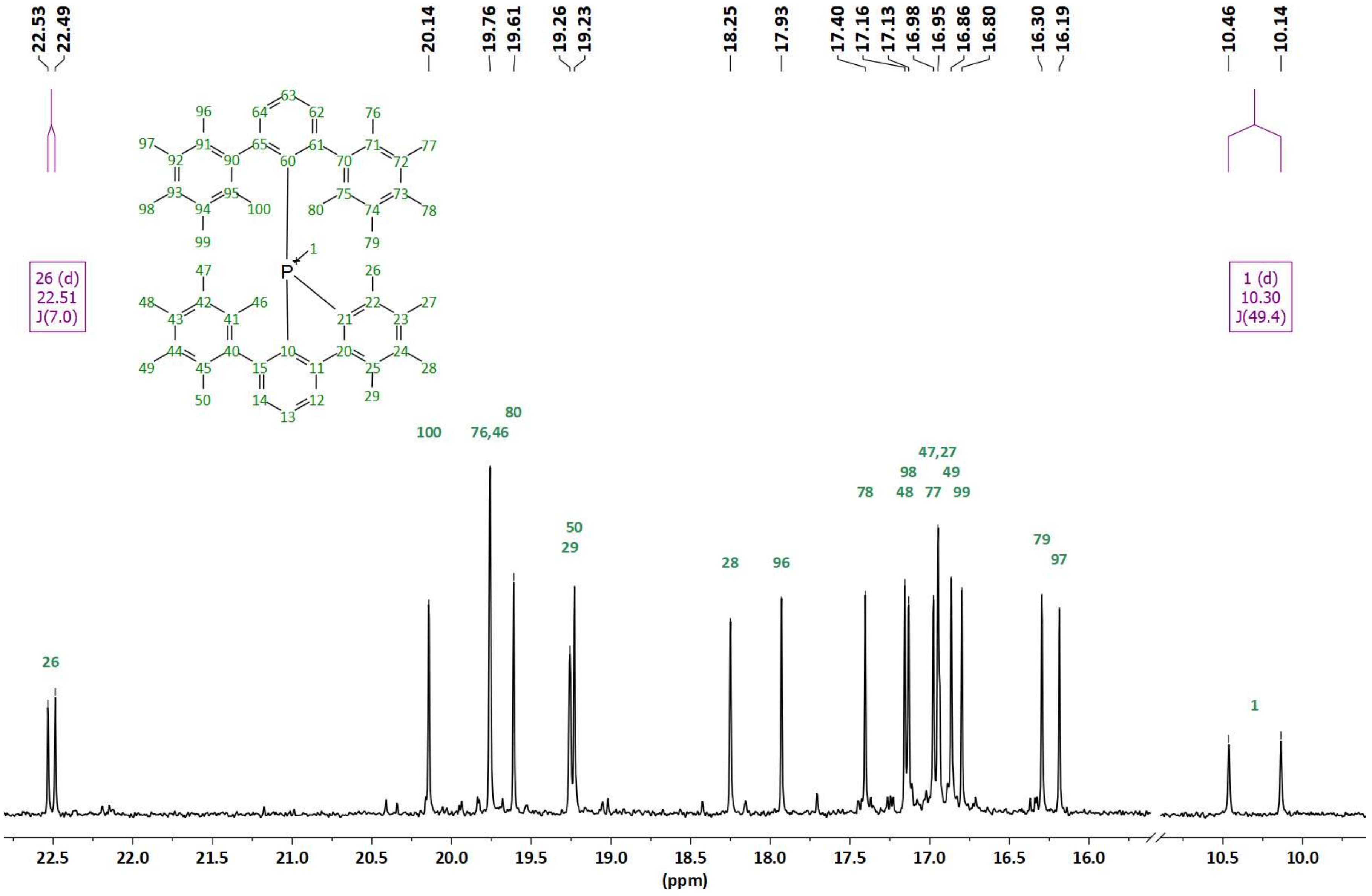

Figure S40. $^{13}C\{^{1}H\}$ NMR (CD_2Cl_2, 151 MHz) spectrum (detail) of [**8a**]$^{+}$.

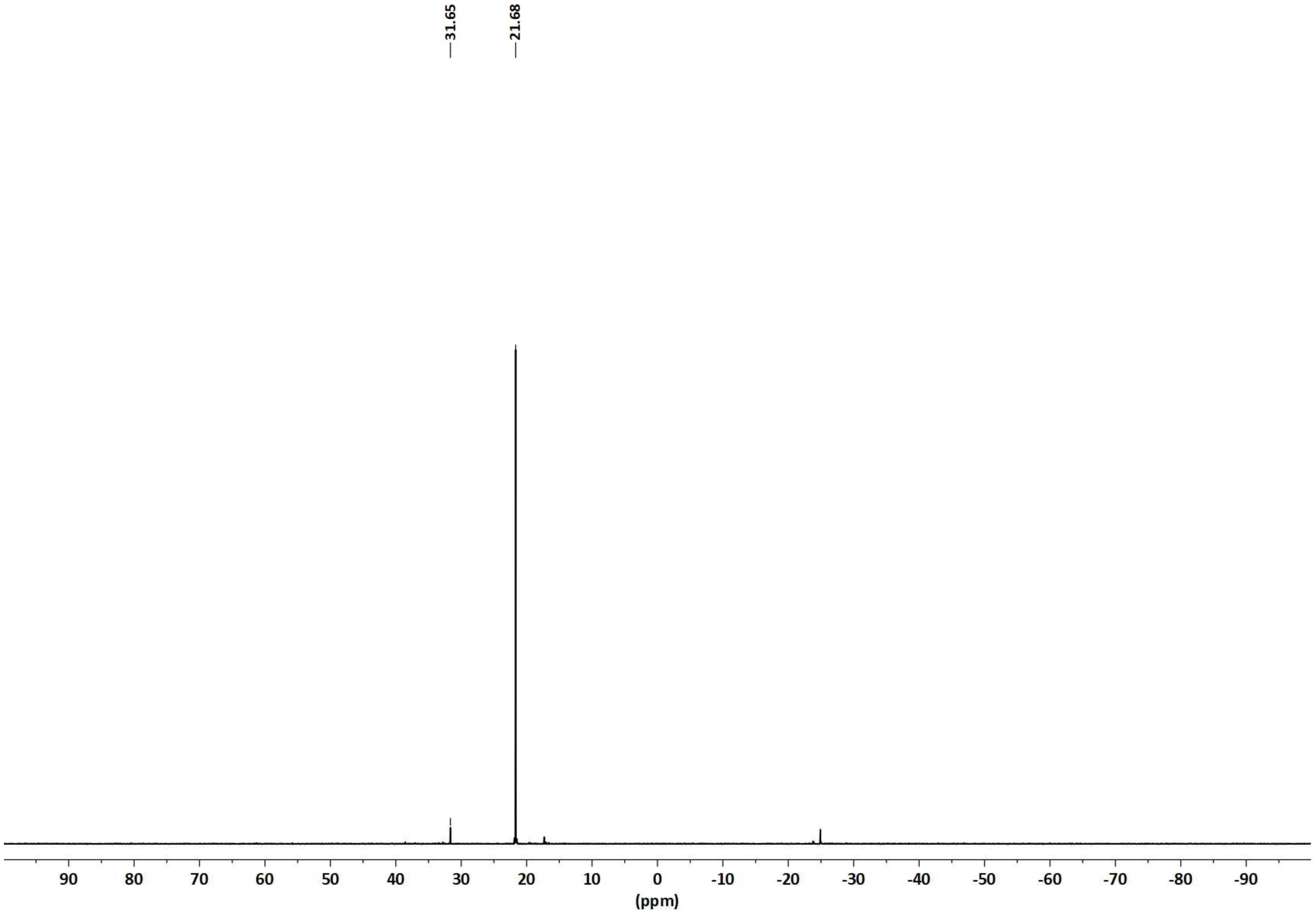

Figure S41. $^{31}P\{^{1}H\}$ NMR (CD_2Cl_2, 243 MHz) spectrum of [**8a**]$^+$. A minor by-product ([**10a**]$^+$) is visible at δ = 31.65 ppm.

Synthesis and characterization of 9a

A J. Young NMR tube was charged with a mixture of [**5a**][$AlCl_4$] and [**7a**][$AlCl_4$] (24 mg) and THF-*d8* (0.5 mL). The solution discoloured to pale red (at higher dilutions it became pale yellow). After NMR spectra were recorded, the solution was diluted with THF (2 mL) and hexane (10 mL) and filtered through a short plug of aluminium oxide. Crystals suitable (16 mg) for single crystal X-ray diffraction were grown by slow evaporation of a solution of **9a** in CH_2Cl_2 and hexane. Neither multiple re-crystallizations nor other attempts (including chromatography) were successful in obtaining **9a** as a pure compound. **^{1}H NMR (600 MHz, THF-*d8*)**: δ = 7.35 (d, $^3J(^1H–^1H)$ = 8 Hz, $^5J(^1H–^{31}P)$ = 1 Hz, 1H, H12), 7.25 (td, $^3J(^1H–^1H)$ = 7 Hz, $^5J(^1H–^{31}P)$ = 1 Hz, 1H, H63), 7.23 (t, $^3J(^1H–^1H)$ = 8 Hz, 1H, H13), 6.88 (ddd, $^3J(^1H–^1H)$ = 8 Hz, $^4J(^1H–^1H)$ = 5 Hz, $^5J(^1H–^{31}P)$ = 2 Hz, 1H, H64), 6.57 (ddd, $^3J(^1H–^1H)$ = 7 Hz, $^4J(^1H–^1H)$ = 5 Hz, $^5J(^1H–^{31}P)$ = 1 Hz, 1H, H14), 6.49 (dd, $^2J(^1H–^1H)$ = 20 Hz, $^4J(^1H–^{31}P)$ = 3 Hz, 1H, H62), 4.79 ($^3J(^1H–^1H)$ = 8 Hz, $^5J(^1H–^{31}P)$ = 2 Hz, 2H, H26), 2.31 (s, 3H, H48), 2.21 (s, 3H, H98), 2.17 (s, 3H, H99), 2.16 (s, 3H, H49), 2.14 (s, 3H, H47), 2.11 (s, 3H, H78), 2.00 (s, 6H, H50, H97), 1.95 (s, 3H, H77), 1.92 (s, 3H, H79), 1.85 (s, 3H, H27), 1.79 (s, 3H, H28), 1.70 (s, 3H, H100), 1.45 (s, 6H, H76, H80), 1.19 (s, 3H, H46), 1.07 (s, 3H, H30), 1.05 (s, 3H, H29), 0.93 (s, 3H, H96) ppm. **^{13}C{^{1}H} NMR (151 MHz, THF-*d8*)**: δ = 155.23 (d, $^2J(^{13}C–^{31}P)$ = 44 Hz, C65), 152.46 (d, $^2J(^{13}C–^{31}P)$ = 7 Hz, C61), 150.92 (d, $^2J(^{13}C–^{31}P)$ = 3 Hz, C20), 149.44 (d, $^2J(^{13}C–^{31}P)$ = 13 Hz, C11), 148.57 (d, $^2J(^{13}C–^{31}P)$ = 21 Hz, C15), 142.91 (d, $^1J(^{13}C–^{31}P)$ = 5 Hz, C10), 142.58 (s, br, C40), 141.78 (d, $^3J(^{13}C–^{31}P)$ = 7 Hz, C90), 141.69 (d, $^2J(^{13}C–^{31}P)$ = 15 Hz, C22), 140.50 (s, C24), 140.30 (d, $^1J(^{13}C–^{31}P)$ = 5 Hz, C21), 139.81 (s, C70), 134.77 (s, C43), 134.54 (s, C71), 173.06 (s, C73), 133.88 (s, C93), 133.58 (s, C41), 133.03 (s, C42), 132.96 (s, br, C44, C95), 132.83 (s, C45), 132.26 (s, C75), 132.04 (s, C74), 132.01 (d, $^1J(^{13}C–^{31}P)$ = 29 Hz, C60), 131.92 (s, C94), 131.81 (s, C62),131.43 (s, C92), 131.40 (s, C91), 131.28 (s, C72), 130.62 (d, $^1J(^{13}C–^{31}P)$ = 9 Hz, C64), 130.22 (d, $^4J(^{13}C–^{31}P)$ = 1 Hz, C63) 129.54 (d, $^3J(^{13}C–^{31}P)$ = 6 Hz, C14), 128.47 (s, C13), 124.59 (d, $^3J(^{13}C–^{31}P)$ = 4 Hz, C23), 123.18 (s, C12), 110.10 (d, $^3J(^{13}C–^{31}P)$ = 18 Hz, C26), 41.12 (s, C25), 28.16 (s, br, C30), 24.76 (d, $^4J(^{13}C–^{31}P)$ = 2 Hz, C29), 20.73 (s, C80), 20.25 (s, C100), 19.87 (s, C50), 19.59 (s, C76), 17.69 (d,$^5J(^{13}C–^{31}P)$ = 4 Hz, C46), 17.61 (d,$^5J(^{13}C–^{31}P)$ = 7 Hz, C96), 17.12 (s, C48), 17.09 (s, C47), 17.08 (s, C78), 16.95 (s, C98), 16.84 (s, C49), 16.76 (s, C99), 16.74 (s, C77), 16.44 (s, C79), 16.42 (s, C97), 15.75 (s, C28), 14.98 (d, $^4J(^{13}C–^{31}P)$ = 3 Hz, C27) ppm. **^{31}P{1H} NMR (243 MHz, THF-*d8*)**: δ = −24.23 (s) ppm. **HRMS ESI (m/z):** $[M−H]^+$ calculated. for $C_{56}H_{64}P$,767.47402; found, 767.47264.

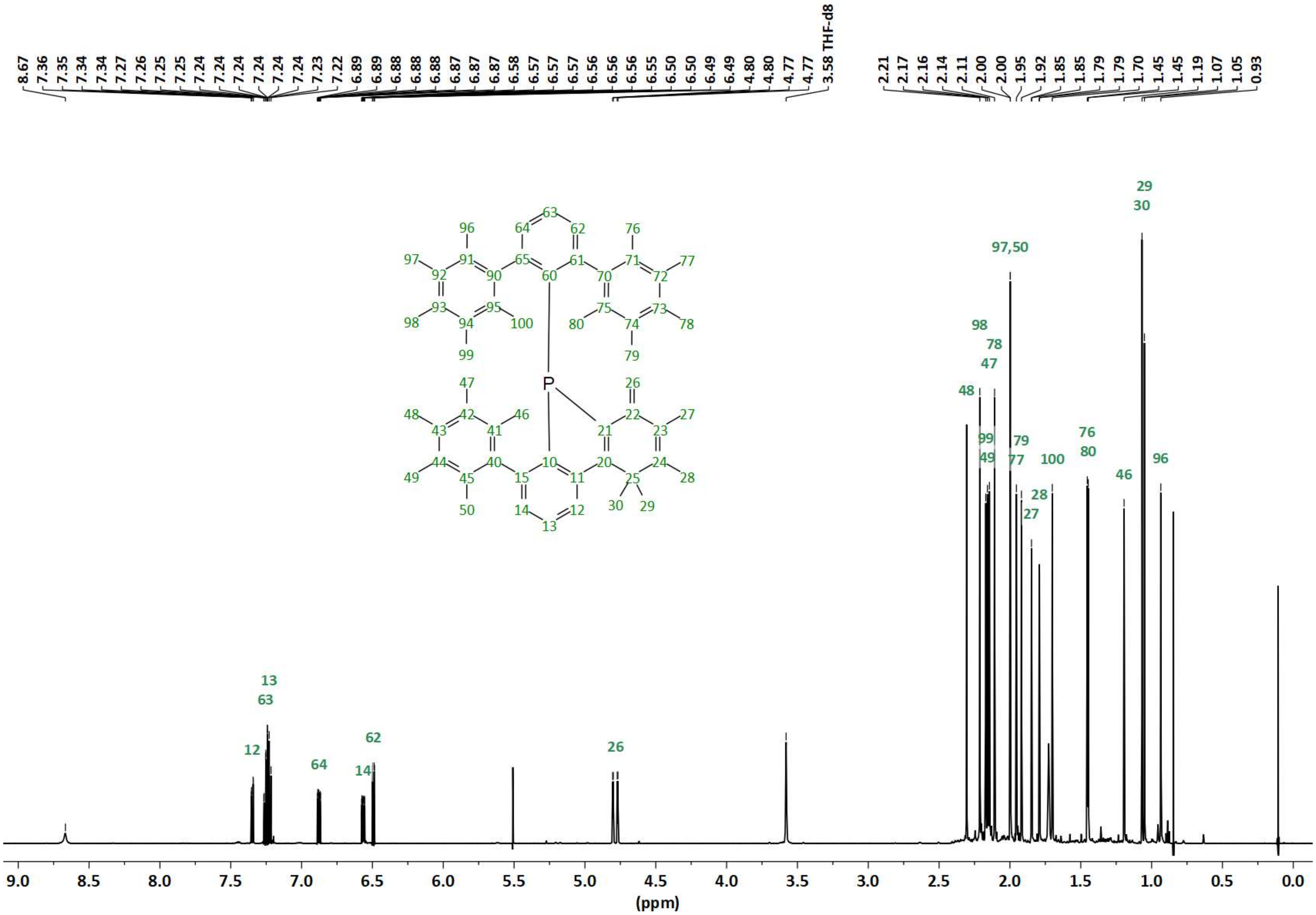

Figure S42. ^{1}H NMR (CD_2Cl_2, 600 MHz) spectrum (full) of **9a**.

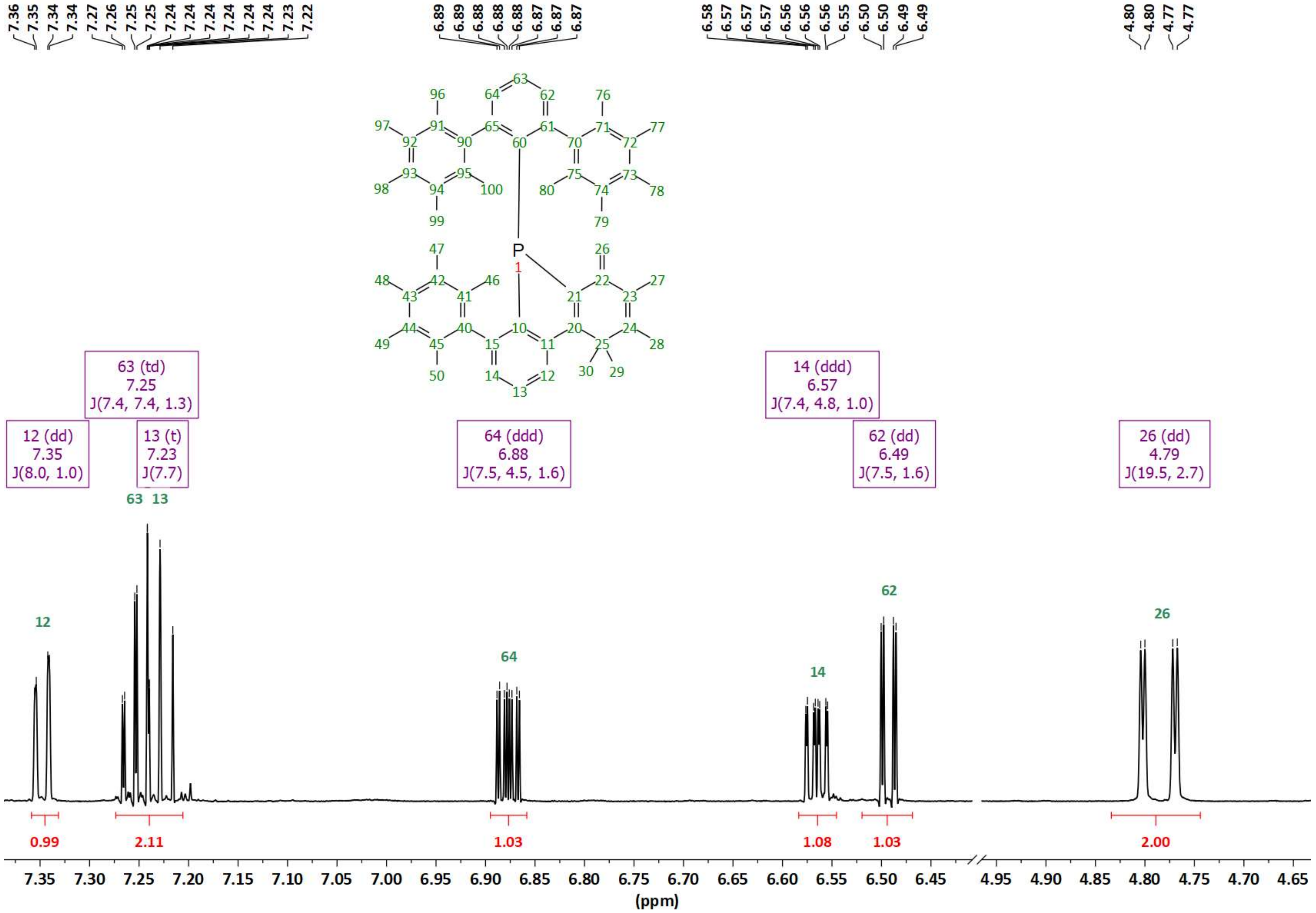

Figure S43. ^{1}H NMR (CD_2Cl_2, 600 MHz) spectrum (detail) of **9a**.

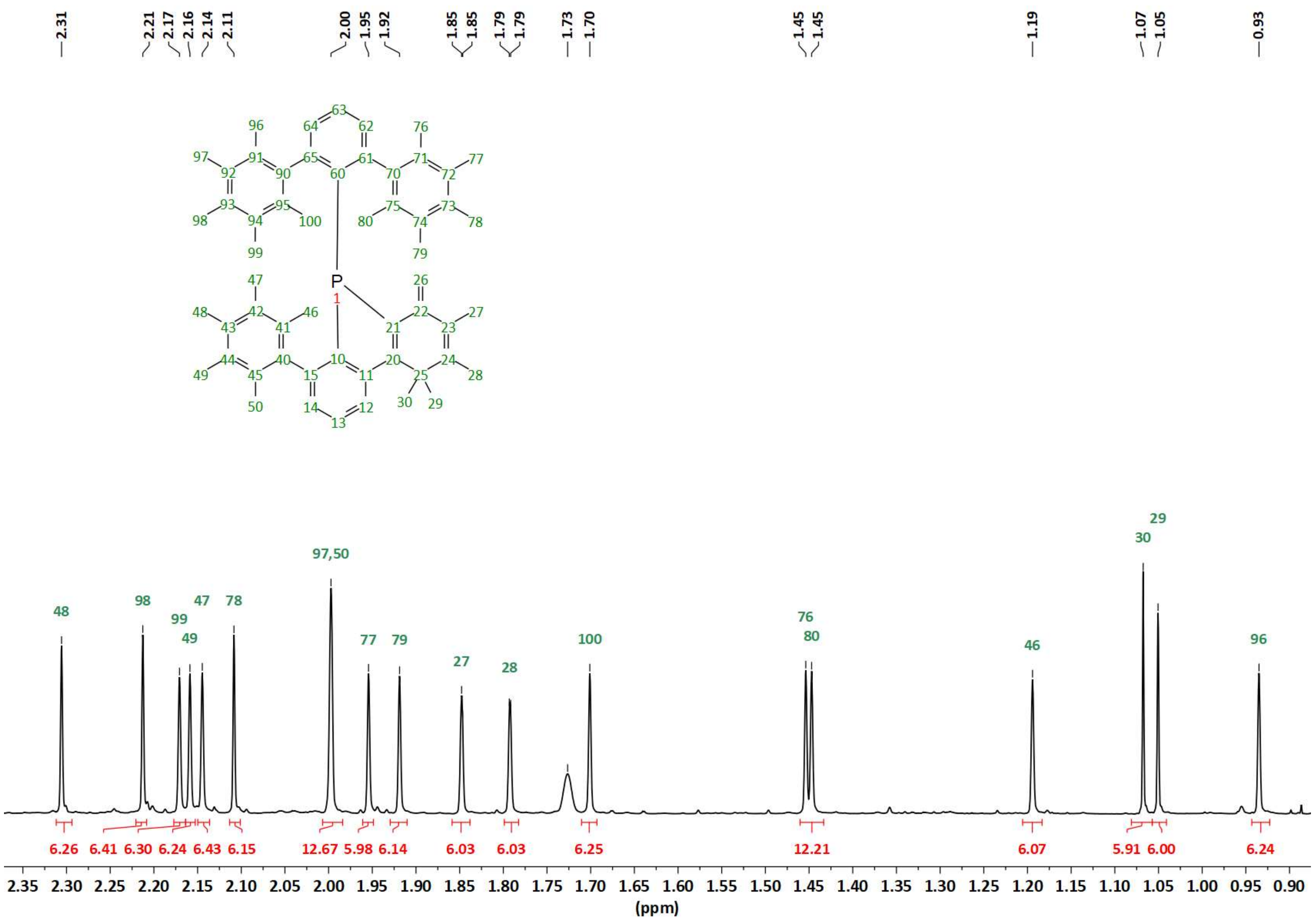

Figure S44. ^{1}H NMR (CD_2Cl_2, 600 MHz) spectrum (detail) of **9a**.

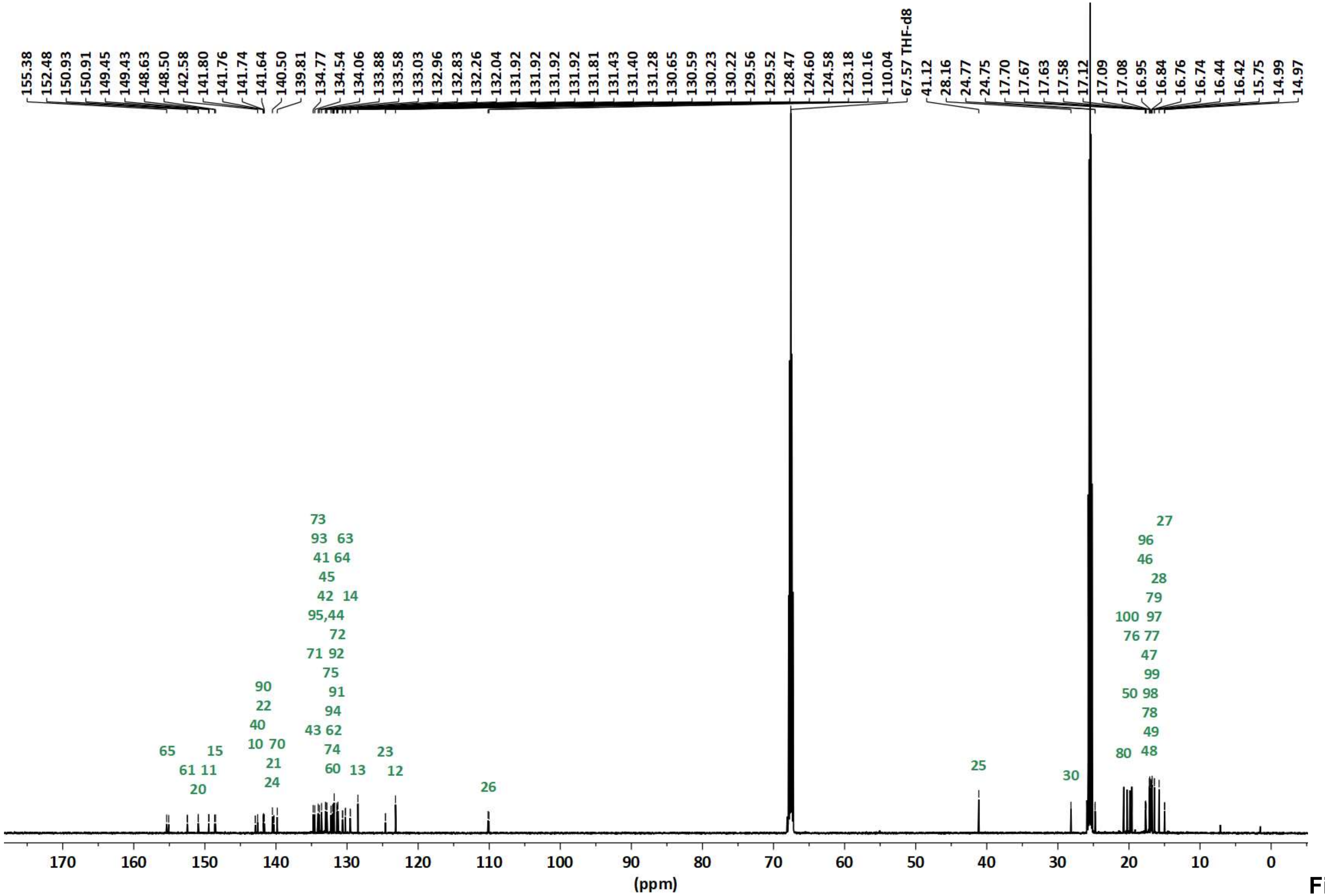

Figure

Figure S45. $^{13}C\{^1H\}$ NMR (CD_2Cl_2, 151 MHz) spectrum (full) of **9a**.

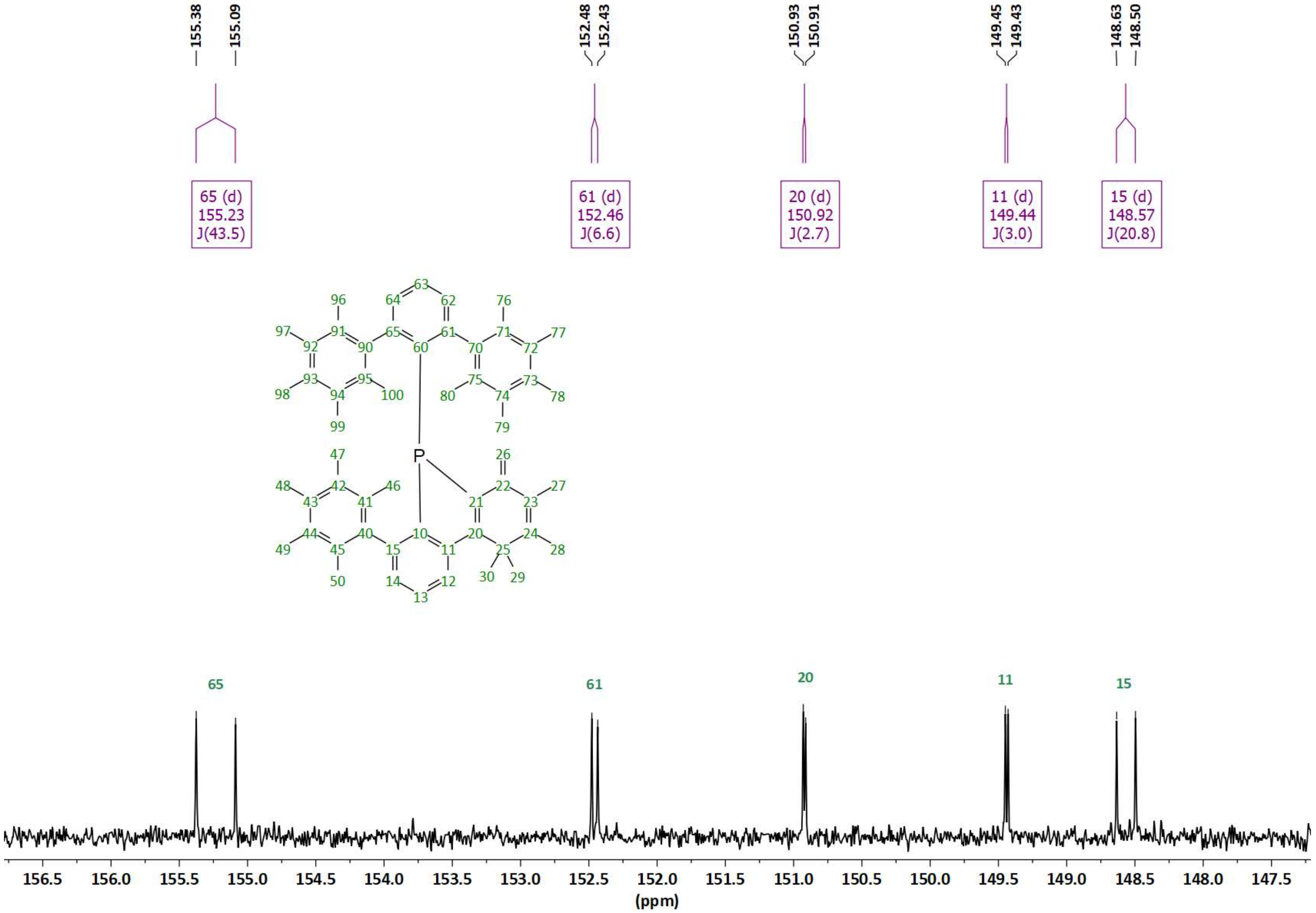

Figure S46. $^{13}C\{^{1}H\}$ NMR (CD_2Cl_2, 151 MHz) spectrum (detail) of **9a**.

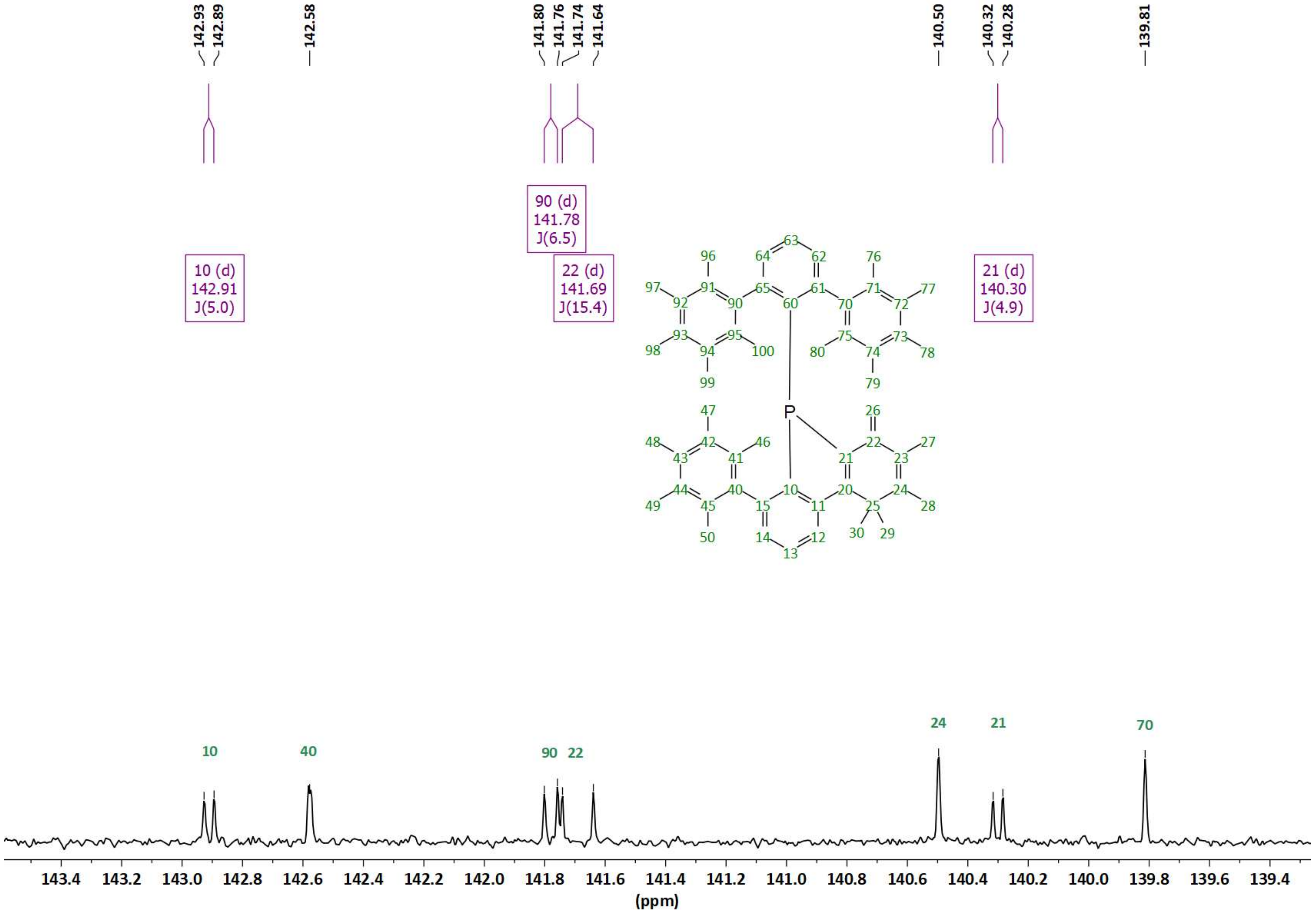

Figure S47. $^{13}C\{^1H\}$ NMR (CD_2Cl_2, 151 MHz) spectrum (detail) of **9a**.

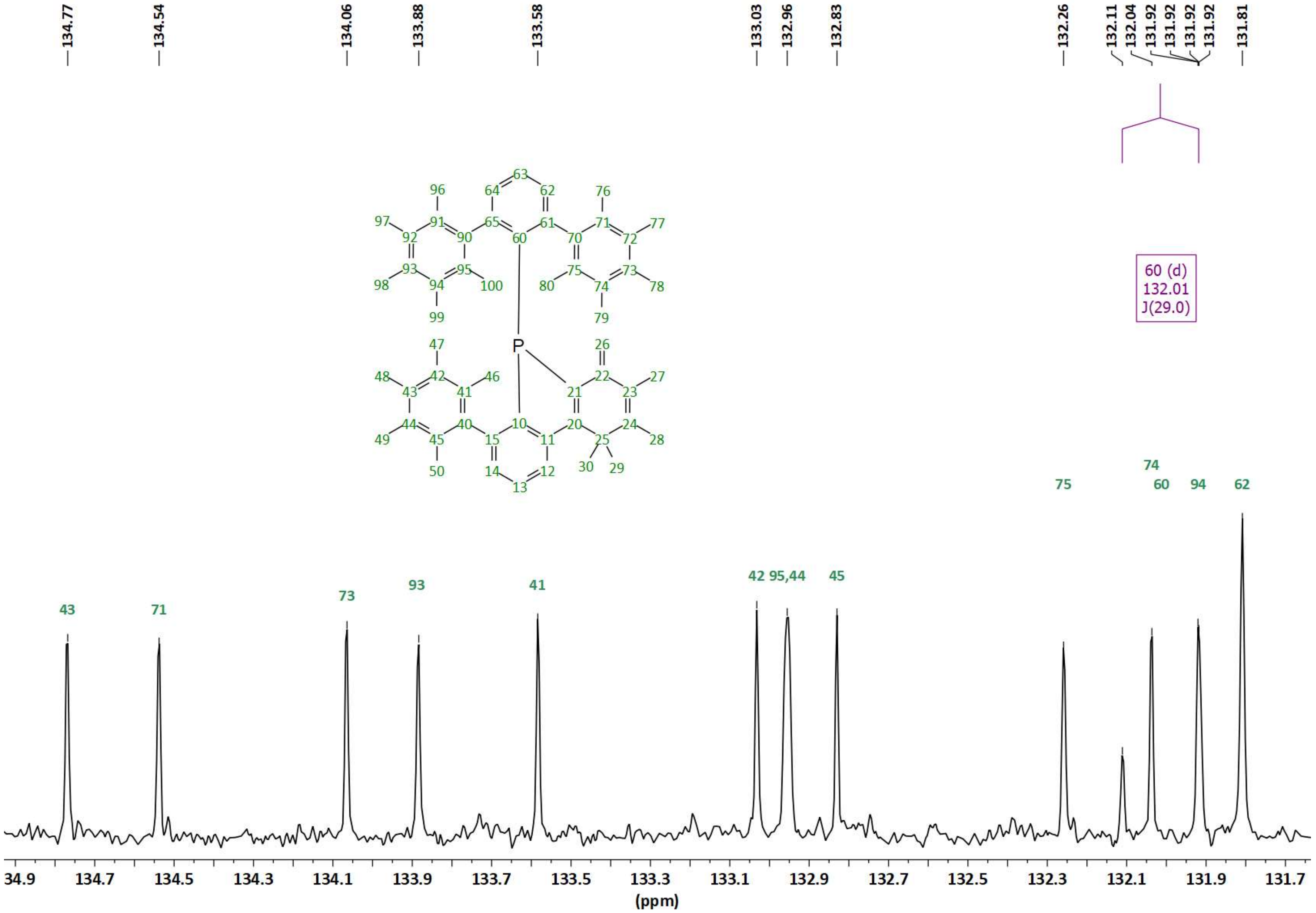

Figure S48. $^{13}C\{^1H\}$ NMR (CD_2Cl_2, 151 MHz) spectrum (detail) of **9a**.

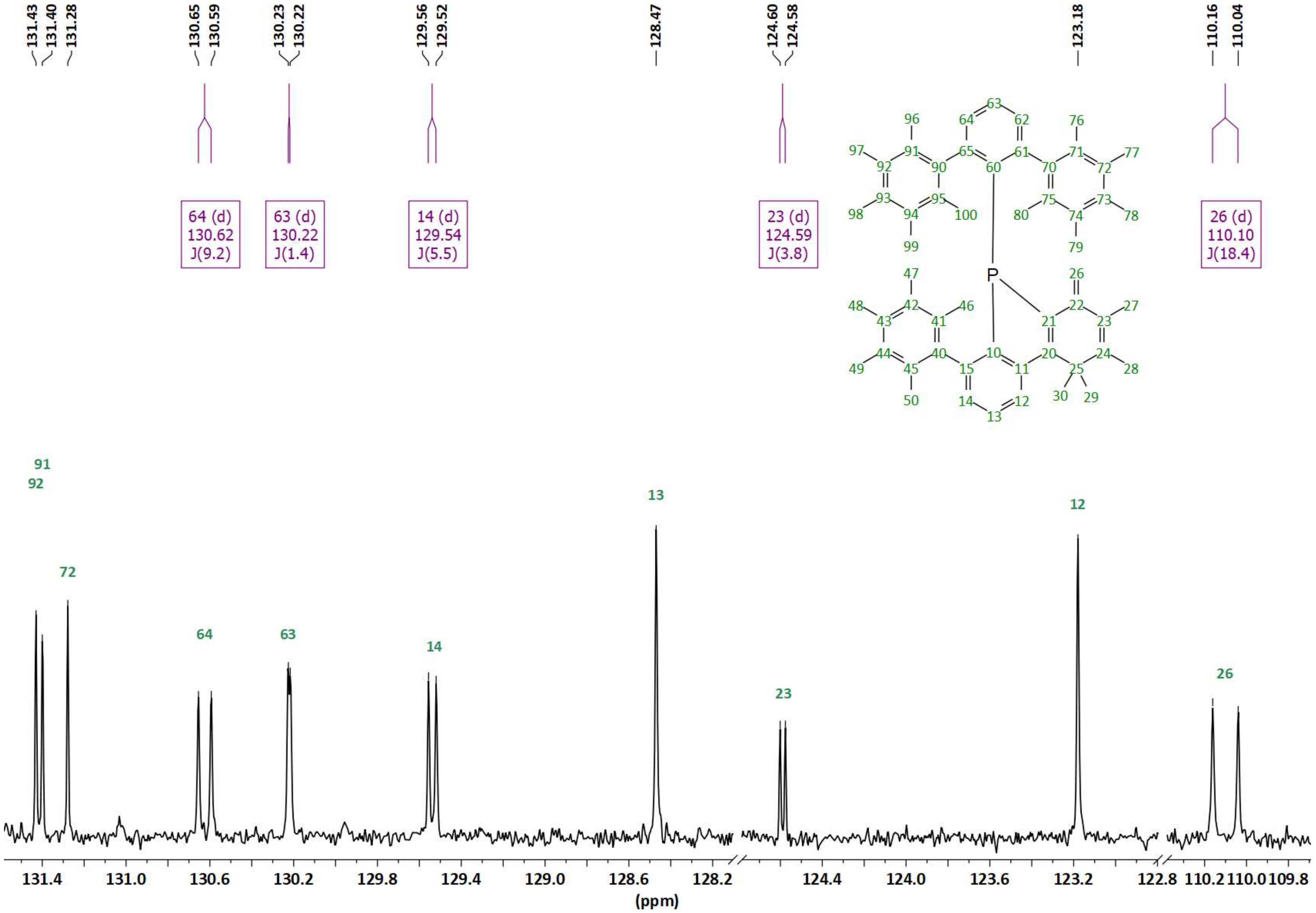

Figure S49. $^{13}C\{^{1}H\}$ NMR (CD_2Cl_2, 151 MHz) spectrum (detail) of **9a**.

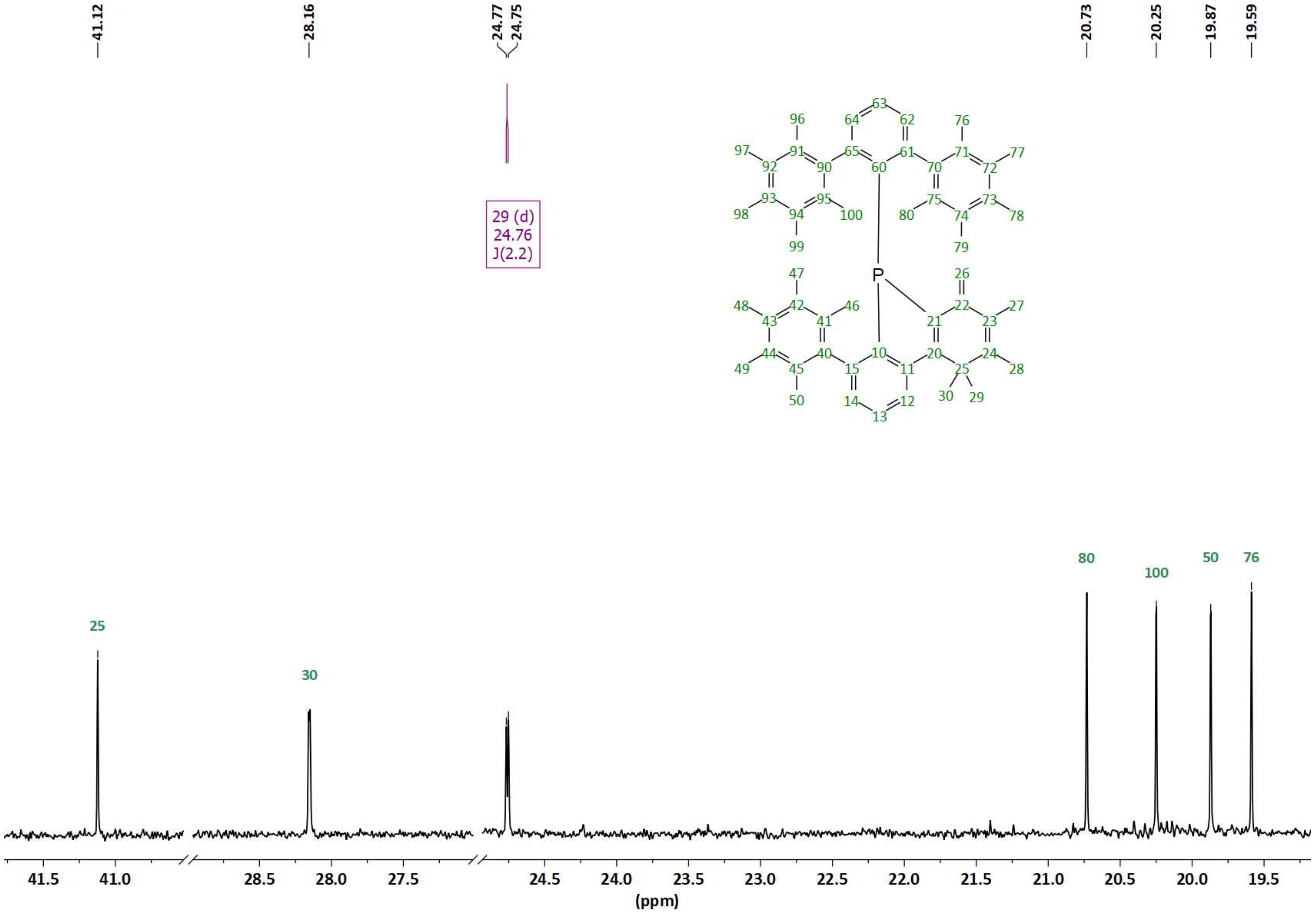

Figure S50. $^{13}C\{^{1}H\}$ NMR (CD_2Cl_2, 151 MHz) spectrum (detail) of **9a**.

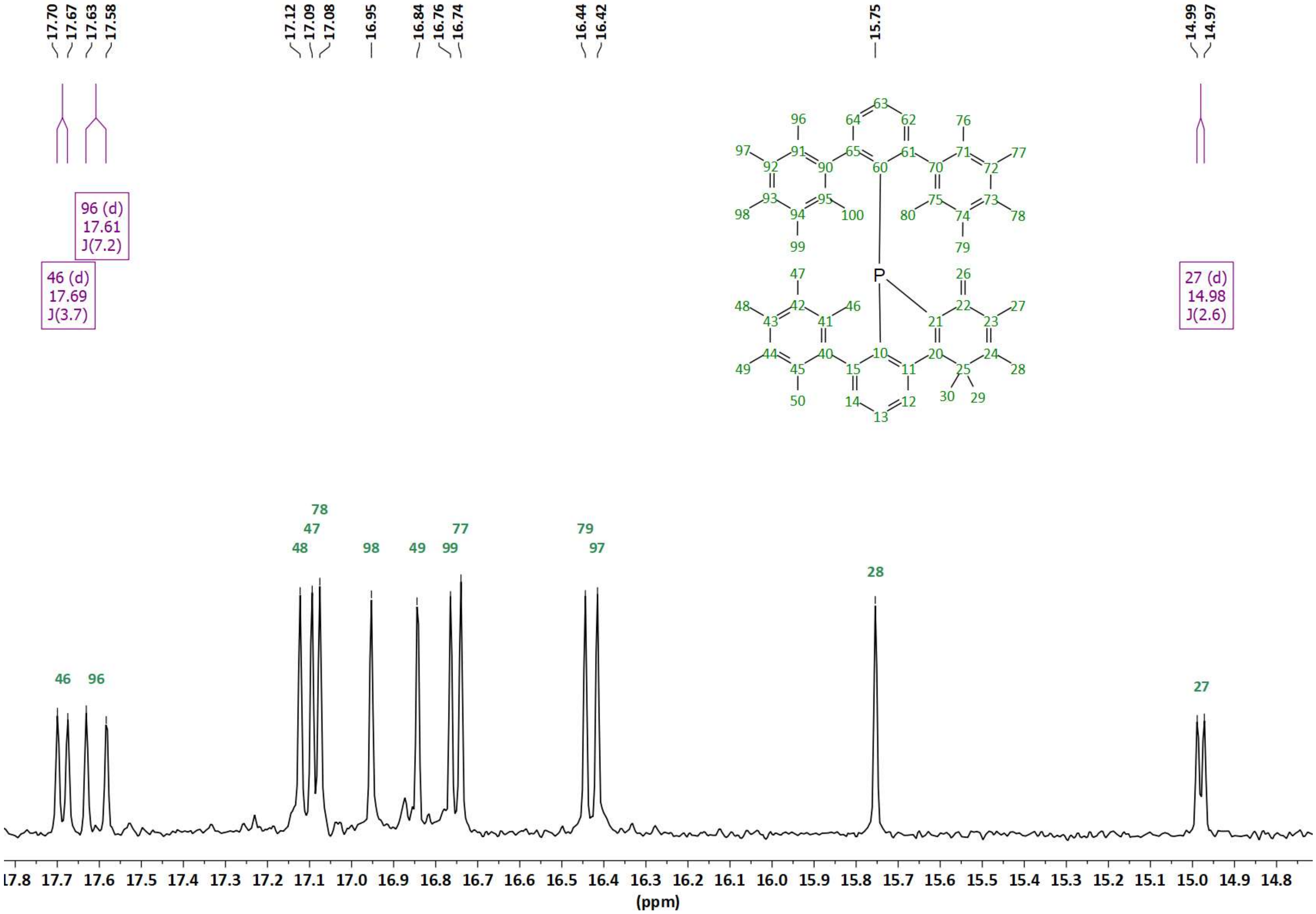

Figure S51. $^{13}C\{^{1}H\}$ NMR (CD_2Cl_2, 151 MHz) spectrum (detail) of **9a**.

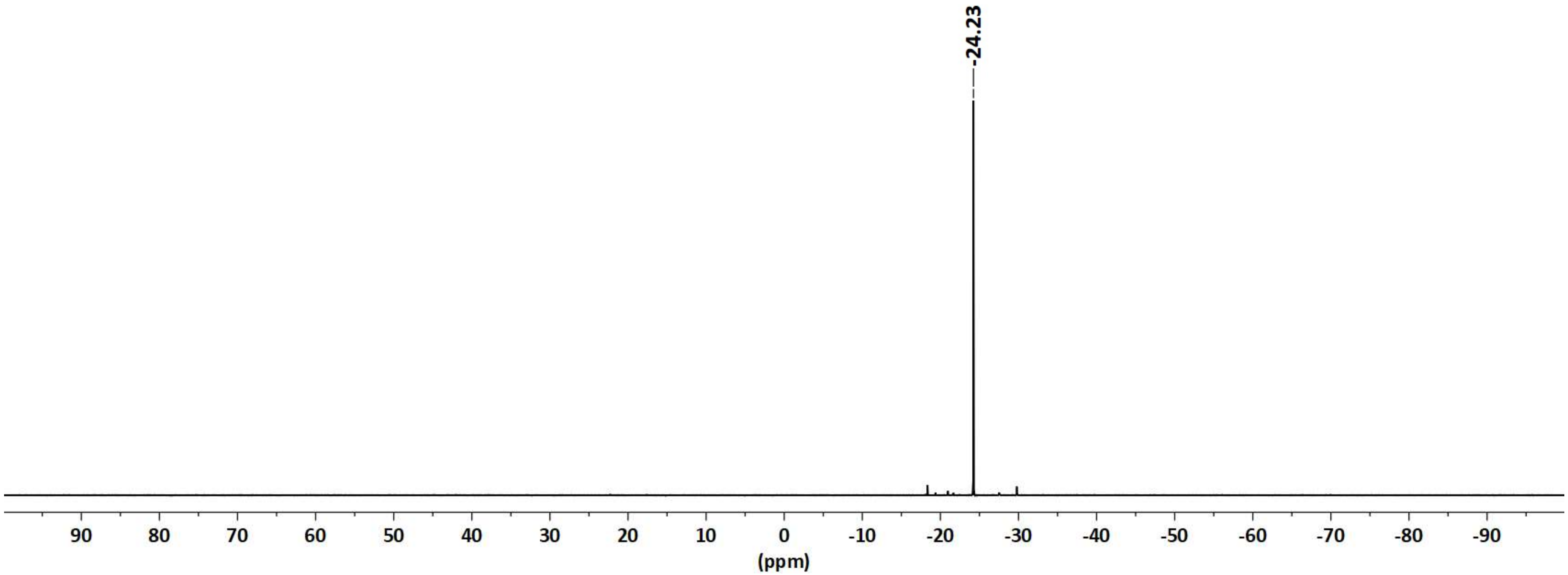

Figure S52. $^{31}P\{^{1}H\}$ NMR (CD_2Cl_2, 243 MHz) spectrum of **9a**.

Synthesis and characterization of 9b

A mixture of [**5b**][$AlCl_4$] and [**7b**][$AlCl_4$] (50 mg, 0.05 mmol) was added to a Schlenk tube and to this dry THF (4 mL) was added. The mixture was stirred for 1h at room temperature and then layered with hexane (40 mL). After diffusion the remaining solution was filtered and washed with water (3×40 mL). The solution was slowly evaporated to yield **9b** as pale orange crystalline solid (27 mg, 65%). Crystals suitable for X-ray diffraction measurement were grown by evaporation of a heptane solution. **Mp** 285–290 °C (decomp.). **^{1}H-NMR (600 MHz, THF-*d8*)**: δ = 7.29 (d, $^3J(^1H–^1H)$ = 7.60 Hz, 1H, H12), 7.24 (t, $^3J(^1H–^1H)$ = 7.60 Hz, 1H, H13), 7.23 (t, $^3J(^1H–^1H)$ = 7.50 Hz, 1H, H63), 6.85 (dd, $^3J(^1H–^1H)$ = 7.50 Hz, $^4J(^1H–^1H)$ = 1.50 Hz, 1H, H64), 6.63 (d, $^3J(^1H–^1H)$ = 7.60 Hz, 1H, H14), 6.46 (dd, $^3J(^1H–^1H)$ = 7.50 Hz, $^4J(^1H–^1H)$ = 1.50 Hz, 1H, H62), 4.84(s, 1H, H26), 4.61 (s, 1H, H26), 2.33 (s, 3H, H48), 2.27 (s, 3H, H98), 2.21 (s, 3H, H78), 2.18 (s, 3H, H47), 2.15 (s, 3H, H49), 2.11 (s, 3H, H77), 2.05 (s, 3H, H46), 2.00 (s, 3H, H99), 1.95 (s, 3H, H79), 1.91 (s, 3H, H97), 1.83 (s, 3H, H27), 1.79 (s, 3H, H28), 1.76 (s, 3H, H96), 1.46 (s, 3H, H80), 1.42 (s, 3H, H76), 1.31 (s, 3H, H50), 1.12 (s, 3H, H29), 1.03 (s, 3H, H30), 0.92 (s, 3H, H100) ppm. **^{13}C{^{1}H}-NMR (151 MHz, THF-*d8*)**: δ = 152.56 (s, C65), 152.47 (s, C20), 151.29 (s, C61), 150.83 (s, C11), 148.05 (s, C15), 145.64 (s, C10), 143.95 (s, C21), 142.41 (s, C22), 142.01 (s, C40), 141.73 (s, C90), 141.42 (s, C24), 139.41 (s, C70), 136.08 (s, C60), 134.70 (s, C43), 134.16 (s, C74), 133.92 (s, C93 and C73), 133.11 (s, C44), 132.96 (s, C41), 132.77 (s, C45), 132.63 (s, C95), 132.28 (s, C91), 132.19 (s, C92), 131.84 (s, C42), 131.66 (s, C72), 131.58 (s, C64), 131.28 (s, C94), 131.22 (s, C71), 131.14 (s, C75), 129.66 (s, C62), 129.21 (s, C63), 128.88 (s, C14), 128.08 (s, C13), 123.97 (s, C12), 123.30 (s, C23), 109.84 (s, C26), 41.23 (s, C25), 28.10 (s, C29), 24.86 (s, C30), 20.63 (s, C76), 19.72 (s, C96), 19.64 (s, C46), 19.28 (s, C80), 17.90 (s, C50), 17.11 ((s, C48 and C100), 17.09 (s, C49), 17.04 (s, C77), 17.02 (s, C98), 16.83 (s, C78), 16.80 (s, C47), 16.57 (s, C79), 16.33 (s, C99), 16.24 (s, C97), 15.99 (s, C28), 14.70 (s, C27) ppm. **HRMS ESI (m/z):** $[M+H]^+$ calculated for $C_{56}H_{66}As$ 813.43750; found 813.43640.

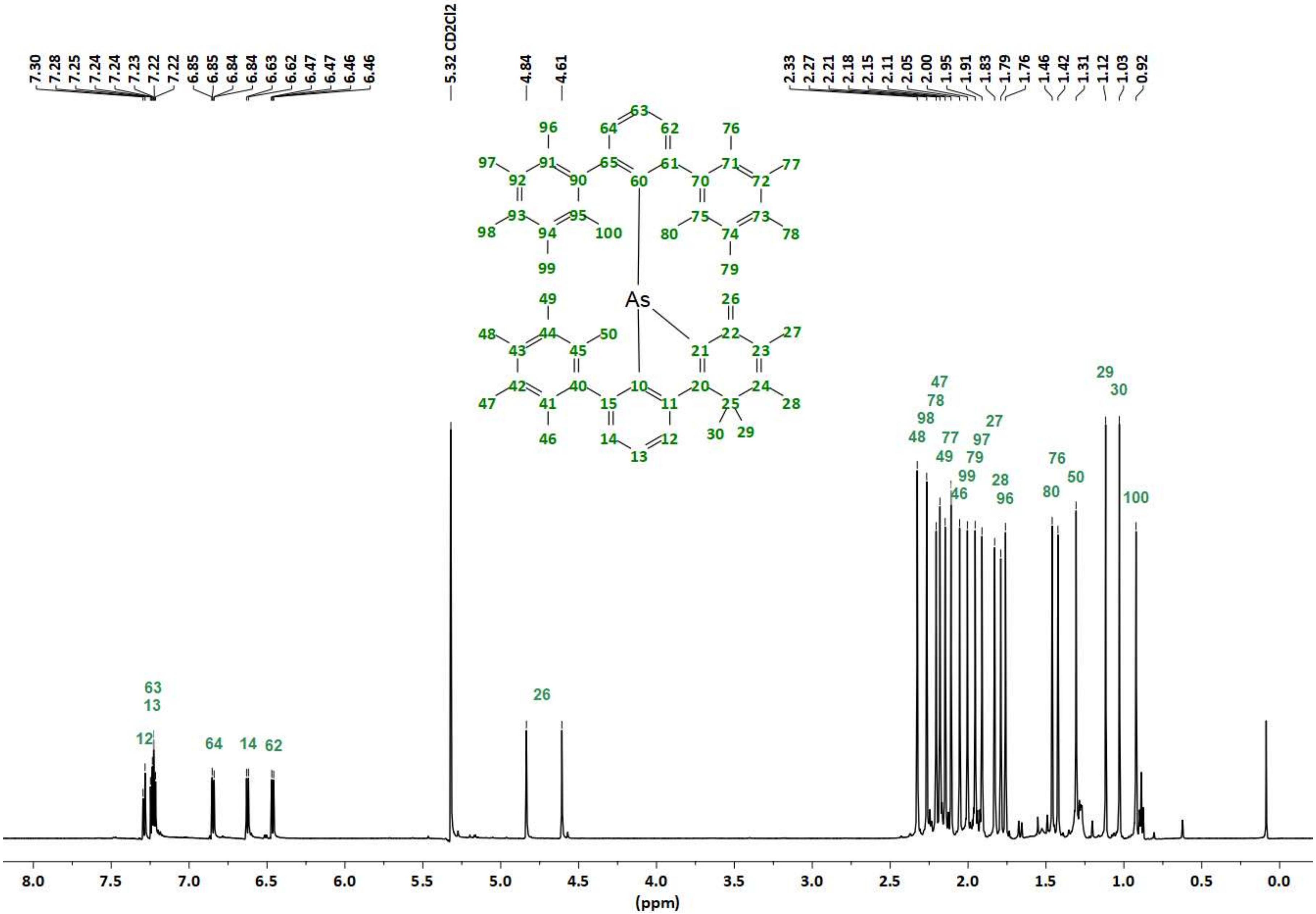

Figure S53. ^{1}H NMR (CD_2Cl_2, 600 MHz) spectrum (full) of **9b**.

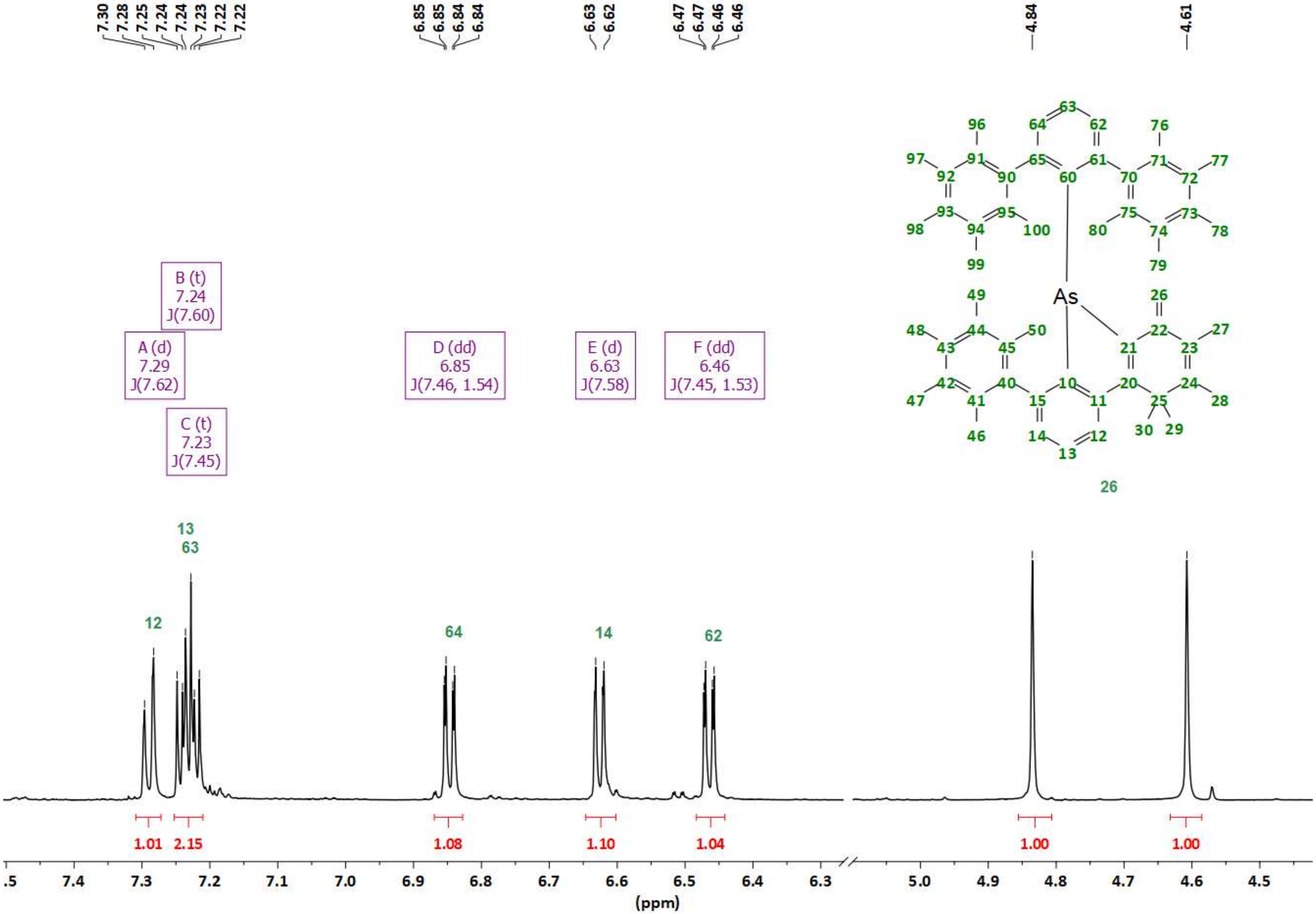

Figure S54. ^{1}H NMR (CD_2Cl_2, 600 MHz) spectrum (detail) of **9b**.

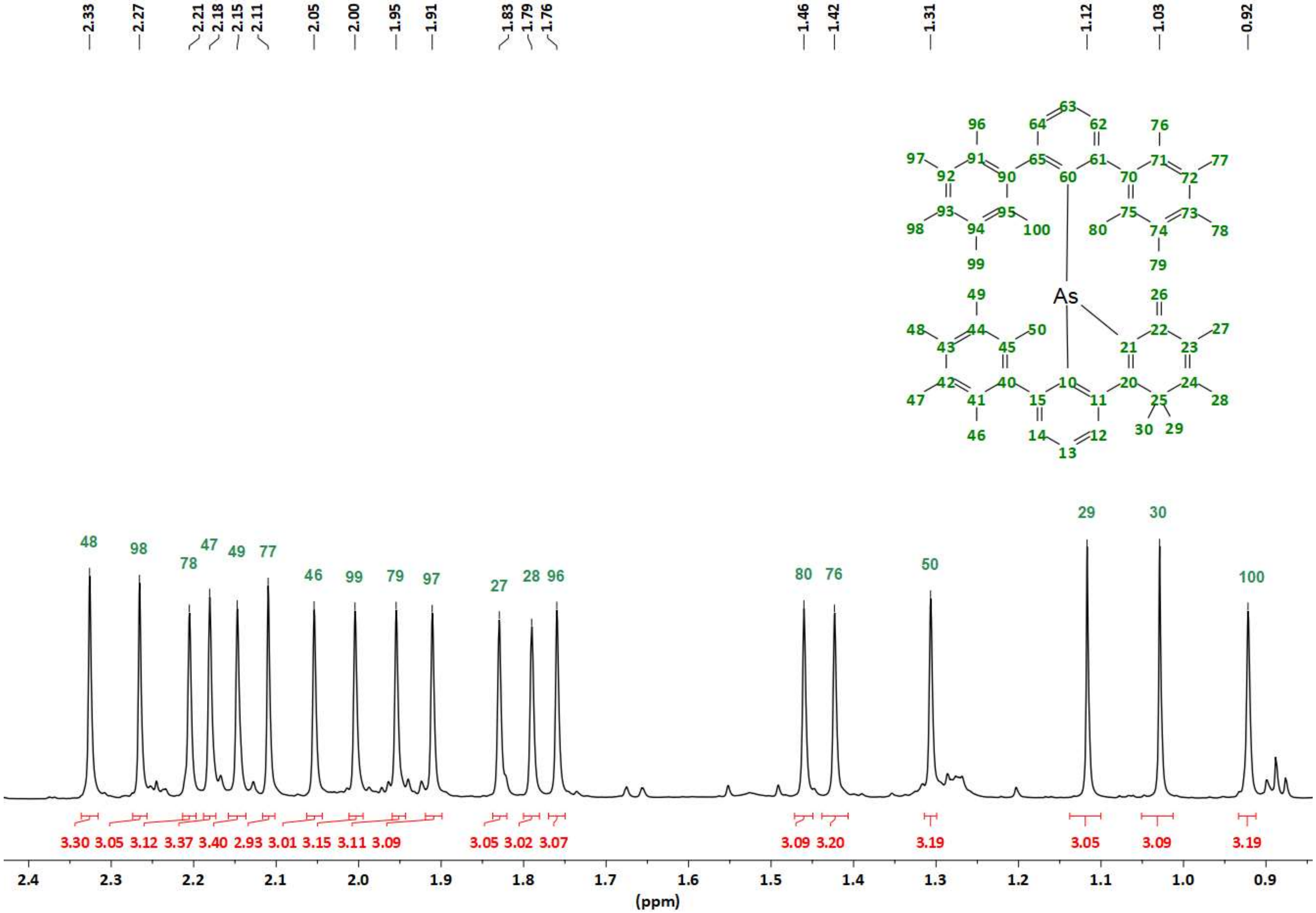

Figure S55. ^{1}H NMR (CD_2Cl_2, 600 MHz) spectrum (detail) of **9b**.

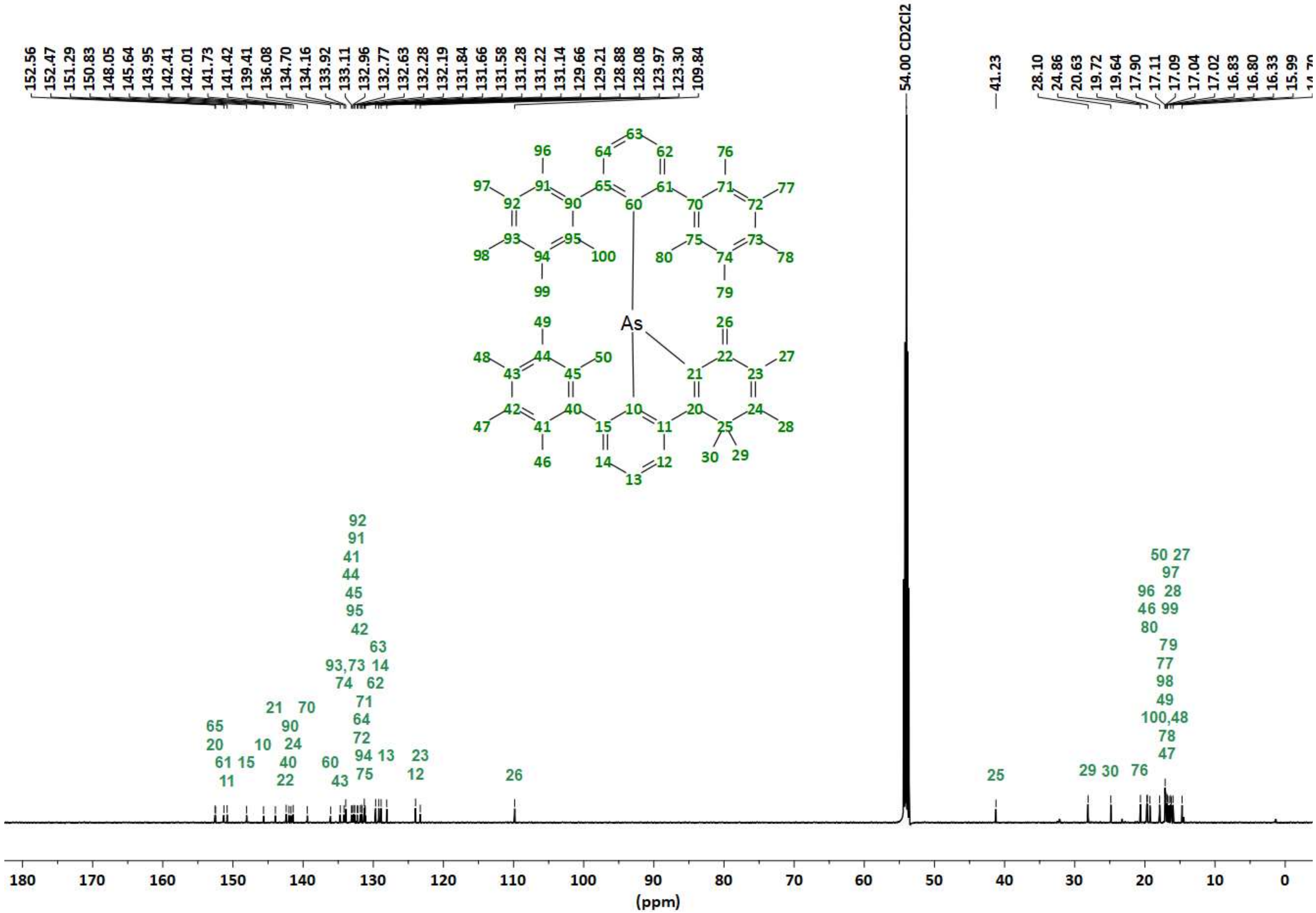

Figure S56. $^{13}C\{^{1}H\}$ NMR (CD_2Cl_2, 151 MHz) spectrum (full) of **9b**.

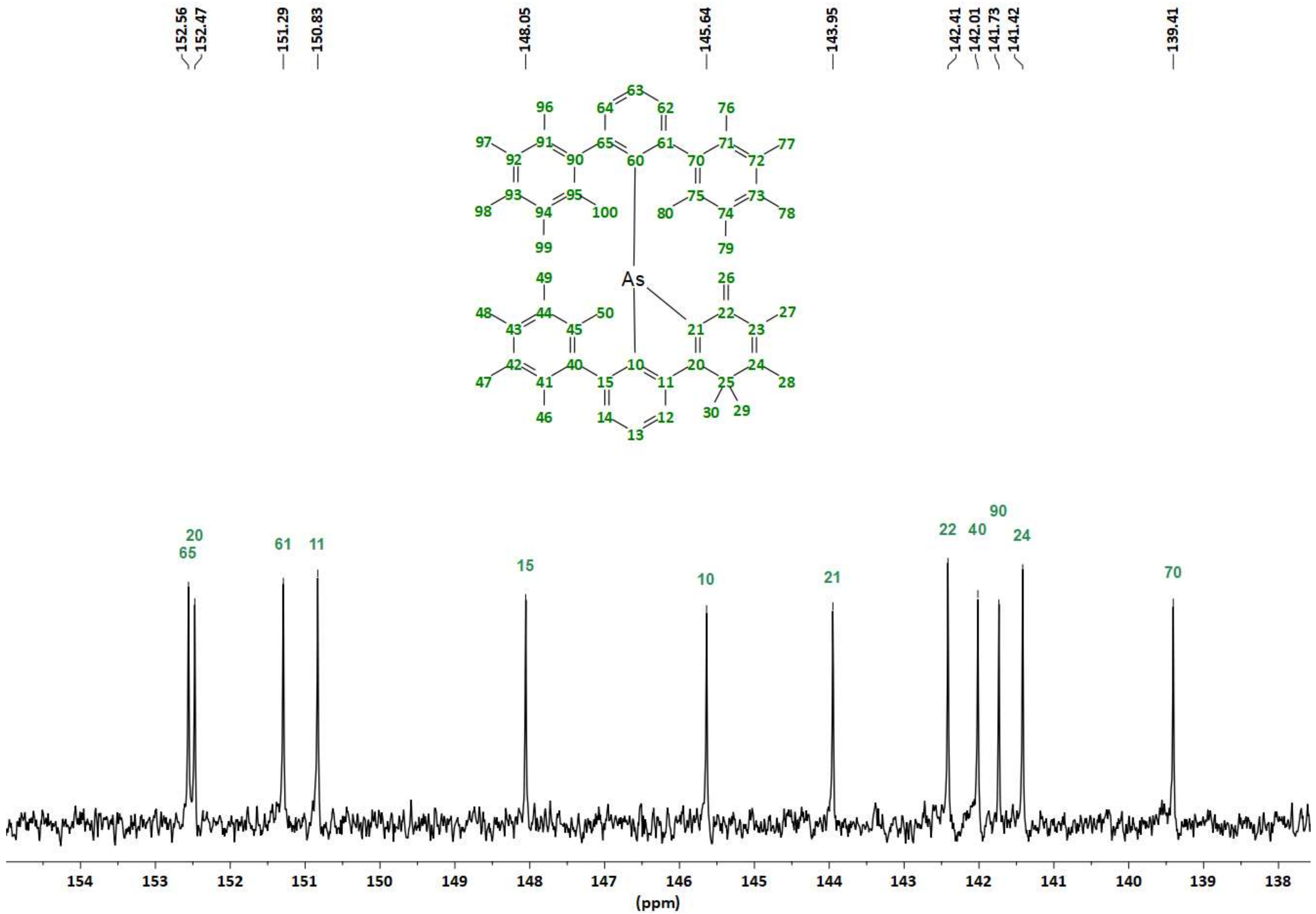

Figure S57. $^{13}C\{^{1}H\}$ NMR (CD_2Cl_2, 151 MHz) spectrum (detail) of **9b**.

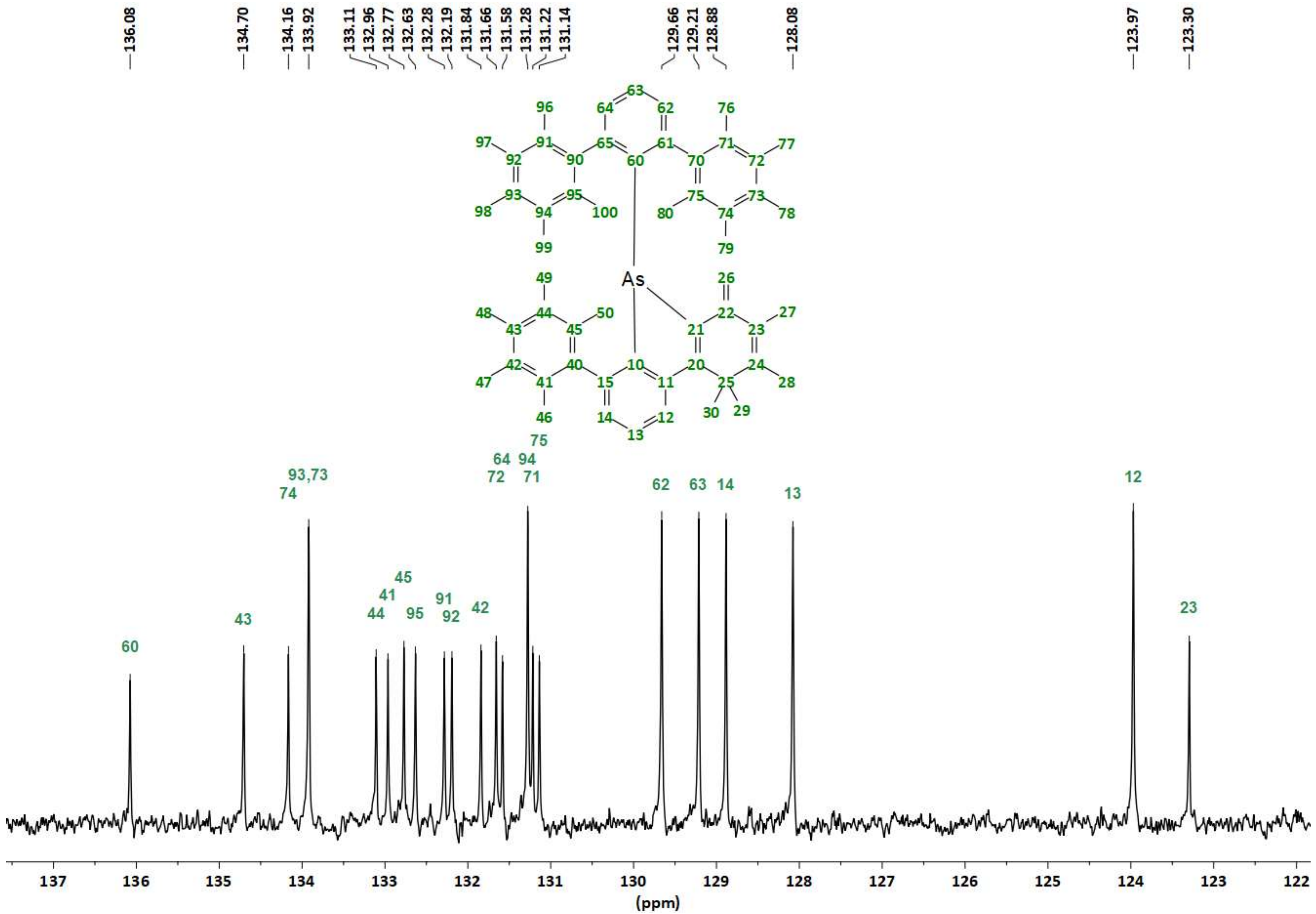

Figure S58. $^{13}C\{^{1}H\}$ NMR (CD_2Cl_2, 151 MHz) spectrum (detail) of **9b**.

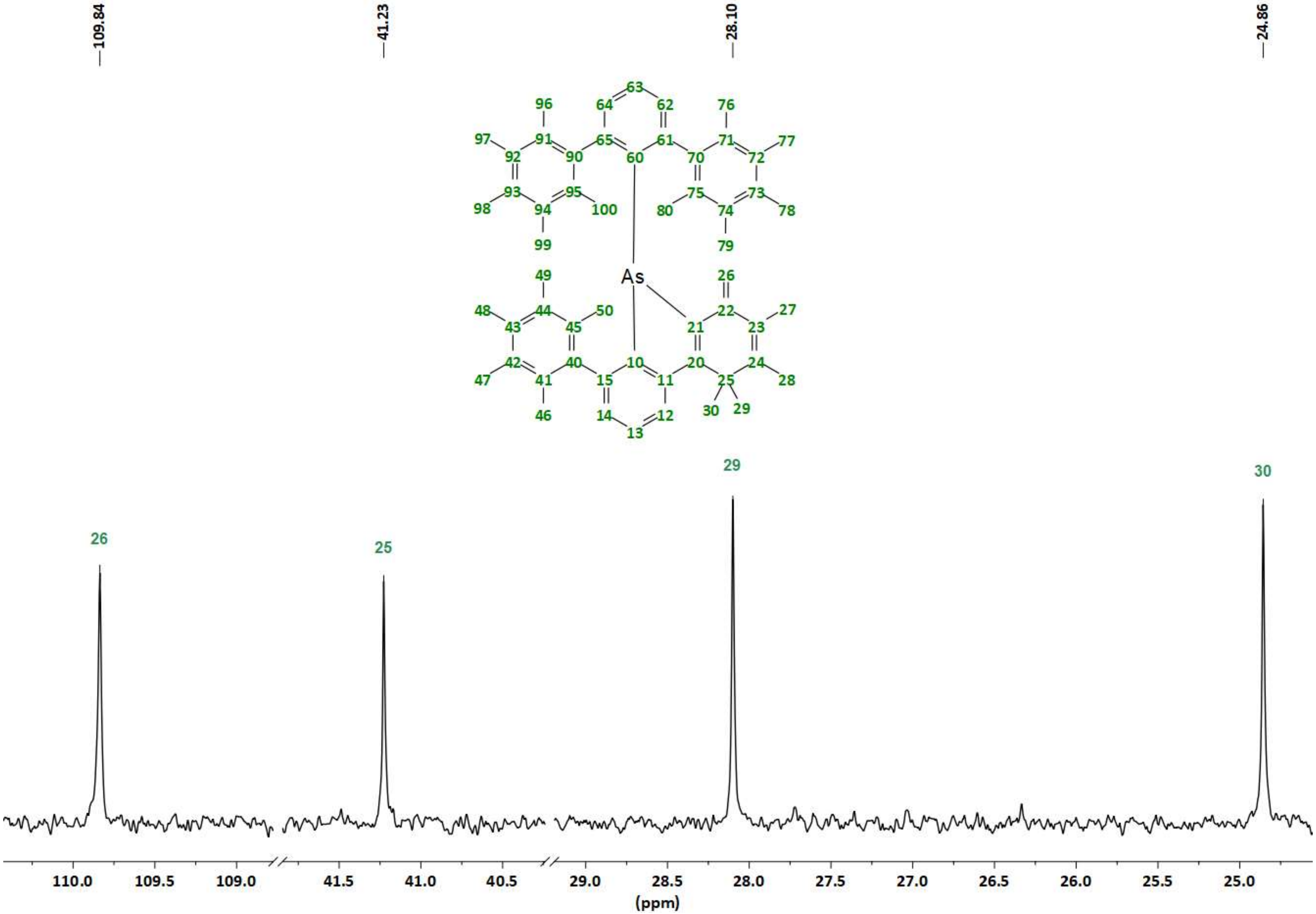

Figure S59. $^{13}C\{^{1}H\}$ NMR (CD_2Cl_2, 151 MHz) spectrum (detail) of **9b**.

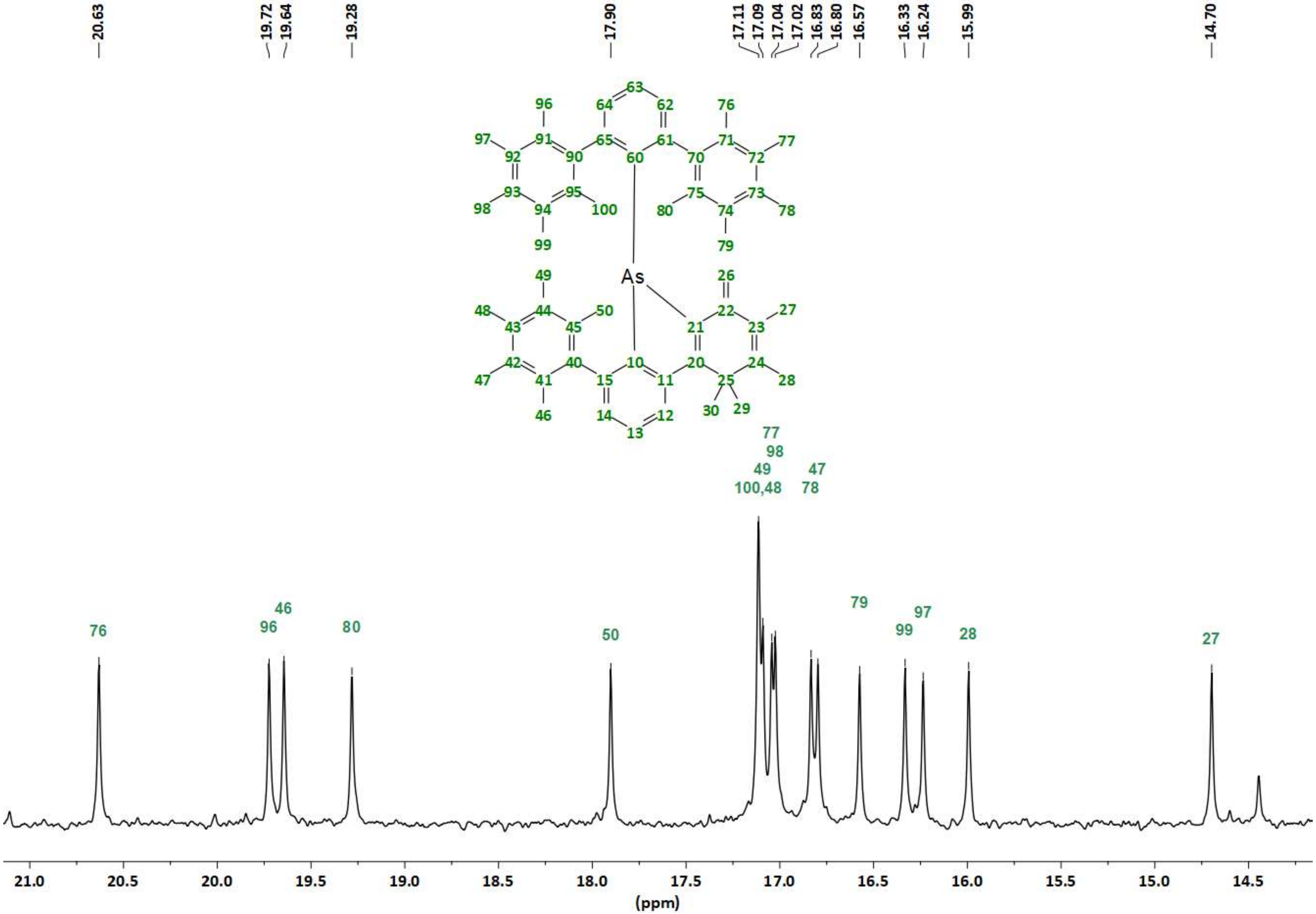

Figure S60. $^{13}C\{^1H\}$ NMR (CD_2Cl_2, 151 MHz) spectrum (detail) of **9b**.

X-Ray diffraction studies

Intensity data of **1a**, **1b**, [**5a**][0.75·$AlCl_4$+0.25·$EtAlCl_3$], [**5b**][$AlCl_4$], [**8a**][$AlCl_4$]·CH_2Cl_2, **9a** and **9b** was collected on a Bruker Venture D8 diffractometer at 100 K with graphite-monochromated Mo-Kα (0.7107 Å) radiation. All structures were solved by direct methods and refined based on F^2 by use of the SHELX program package as implemented in WinGX.[S3,S4] All non-hydrogen atoms were refined using anisotropic displacement parameters. Hydrogen atoms attached to carbon atoms were located form the difference Fourier map and refined freely for **1a**. For [**5a**][0.75·$AlCl_4$+0.25·$EtAlCl_3$], [**5b**][$AlCl_4$], [**8a**][$AlCl_4$]·CH_2Cl_2, **9a**, and **9b** H atoms were treated as a mixture of freely refined and geometrically constrained positions within the ridding model.

The diffraction pattern measured during data collection for crystals of **1b** revelled a non-merohedral twin with a two-fold twin law. Unfortunately, the crystal quality and scattering power were not enough to obtain sufficient resolution to provide a high quality structure. Despite the fact that the twin law solve most problems during the structure refinement, there are still many reflection overlaps that were not properly de-convoluted by the twin law solely, but most importantly the derived weak intensities for the highest resolution shells (0.93–0.75A) prevent a better model to be presented, as it can be seen from the large derived K factors of 13.326. Crystal and refinement data are collected in Tables S1 and S2. Figures were created using DIAMOND.[S5] Crystallographic data for the structural analyses have been deposited with the Cambridge Crystallographic Data Centre. Copies of this information may be obtained free of charge from The Director, CCDC, 12 Union Road, Cambridge CB2 1EZ, UK (Fax: +44-1223-336033; e-mail: deposit@ccdc.cam.ac.uk or http://www.ccdc.cam.ac.uk).

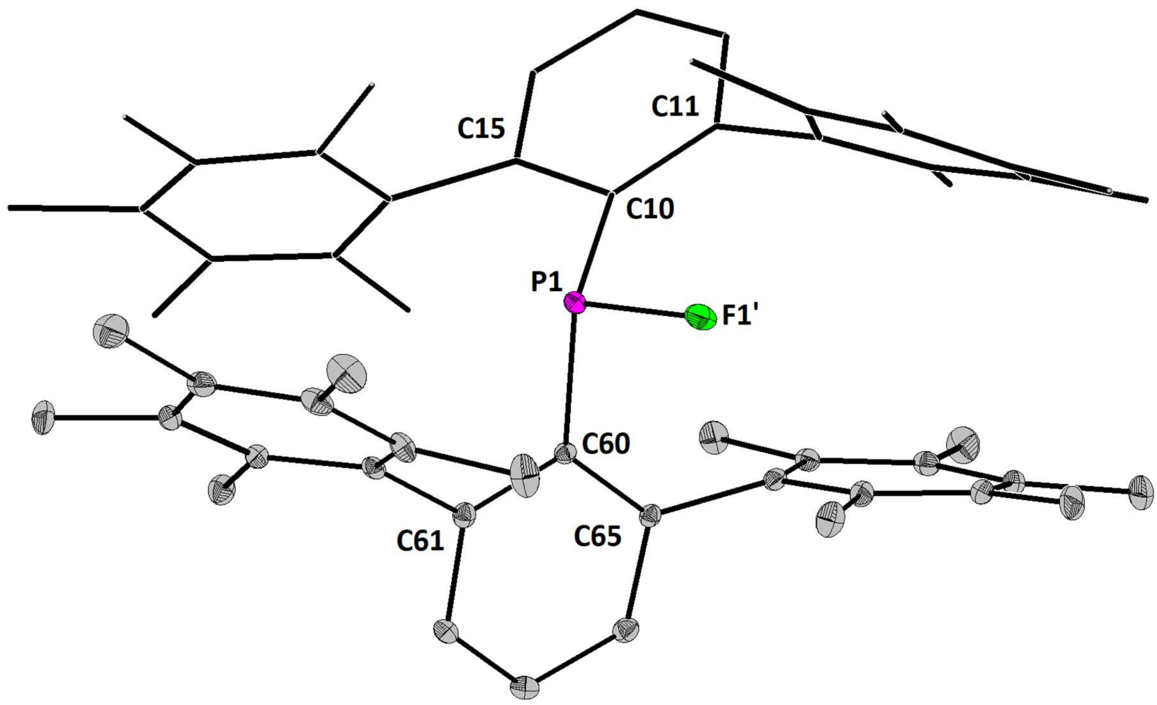

Figure S61. Molecular structure of **1a** showing 30% probability ellipsoids and the essential atom numbering scheme. Selected bond parameters [Å, °] of **1a**: C10–P1 1.853(3), C60–P1–1.848(2), F1'–P1–1.602(3), F1'–P1–C60 96.7(1), F1'–P1–C10 100.1(1), C60–P1–C10 111.5(1), C15–C10–P1 114.7(1), C11–C10–P1 125.2(2), C61–C60–P1 113.1(1), C65–C60–P1 128.0(2).

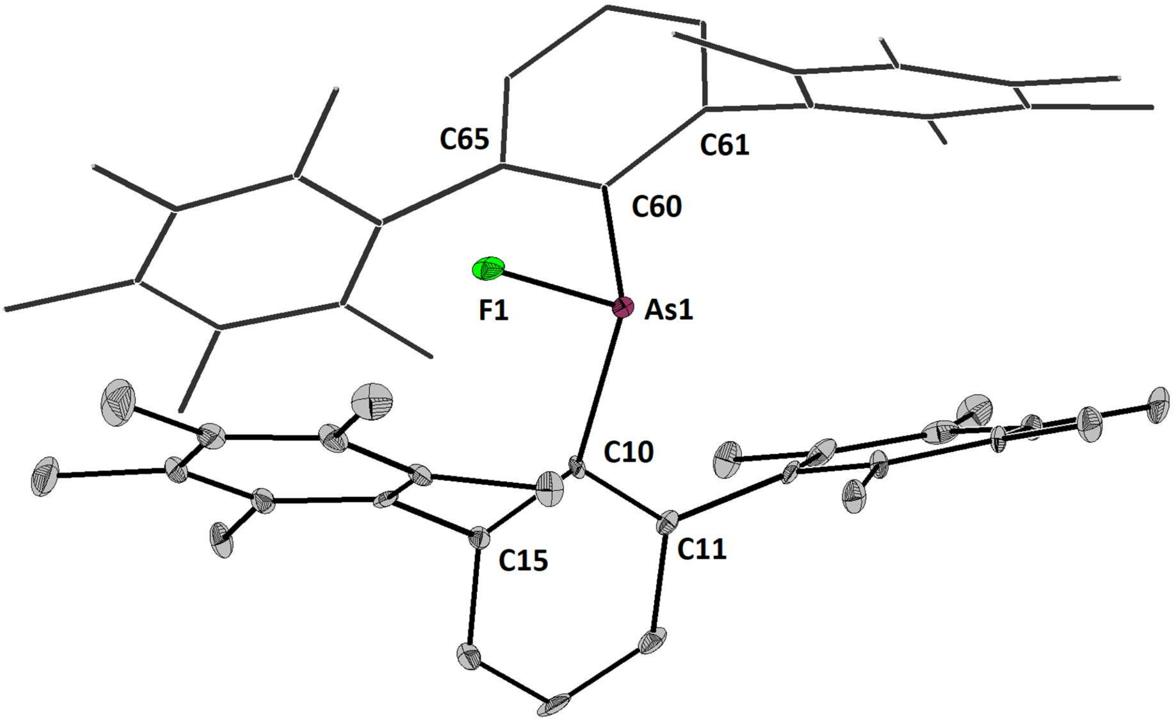

Figure S62. Preliminary molecular structure of **1b** showing 30% probability ellipsoids and the essential atom numbering scheme.

Table S1. Crystal data and structure refinement of **1a**, **1b**, [**5a**][0.75·$AlCl_4$+0.25·$EtAlCl_3$].

	**1a**	**1b**	[**5a**][0.75·$AlCl_4$+0.25·$EtAlCl_3$]
Formula	$C_{56}H_{66}FP$	$C_{56}H_{66}AsF$	$C_{56}H_{66}P{\cdot}C_{0.52}H_{1.29}AlCl_{3.74}$
Formula weight, g mol^{-1}	789.05	833	937.19
Crystal system	Triclinic	Orthorhombic	Triclinic
Crystal size, mm	0.2×0.2×0.2	0.6×0.4×0.2	0.08×0.07×0.07
Space group	$P\bar{1}$	Pbca	$P\bar{1}$
a, Å	12.4535(6)	15.901(1)	11.1666(4)
b, Å	12.5560(6)	16.105(1)	11.6683(4)
c, Å	16.3309(8)	34.642(2)	19.2087(7)
α, °	68.0885(15)	90	92.0876(13)
β, °	71.6618(16)	90	99.9649(13)
γ, °	76.814(16)	90	91.0749(14)
V, Å^3	2230.99(19)	8871.7 (10)	2462.61(15)
Z	2	8	2
ρ_{calcd}, g cm^{-3}	1.175	1.247	1.264
μ (Mo *Kα*), mm^{-1}	0.103	0.81	0.314
F(000)	852	3552	996
θ range, deg	2.3–29.7	2.5–28.3	2.5–29.6
Index ranges	−17 ≤ h ≤ 17<br>−17 ≤ k ≤ 17<br>−22 ≤ l ≤ 22	−21 ≤ h ≤ 21<br>−21 ≤ k ≤ 21<br>−46 ≤ l ≤ 46	−13 ≤ h ≤ 13<br>−13 ≤ k ≤ 13<br>−22 ≤ l ≤ 22
No. of reflns collected	51821	131233	42596
Completeness to θ_{max}	0.997	0.973	0.999
No. indep. Reflns	12661	11523	8669
No. obsd reflns with ($I>2\sigma(I)$)	10005	10667	7281
No. refined params	582	564	668
GooF (F^2)	1.123	1.22	1.05
R_1 (F) ($I > 2\sigma(I)$)	0.071	0.152	0.0417
wR_2 (F^2) (all data)	0.177	0.348	0.0417
Largest diff peak/hole, e Å^{-3}	0.5/−0.35	5.28/−2.43	0.7/−0.62
CCDC number	1993654	not deposited	1993655

Table S2. Crystal data and structure refinement of [**5b**][$AlCl_4$], [**8a**][$AlCl_4$]·CH_2Cl_2, **9a** and **9b**.

	[**5b**][$AlCl_4$]	[**8a**][$AlCl_4$]·CH_2Cl_2	**9a**	**9b**
Formula	$C_{56}H_{66}As·AlCl_4$	$C_{56}H_{65.68}Cl_{0.05}P·AlCl_4·CH_2Cl_2$	$C_{56}H_{65}P$	$C_{56}H_{65}As$
Formula weight, g mol^{-1}	982.78	1025.22	769.05	813.01
Crystal system	Triclinic	Triclinic	Monoclinic	Triclinic
Crystal size, mm	0.36×0.31×0.17	0.25×0.25×0.25	0.07×0.07×0.05	0.29×0.28×0.18
Space group	$P\bar{1}$	$P\bar{1}$	$P2_1/c$	$P\bar{1}$
a, Å	11.1651(4)	12.1874(3)	15.1170(6)	12.0629(6)
b, Å	11.7015(5)	15.1040(4)	19.9711(7)	12.2645(7)
c, Å	19.3134(7)	15.5399(4)	14.8307(6)	16.1347(7)
α, °	92.154(1)	77.572(1)	90	69.928(2)
β, °	100.440(1)	70.442(1)	105.4962(14)	88.099(2)
γ, °	91.535(1)	85.716(1)	90	86.440(2)
V, Å^3	2478.29 (17)	2632.34(12)	4314.7(3)	2237.6(2)
Z	2	2	4	2
ρ_{calcd}, g cm^{-3}	1.317	1.293	1.184	1.207
μ (Mo *Kα*), mm^{-1}	0.956	0.413	0.101	0.80
F(000)	1032	1081	1664	870
θ range, deg	2.5–33.2	2.6–40.2	2.5–28.3	2.2–33.1
Index ranges	−17≤ h ≤ 17<br>−18 ≤ k ≤ 17<br>−24 ≤ l ≤ 24	−19 ≤ h ≤ 19<br>−23 ≤ k ≤ 23<br>−24 ≤ l ≤ 24	−20 ≤ h ≤ 20<br>−26 ≤ k ≤ 26<br>−19 ≤ l ≤ 19	−18 ≤ h ≤ 18<br>−18 ≤ k ≤ 18<br>−24 ≤ l ≤ 24
No. of reflns collected	85724	211012	94259	76408
Completeness to θ_{max}	0.998	0.999	0.999	0.995
No. indep. Reflns	19004	22020	10802	17063
No. obsd reflns with (*I*>2σ(*I*))	16307	18729	8556	13656
No. refined params	625	690	584	565
GooF (F^2)	1.03	1.04	1.08	1.06
R_1 (*F*) (*I* > 2σ(*I*))	0.033	0.040	0.0377	0.048
wR_2 (F^2) (all data)	0.088	0.121	0.067	0.118
Largest diff peak/hole, e Å^{-3}	0.52/−0.41	1.30/−1.08	1.18/−0.36	0.85/−0.47
CCDC number	1993656	1993657	1993658	1993659

Computational data

The structures of all cations were optimized in the gas-phase by density functional theory (DFT) at the B3PW91/6-311+G*[S6,S7] level of theory using Gaussian09.[S8] The starting geometries were modelled at the computer with GaussView 5. Dispersion effects were accounted for using the empirical Grimme correction (GD3BJ).[S9]

References

S1. S. Hino, M. M. Olmstead, J. C. Fettinger, P. P. Power, *J. Organomet. Chem.*, **2005**, *690*, 1638–1644.

S2. G. R. Fulmer, A. J. M. Miller, N. H. Sherden, H. E. Gottlieb, A. Nudelman, B. M. Stoltz, J. E. Bercaw, K. I. Goldberg, *Organometallics* **2010**, *29*, 2176–2179.

S3. G. M. Sheldrick, *Acta Cryst*. **2008**, *A64*, 112-122.

S4. L. Farrugia, *J. Appl. Cryst*. **1999**, *32*, 837-838.

S5. K. Brandenburg, Diamond, version 4.0.4, Crystal Impact GbR: Bonn, Germany, **2012**.

S6. J. P Perdew, J. A. Chevary, S. H. Vosko, K. A. Jackson, M. R. Pederson, D. J. Singh, C. Fiolhais, *Phys. Rev*. B **1992**, *46*, 6671-6687.

S7. A. D. Becke, *J. Chem. Phys*. **1993**, *98*, 5648-5652.

S8. M. J. Frisch, G. W. Trucks, H. B. Schlegel, G. E. Scuseria, M. A. Robb, J. R. Cheeseman, G. Scalmani, V. Barone, B. Mennucci, G. A. Petersson, H. Nakatsuji, M. Caricato, X. Li, H. P. Hratchian, A. F. Izmaylov, J. Bloino, G. Zheng, J. L. Sonnenberg, M. Hada, M. Ehara, K. Toyota, R. Fukuda, J. Hasegawa, M. Ishida, T. Nakajima, Y. Honda, O. Kitao, H. Nakai, T. Vreven, J. A. Montgomery, Jr., J. E. Peralta, F. Ogliaro, M. Bearpark, J. J. Heyd, E. Brothers, K. N. Kudin, V. N. Staroverov, R. Kobayashi, J. Normand, K. Raghavachari, A. Rendell, J. C. Burant, S. S. Iyengar, J. Tomasi, M. Cossi, N. Rega, J. M. Millam, M. Klene, J. E. Knox, J. B. Cross, V. Bakken, C. Adamo, J. Jaramillo, R. Gomperts, R. E. Stratmann, O. Yazyev, A. J. Austin, R. Cammi, C. Pomelli, J. W. Ochterski, R. L. Martin, K. Morokuma, V. G. Zakrzewski, G. A. Voth, P. Salvador, J. J. Dannenberg, S. Dapprich, A. D. Daniels, Ö. Farkas, J. B. Foresman, J. V. Ortiz, J. Cioslowski, D. J. Fox, Gaussian 09, Revision B.01, Gaussian Inc., Wallingford CT, **2010**.

S9. S. Grimme, J. Antony, S. Ehrlich and H. Krieg, *J. Chem. Phys*., **2010**, *132*,154104.
